# A phase II peri-operative study of pembrolizumab plus lenvatinib for mucosal melanoma

**DOI:** 10.1038/s41467-026-73190-1

**Published:** 2026-05-16

**Authors:** Lili Mao, Yumei Lai, Hong Zheng, Ming Cui, Lifeng Li, Ziyi Liu, Hongyu Zhou, Linzi Sun, Caili Li, Xiaoting Wei, Junjie Gu, Xue Bai, Yan Kong, Chuanliang Cui, Zhihong Chi, Xinan Sheng, Bin Lian, Siming Li, Xieqiao Yan, Bixia Tang, Juan Li, Li Zhou, Xuan Wang, Jun Guo, Jie Dai, Lu Si

**Affiliations:** 1 Key Laboratory of Carcinogenesis and Translational Research (Ministry of Education/Beijing), Department of Melanoma and Sarcoma, Peking University Cancer Hospital and Institute, Beijing, China; 2 Key Laboratory of Carcinogenesis and Translational Research (Ministry of Education/Beijing), Department of Pathology, Peking University Cancer Hospital and Institute, Beijing, China; 3 Key Laboratory of Carcinogenesis and Translational Research (Ministry of Education/Beijing), Department of Gynaecology, Peking University Cancer Hospital and Institute, Beijing, China; 4 Key Laboratory of Carcinogenesis and Translational Research (Ministry of Education/Beijing), Department of Gastrointestinal Surgery IV, Peking University Cancer Hospital and Institute, Beijing, China; 5 Geneplus-Beijing, Beijing, China; 6 Key Laboratory of Carcinogenesis and Translational Research (Ministry of Education/Beijing), Department of Genitourinary Oncology, Peking University Cancer Hospital and Institute, Beijing, China

**Keywords:** Cancer immunotherapy, Tumour biomarkers

## Abstract

Mucosal melanoma is an aggressive malignancy with limited neoadjuvant options. We conducted a single arm, phase II study of neoadjuvant pembrolizumab plus lenvatinib followed by surgery and adjuvant pembrolizumab in resectable mucosal melanoma (NCT04622566) along with exploratory biomarker analysis. Primary objective was pathological complete response (pCR) rate; secondary endpoints included relapse-free survival (RFS), overall survival (OS), clinical response, surgical outcomes, and safety. Among 21 surgical patients, the pCR rate was 9.5%, major pathologic response (MPR) rate was 19.0%, and the pathologic response rate was 38.1%. Median RFS was 14.8 months (1-year rate: 61.9%); median OS was not reached. No grade 4–5 treatment-related toxicities or additional perioperative complications occcurred. Spatial profiling revealed a more immune-inflamed baseline microenvironment in responders, with activated CD4⁺/CD8⁺ T cell signatures associated with favorable outcomes. Treatment induced vascular normalization and increased T cell infiltration in non-responders, partially narrowing the immune gap. Responders exhibited higher prevalence of persistent TCR clonotypes and tighter spatial proximity between activated CD4⁺ and CD8⁺ T cells. Although the pre-specified primary endpoint was not met, our findings identify activated CD4^+^/CD8^+^ T cell states and TCR persistence as key outcome-associated features, supporting immune-informed optimization of peri-operative therapy in mucosal melanoma.

## Abstract (web summary)

Mucosal melanoma is a rare and aggressive subtype which, unlike cutaneous melanoma, shows limited response to immune checkpoint inhibition. Here, the authors report the safety and efficacy of neoadjuvant pembrolizumab (anti-PD-1) and lenvatinib (multi tyrosine kinase inhibition) in patients with resectable mucosal melanoma, along with exploratory biomarker analysis.

## Introduction

Mucosal melanoma (MM) is an uncommon and clinically challenging form of melanoma that originates from melanocytes located in mucosal tissues. It accounts for 1–2% of melanomas in Caucasian populations but represents ~20% of cases in Asian cohorts^1^. Due to its insidious onset and high invasive potential, nearly half of MM cases are diagnosed with metastatic disease^2^. While ultraviolet radiation (UVR) from sun exposure plays a major role in the development of cutaneous melanoma (CM), the molecular drivers and spatial immunogenomic landscape of MM remain poorly defined^3–6^.

While the introduction of immune checkpoint inhibitors (ICIs) has significantly altered therapeutic strategies for CM, clinical outcomes for MM remain dismal. In chemotherapy-refractory MM, pembrolizumab achieved a modest objective response rate (ORR) of 13.3% (NCT02821000)^7,8^. Pooled post hoc analyses of 84 advanced MM patients across KEYNOTE studies reported a 19% response, alongside a median progression-free survival (mPFS) of 2.3 months and a median overall survival (mOS) of 11.3 months^9^. The limitations of current treatments highlight the necessity for more potent therapeutic options for MM.

Surgery remains the primary treatment approach for resectable melanoma, yet recurrence rates post-resection are high^10,11^. Neoadjuvant immunotherapy has shown survival benefits in CM, particularly among patients with limited tumor burden^12–14^. The phase II S1801 trial demonstrated significantly longer event-free survival (EFS) in those treated with neoadjuvant versus adjuvant immunotherapy^12^. Similarly, the phase III NADINA trial confirmed these findings, reporting a hazard ratio of 0.32 for EFS^13^. Neoadjuvant therapy not only reduces recurrence risk and improves surgical outcomes but also enables early evaluation of treatment response^15^. For MM patients, organ preservation is critical, as tumors frequently arise in anatomically sensitive regions like the nasal cavity. However, the application of neoadjuvant immunotherapy in MM remains underexplored.

Due to the restricted benefit of anti–PD-1 treatment alone in MM, combination strategies are being investigated. Angiogenic signaling, particularly through vascular endothelial growth factor (VEGF) and its receptors (VEGFR), contributes to immune evasion and tumor progression. Vascular normalization via VEGF(R) blockade has been shown to facilitate T-cell infiltration and enhance antitumor immunity^16,17^. Inhibitors such as bevacizumab, apatinib, and lenvatinib have demonstrated antitumor activity in MM^18–20^. Combination regimens integrating VEGF(R) inhibitors with ICIs have yielded encouraging results: atezolizumab plus bevacizumab achieved a 45% ORR in first-line metastatic MM; camrelizumab plus apatinib and toripalimab plus axitinib reported ORRs of 42.9% and 48.3%, respectively^21–24^. These findings support further investigation of VEGF(R)–ICI combinations in the neoadjuvant setting.

In this study, we report the results of a phase II, single-arm, open-label trial evaluating peri-operative pembrolizumab plus lenvatinib in patients with resectable MM. In addition to assessing clinical efficacy and safety, we performed whole exome sequencing, spatial transcriptomic profiling, multiplex immunohistochemistry, and TCR sequencing to elucidate the immunologic mechanisms underlying response. Our findings highlight the prognostic relavance of activated CD4⁺/CD8⁺ T cell features and suggest that immune contexture may inform individualized treatment duration. These insights offer a framework for tailoring peri-operative therapy in mucosal melanoma.

## Results

### Patients disposition and baseline characteristics

**Fig. 1:**
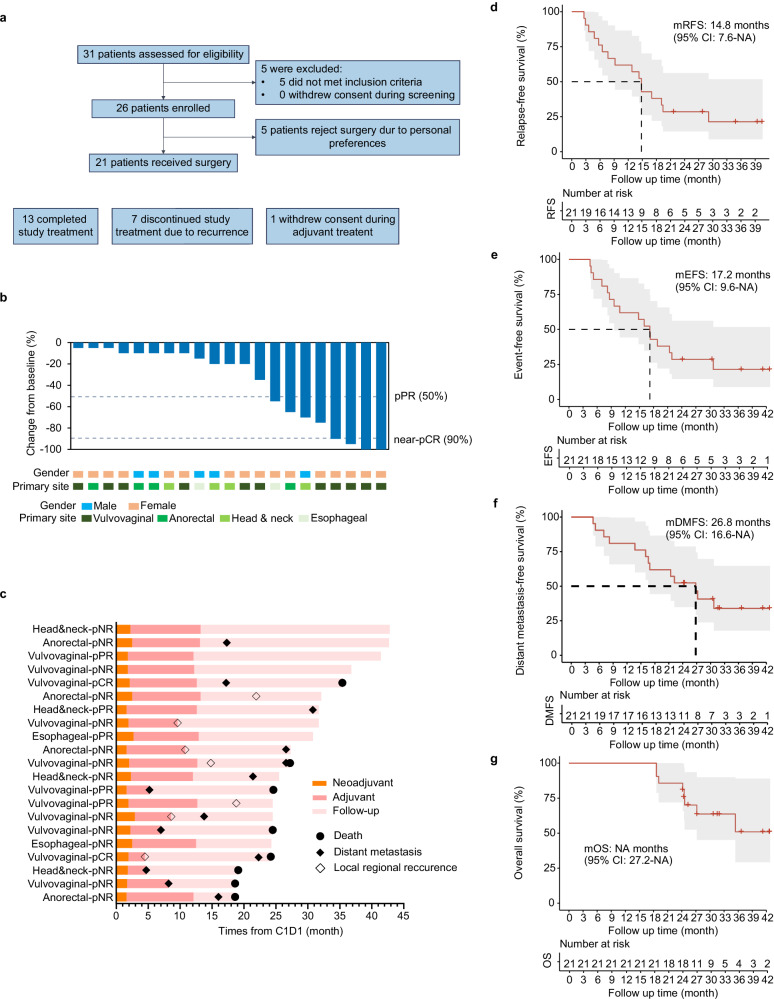
Clinical outcomes. **a** The flowchart of clinical trial. **b**–**g** Clinical efficacy of patients underwent surgery (*n* = 21). **b** Pathological response in primary tumor. pCR (pathological complete response), no viable tumor; near pCR, ≤ 10% viable tumor; pPR (pathological partial response), 11-50% viable tumor; pNR (pathological non-response), >50% viable tumor. **c** Exposure and duration of response per primary sites. **d** relapse-free survival, **e** event-free survival, **f** distant metastasis-free survival, and (**g**) overall survival curves in surgical patients. Survival curves were estimated using the Kaplan–Meier method. The number of patients at risk is provided below the *x*-axis. Source data are provided as a Source Data file.

Between September 2021 and April 2023, 26 patients were enrolled in the study, of whom 7 were males and 19 were females (Fig. 1a). Primary tumor sites included the vulvovaginal region in 11 patients (42.3%), anorectal area in 8 (30.8%), head and neck in 5 (19.2%), and esophagus in 2 (7.7%). At enrollment, 15 patients (57.7%) had localized disease confined to the primary site, while 11 (42.3%) presented with regional lymph node involvement. Most patients (92.3%) had an Eastern Cooperative Oncology Group (ECOG) performance status of 0 (Table 1).

**Table 1:** Patients baseline characteristics

Characteristics	Total participatents (*n* = 26)
Mean age, years (range)	57 (49-75)
Sex, *n* (%)	
Male	7 (26.9)
Female	19 (73.1)
ECOG, *n* (%)	
0	24 (92.3)
1	2 (7.7)
Primary lesion, *n* (%)	
Vulvovaginal	11 (42.3)
Anorectal	8 (30.8)
Head & Neck	5 (19.2)
Esophageal	2 (7.7)
Regional lymph node metastasis, *n* (%)	
Absent	15 (57.7)
Exist	11 (42.3)
LDH, *n* (%)	
<1.5× ULN	23 (88.5)
≥1.5× ULN	3 (11.5)
PD-L1 status, *n* (%)	
CPS ≥ 1	13 (50)
CPS < 1	5 (19.2)
Unknow	8 (30.8)

Abbreviations: LDH lactate dehydrogenase; ULN upper limit of normal; CPS combined positive score.

### Clinical outcomes

Of the 26 enrolled patients (intent-to-treat [ITT] population), 21 proceeded to surgery, while 5 did not undergo planned resection. Among these, four patients declined surgery after experiencing tumor shrinkage and symptom relief. No patient lost the window for curative surgery due to disease progression. The primary endpoint of this study was the pathological complete response (pCR) rate. Among the 21 surgical patients, 2 achieved pCR, yielding a pCR rate of 9.5% (95% CI, 1.2–30.3%) in the surgical cohort and 7.7% (95% CI, 0.9–25.1%) in the ITT population. Regarding other pathological response, 2 patients had near-pCR, 4 had pathological partial response (pPR), and 13 demonstrated pathological non-response (pNR). The secondary endpoint major pathologic response (MPR) rate was 19.0% (95% CI, 5.4–41.9%) in the surgical cohort and 15.4% (95% CI, 4.4–34.9%) in the ITT population. The overall pathological response rate (PRR) was 38.1% (95% CI, 18.1–61.6%) in the surgical cohort and 30.8% (95% CI, 14.3–51.8%) in the ITT population (Table 2; Fig. 1b).

**Table 2:** Pathological response

Pathologic response *n* (%)	ITT population (*n* = 26)	Surgery population (*n* = 21)
pCR	2 (7.7)	2 (9.5)
near-pCR	2 (7.7)	2 (9.5)
pPR	4 (15.4)	4 (19.0)
pNR	13 (50.0)	13 (61.9)
Not evaluable^a^	5 (19.2)	-
Pathologic response rate (95% CI)	30.8% (14.3-51.8%)	38.1% (18.1-61.6%)

^a^Data are n (%) or otherwise specified. Abbreviations: pCR (pathological complete response), no viable tumor; near pCR, ≤ 10% viable tumor; pPR (pathological partial response), 11-50% viable tumor; pNR (pathological non-response), >50% viable tumor.

Clinical response was assessed as a secondary outcome. Since mucosal lesions are non-measurable lesions, most patients in this study only had non-measurable lesions. For those patients with no measurable disease, the sum of diameters of the largest tumor(s) that were not a lymph node and of the shortest diameter of the involved lymph nodes (maximum of two per organ) was used to assess percentage change in tumor size. Percentage change in tumor size before and after neoadjuvant therapy were available for 18 patients,within these 18 patients, including 1 CR, 2 PR, 13 stable disease (SD), and 2 progressive disease (PD). No PD was observed during the neoadjuvant phase. Overall, tumor shrinkage was seen in 12 of 18 patients (66.7%). Representative imaging for three patients is shown in Supplementary Fig. 1.

By the cutoff date of April 1st, 2025, the median follow-up duration was 27.2 months (range 18.6–42.8 months). A total of 16 patients experienced disease relapse: 6 had locoregional relapse, 4 regional recurrence, and 6 developed distant metastases (Fig. 1c). In the surgical population, the secondary outcome of median relapse-free survival (RFS) was 14.8 months (95% CI, 7.6–NA months) (Fig. 1d), with a 1-year RFS rate of 61.9% (95% CI, 44.3–86.6%). RFS did not differ significantly across primary tumor sites (anorectal, vulvovaginal, head and neck, or esophageal) (*p* = 0.25). Median RFS for pathological responders was 15.9 months (95% CI, 14.8–NA months), and 12.8 months (95% CI, 6.5–NA months) for non-responders (*p* = 0.68). The median EFS was 17.2 months (95% CI, 9.6–NA months) (Fig. 1e), and the median distant metastasis-free survival (DMFS) was 26.8 months (95% CI, 16.6–NA months) (Fig. 1f). The median OS, also a secondary outcome, was not reached and the data remain immature at the time of this analysis (Fig. 1g).

### Safety

Safety was evaluated in all 26 patients administered at least one dose of pembrolizumab or lenvatinib, and all patients (100%) experienced treatment-related adverse event (TRAE) (Table 3). The most common TRAEs were hypertension (*n* = 13; 50.0%), proteinuria (*n* = 12; 46.2%), and actate dehydrogenase (LDH) increased (*n* = 9; 34.6%). Most events were grade 1–2, and no grade 4–5 toxicities were observed. Grade 3 TRAEs occurred in three patients and included hypertension, alanine aminotransferase (ALT) increase, and platelet (PLT) count decrease; all resolved following interruption of drug administration or supportive treatment. Besides the three patients with grade 3 TRAEs, six patients underwent temporary interruption of lenvatinib therapy due to grade 1–2 TRAEs, and three of these resumed lenvatinib at a one-level dose reduction after recovery. No patients discontinued pembrolizumab during adjuvant therapy. Surgical resections were performed according to baseline imaging assessments, and no additional perioperative complications were observed. Overall, the combination of pembrolizumab plus lenvatinib demonstrated an acceptable safety profile with manageable toxicities.

**Table 3:** Common (≥10%) treatment-related adverse events from all patients (*n* = 26)

Adverse event *n* (%)	All patients (*n* = 26)	
	Any grade	Grade 3
Treatment - related adverse event	24 (92.3%)	3 (11.5%)
Hypertension	13 (50.0%)	1 (3.9%)
Proteinuria	12 (46.2%)	0
LDH increased	9 (34.6%)	0
Hypothyroidism	8 (30.8%)	0
TG increased	6 (23.1%)	0
Hoarseness	6 (23.1%)	0
ALT increased	5 (19.2%)	1 (3.9%)
GGT increased	5 (19.2%)	0
PLT decreased	5 (19.2%)	1 (3.9)
Rash	4 (15.4%)	0
Hyperglycemia	4 (15.4%)	0
Hand-foot syndrome	3 (11.5%)	0
Hyperuricemia	3 (11.5%)	0
Fatigue	3 (11.5%)	0
WBC decreased	3 (11.5%)	0

Abbreviations: LDH lactate dehydrogenase; TG triglyceride; ALT alanine aminotransferase; GGT gamma-glutamyl transferase; PLT platelet; WBC white blood cell.

### Genomic landscape evolution across neoadjuvant treatment

**Fig. 2:**
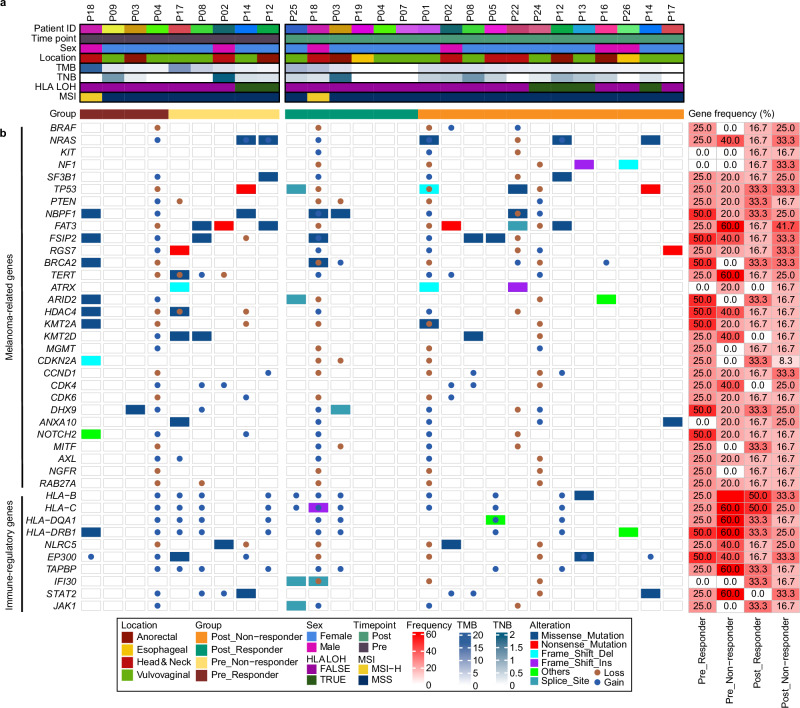
Clinical and genomic characteristics of tumors profiled during neoadjuvant treatment. **a** clinical annotations for pre-treatment biopsies (*n* = 9) and post-treatment surgical specimens (*n* = 18) subjected to whole-exome sequencing, including patient ID, time point, sex, and primary site. Tracks indicate tumor mutational burden (TMB), tumor neoantigen burden (TNB), HLA loss of heterozygosity (HLA LOH), and microsatellite instability (MSI) status. **b** oncoplot summarizing alterations in recurrent melanoma-related genes and selected immune-regulatory genes across the cohort. Source data are provided as a Source Data file.

To investigate molecular correlates of response and resistance to pembrolizumab plus lenvatinib in mucosal melanoma, we profiled longitudinal specimens using whole-exome sequencing (WES), digital spatial profiling (DSP), multiplex immunohistochemistry (mIHC), and TCR sequencing. Supplementary Table 1 summarizes patient-level sample availability and assay coverage across platforms. WES was performed on 9 pre-treatment biopsies (responders, *n* = 4; non-responders, *n* = 5) and 18 post-treatment surgical specimens (responders, *n* = 6; non-responders, *n* = 12) from 19 patients (responders, *n* = 7; non-responders, *n* = 12), including 8 paired pre- and post-treatment cases (Fig. 2a). The landscape of frequently altered melanoma-related genes and immune-regulatory genes (including HLA class I/II genes, *NLRC5*, *EP300*, *TAPBP*, *IFI30*, *STAT2*, and *JAK1*) is summarized in Fig. 2b. Across the cohort, *NBPF1* was among the most frequently altered genes, detected in 4 of 19 patients (21.1%). In paired pre- and post-treatment samples, *NRAS* alteration (P12 and P14) and an *NBPF1* alteration (P18) were maintained after two treatment cycles. The median tumor mutational burden (TMB) was 2.73 mutations/Mb (range, 1.08–16.72) in pre-treatment samples and 1.65 mutations/Mb (range, 0.00–7.53) in post-treatment samples; TMB decreased in 7 of 8 paired cases (87.5%) after neoadjuvant treatment. Notably, HLA loss of heterozygosity (HLA LOH) was observed exclusively in non-responders (4/12 vs. 0/7; *p* = 0.245, Fisher’s exact test), consistent with impaired antigen presentation as a potential resistance mechanism.

### DSP identifies CD4⁺/CD8⁺ T cell signatures associated with response and survival in mucosal melanoma

DSP was performed to comprehensively characterize alterations in the tumor immune microenvironment (TIME) following neoadjuvant pembrolizumab plus lenvatinib. As illustrated in Supplementary Fig. 2a, regions of interest (ROIs) were segmented into two distinct areas of interest (AOIs): the tumor compartment (S100/Pmel⁺) and the immune compartment (CD45⁺). At baseline, 11 ROIs from responders (Pre-R) and 30 ROIs from non-responders (Pre-NR) were analyzed. Following treatment, 31 ROIs from responders (Post-R) and 44 ROIs from non-responders (Post-NR) were sequenced. The number of ROIs per sample, ROI density (ROIs per mm^2^ analyzable area), and ROI coverage (the sum of ROI areas divided by the analyzable tissue area) were comparable between responders and non-responders at both pre-treatment and post-treatment time points, indicating no substantial, response-associated systematic sampling bias (Supplementary Fig. 2b-d).

**Fig. 3:**
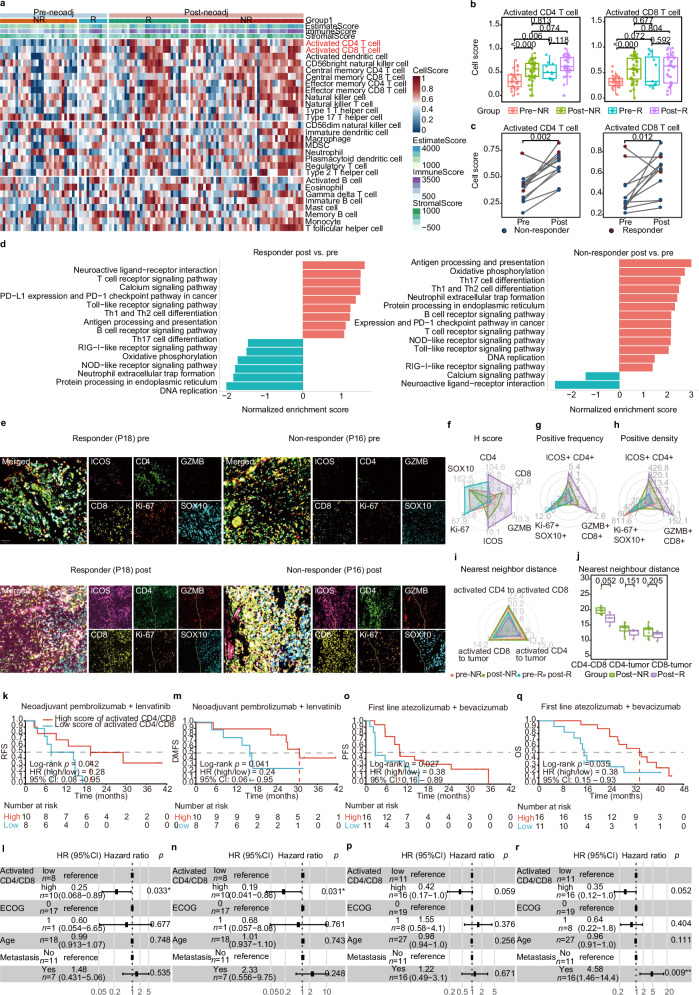
Digital spatial profiling (DSP) of the CD45^+^ compatment and immune dynamics. **a** Heatmap illustrating immune cell infiltration patterns evaluated by ssGSEA in regions of interest (ROIs) from responders and non-responders pre- (pre-NR, *n* = 30; pre-R, *n* = 11) and post-neoadjuvant (post-R, *n* = 31; post-NR, *n* = 44) treatment. **b** Box plot showing scores of activated CD4^+^ T cells and activated CD8^+^ T cells across different groups. *P* values were determined by unpaired Student’s t-test. **c** Scores of activated CD4^+^ T cells and activated CD8^+^ T cells before and after neoadjuvant treatment in paired samples (*n* = 12). The mean signature score was calculated by averaging multiple ROIs from the same specimen. *P* values were determined by two-sided Wilcoxon test. **d** Bar plots displaying enriched KEGG pathways in responders and non-responders when comparing post-neoadjuvant to pre-neoadjuvant treatment. **e**–**j** Multiplex immunohistochemistry analysis of pre- (*n* = 8) and post-neoadjuvant (*n* = 19) treatment specimens. **e** Representative images showing activated CD4^+^ T cells (ICOS^+^CD4^+^), activated CD8^+^ T cells (GZMB^+^CD8^+^), and proliferating melanoma cells (Ki-67^+^SOX10^+^) in paired pre- and post-treatment specimens. Radar plots display the mean values of each marker expression and cell phenotypes by (**f**) H-score, (**g**) positive cell frequency, and (**h**) positive cell density. **i** Nearest-neighbor cell distance (NNC) among indicated cell types within tumor cores across groups. **j** Post-treatment comparison of NNC between responders and non-responders in the tumor core area. *P* values were determined by two-sided Mann-Whitney U test. **k**–**n** Kaplan–Meier plots and multivariable Cox models for (**k, i**) relapse-free survival (RFS) and (**m**, **n**) distant metastasis-free survival (DMFS) in patients stratified by activated CD4^+^ and CD8^+^ T cell score in mucosal melanoma patients received neoadjuvant anti-PD-1 plus VEGFR inhibitor treatment (*n* = 18). **o**–**r** Kaplan–Meier plots and multivariable Cox models for (**o, p**) progression-free survival (PFS) and (**q**, **r**) overall survival (OS) in an external cohort of advanced mucosal melanoma treated with first-line anti–PD-1 plus VEGF(R) inhibitor therapy (NCT04091217), stratified by activated CD4⁺ or activated CD8⁺ T cell scores (*n* = 27). Survival curves were compared using two-sided log-rank tests. For multivariable Cox proportional hazards regression (two-sided), data are presented as hazard ratio (HR) values (measure of centre) with 95% confidence intervals (CI) (error bars). Covariates included activated CD4 + /CD8 + T-cell scores, ECOG, age, and metastasis. For box plots, the center line represents the median, box limits represent lower and upper quartiles, and whiskers extend to minimum and maximum values; individual data points are overlaid. Source data are provided as a Source Data file.

Immune cell phenotypes were quantified using single-sample gene set enrichment analysis (ssGSEA) and compared across response groups and time points. At baseline, non-responders exhibited a “colder” immune phenotype, characterized by significantly lower immune scores and reduced infiltration of key anti-tumor immune cell populations, including activated CD4⁺ T cells, activated CD8⁺ T cells, etc (Fig. 3a, b, Supplementary Fig. 2e). Notably, activated dendritic cell and plasmacytoid dendritic cell signatures were also lower in non-responders at baseline, consistent with a less favorable antigen presentation and T-cell priming milieu prior to therapy. These differences suggest that poor baseline T cell infiltration and compromised antigen presentation may underlie resistance to therapy. Post-treatment, non-responders showed increased infiltration of T cells relative to baseline (Supplementary Fig. 2f), whereas responders exhibited moderate further increases after therapy (Supplementary Fig. 2g). As a result, post-treatment immune cell composition became comparable between groups (Fig. 3b, Supplementary Fig. S2e). Among all evaluated immune populations, activated CD4⁺ (*p* = 0.002) and CD8⁺ T cells (*p* = 0.012) showed the most substantial baseline differences and dynamic treatment-associated increases (Fig. 3b, Supplementary Fig. 2e). Consistent with these shifts, KEGG pathway enrichment analysis of the CD45⁺ compartment revealed a more significant upregulation of immune-related pathways after neoadjuvant therapy in non-responders, including T cell receptor signaling, Toll-like receptor signaling, B cell receptor signaling, and antigen processing and presentation (Fig. 3d), indicating broad immune activation induced by combination therapy.

To provide histologic validation of the transcriptome-derived immune signatures, we performed mIHC using an ICOS/CD4/GZMB/CD8/Ki-67/SOX10 panel (Fig. 3e, f). Activated CD4⁺ T cells were defined as ICOS⁺CD4⁺ cells, activated CD8⁺ T cells as GZMB⁺CD8⁺ cells, and proliferating melanoma cells as Ki-67⁺SOX10⁺ cells. The frequency and density of ICOS⁺CD4⁺ and GZMB⁺CD8⁺ cells increased after treatment in both responders and non-responders, whereas Ki-67⁺SOX10⁺ cells decreased (Fig. 3g, h; and Supplementary Fig. 3). Although not statistically significant due to limited sample numbers, the magnitude of activated CD4⁺ and activated CD8⁺ T-cell increases tended to be greater in post-treatment responders compared to post-treatment non-responders. Spatial proximity analysis based on nearest-neighbor distances (NNC) showed that, within tumor core compartments, responders exhibited shorter NNCs between activated CD4⁺ and CD8⁺ T cells, as well as between these T cell subsets and proliferating melanoma cells post-treatment, indicating a stronger immune–tumor cell interaction (Fig. 3i, j). Together, these data indicate treatment-associated changes in both the abundance and spatial organization of activated T cell subsets.

High post-treatment ssGSEA score of activated CD4⁺/CD8⁺ T cells were associated with significantly longer RFS (*p* = 0.042, log-rank test; Fig. 3k, l) and DMFS (*p* = 0.041, log-rank test; Fig. 3m, n). These survival associations were further validated in an independent, previously published multi-center phase II cohort of unresectable locally advanced or metastatic MM patients treated with first-line atezolizumab plus bevacizumab (NCT04091217; bulk RNA-seq subset available from 27 patients at our center)^22^, in which higher activated CD4⁺/CD8⁺ T cell signature scores associated with improved PFS (*p* = 0.027, log-rank test; Fig. 3o, p) and OS (*p* = 0.035, log-rank test; Fig. 3q, r). Together, these observations suggest a consistent relationship between activated CD4⁺/CD8⁺ T cell states and benefit under combined ICI and VEGF(R) inhibition in mucosal melanoma, supporting these features as a candidate outcome-associated biomarkers.

### Neoadjuvant therapy induces tumor immunogenicity and vascular remodeling in initially cold tumors

**Fig. 4:**
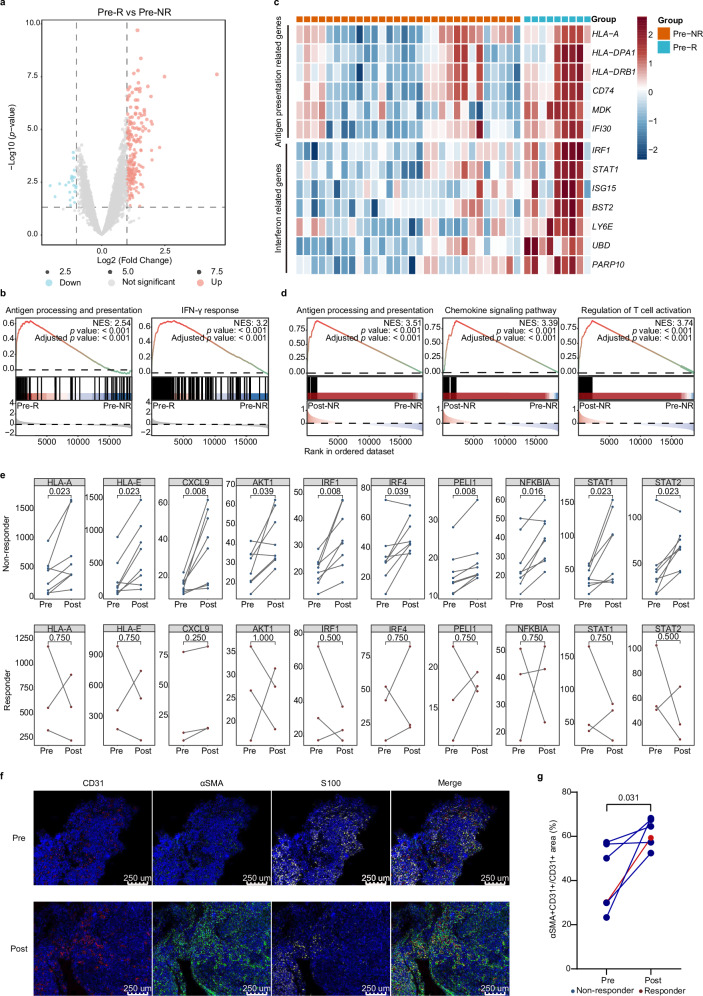
Tumor-intrinsic features and vascular remodeling before and after neoadjuvant treatment. **a** volcano plot of differentially expressed genes (DEGs) beween regions of interest (ROIs) from responders (*n* = 9) and non-responders (*n* = 30) at baseline in the melanoma cell compartment. Horizontal and vertical dashed lines indicate the significance thresholds of ${\mbox{|}}{\log }_{2}{\mbox{fold change}}{\mbox{|}}\ge 1$ and adjusted *P* < 0.05, respectively. *P* values were calculated using moderated t-test implemented in limma. **b** GSEA plots showing enrichment of antigen processing and presentation and interferon-γ signaling in baseline responder tumors. NES: normalized enrichment score. *P* values were calculated using a permutation test (1000 permutations), and adjusted *P* values were obtained using the Benjamini–Hochberg method. **c** Heatmap of representative antigen processing and presentation and interferon-related genes at baseline. **d** GSEA plots for representative gene sets enriched in ROIs from non-responders post-treatment (*n* = 38) vs. pre-treatment (*n* = 30). *P* values were calculated using a permutation test (1000 permutations), and adjusted *P* values were obtained using the Benjamini–Hochberg method. **e** Expression of representative immune-related genes in paired non-responders (*n* = 8) and responders (*n* = 3) pre- and post-treatment. The mean value was calculated by averaging multiple ROIs from the same specimen. Lines connect paired samples. Statistical significance was assessed by two-sided Wilcoxon tests. **f** Representative mIHC images of CD31 (endothelial cells), αSMA (pericytes), and S100 (melanoma cells) on paired tumor samples before and after neoadjuvant therapy. **g** Quantification of vascular normalization level, defined as the proportion of CD31⁺αSMA⁺ vessels among CD31⁺ vessels, in tumors pre- and post-neoadjuvant (*n* = 6). *P* values were determined by two-sided Wilcoxon test. Source data are provided as a Source Data file.

To explore tumor-intrinsic features linked to treatment response, transcriptional data from S100/Pmel^+^ tumor ROIs were analyzed across groups. Differentially expressed genes between responders and non-responders at baseline are summarized in the volcano plot (Fig. 4a). At baseline, GSEA indicated enrichment of antigen processing and presentation (NES = 2.54, adjusted *p* < 0.001) and IFN-γ response signaling (NES = 3.2, adjusted *p* < 0.001) in responders (Fig. 4b), consistent with higher expression of immune-related transcripts (*HLA-A*, *HLA-DPA1*, *HLA-PRB1*, *CD74*, *MDK*, *IFI30*, *IRF1*, *STAT1*, *ISG15*, *BST2*, *LY64*, *UBD*, *PARP10*) in the corresponding heatmap (Fig. 4c). However, within non-responders, post-treatment tumor ROIs showed enrichment of antigen processing and presentation (NES = 3.51, adjusted *p* < 0.001), chemokine signaling (NES = 3.39, adjusted *p* < 0.001), and T-cell activation pathways (NES = 3.74, adjusted *p* < 0.001) compared with pre-treatment (Fig. 4d). Concordantly, key antigen presentation molecules and components of the IFN and NF-κB pathways, which are central to chemokine production and immune cell recruitment, were upregulated in non-responders but remained largely unchanged in responders (Fig. 4e). These findings indicate that pembrolizumab plus lenvatinib enhances tumor immunogenicity in non-responders.

Lenvatinib has been reported to normalize tumor vasculature, thereby improving the efficacy of chemotherapy and ICI^16,17^. We next assessed treatment-induced vascular remodeling using mIHC. Paired pre- and post-treatment tumor samples from six patients (one pPR and five pNR) were stained for CD31 (endothelial cells), αSMA (pericytes), and S100 (melanoma cells). CD31⁺αSMA⁺ double-positive vessels were defined as normalized vasculature. Post-treatment samples from all six patients demonstrated an increased proportion of normalized vessels (*p* = 0.031, Fig. 4f, g), indicating improved vascular architecture following combination therapy. Collectively, these results suggest that neoadjuvant pembrolizumab plus lenvatinib induces tumor-intrinsic immunogenic remodeling, facilitates normalization of the tumor vasculature, and increases infiltration of immune cells in initially immune-cold tumors.

### Divergent intratumoral TCR repertoire dynamics in responders and non-responders

**Fig. 5:**
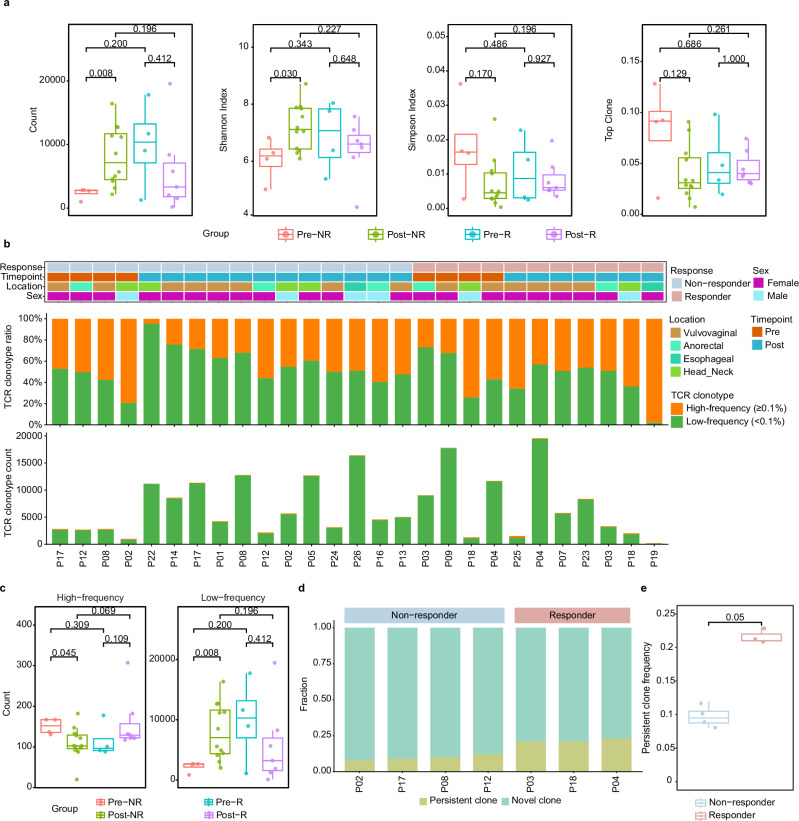
Intratumoral TCR repertoire dynamics during neoadjuvant treatment. **a** TCR sequencing was performed on non-responders pre-treatment (pre-NR, *n* = 4) and post-treatment (post-NR, *n* = 12), and responders pre-treatment (pre-R, *n* = 4) and post-treatment (post-R, *n* = 7). Clonotype count, Shannon diversity, Simpson index, and top clonotype contribution across response groups and time points. **b** Intratumoral clonotypes were classified by frequency into high-frequency (≥0.1%) and low-frequency (<0.1%) groups; the corresponding proportions and counts are shown. **c** Group-wise comparisons of high- and low-frequency clonotypes. **d** In paired tumors (*n* = 7), the fractions of persistent clonotypes shared between pre- and post-treatment samples and novel clonotypes detected only post-treatment are shown. **e** Comparison of persistent clonotype frequency between responders (*n* = 3) and non-responders in the paired cohort (*n* = 4). For box plots, the center line represents the median, box limits represent lower and upper quartiles, and whiskers extend to minimum and maximum values; individual data points are overlaid. All statistical significance was determined by two-sided Mann–Whitney U tests. Source data are provided as a Source Data file.

TCR sequencing was performed on 8 pre-treatment biopsies (responders, *n* = 4; non-responders, *n* = 4) and 19 post-treatment surgical specimens (responders, *n* = 7; non-responders, *n* = 12) from 20 patients (responders, *n* = 8; non-responders, *n* = 12), including 7 paired pre- and post-treatment cases (responders, *n* = 3; non-responders, *n* = 4). In non-responders, clonotype count (*p* = 0.008) and Shannon diversity (*p* = 0.030) increased after therapy, whereas the Simpson index (*p* = 0.170) and the contribution of top clonotypes (*p* = 0.129) decreased, indicating repertoire broadening (Fig. 5a). To further characterize clonal remodeling, we classified intratumoral clonotypes as high-frequency (≥0.1%) or low-frequency (<0.1%) based on abundance (Fig. 5b). Non-responders showed a significant increase in low-frequency clonotypes (*p* = 0.008) accompanied by a decrease in high-frequency clonotypes (*p* = 0.045), whereas responders showed the opposite pattern (Fig. 5c), resulting in a higher post-treatment representation of high-frequency clonotypes in responders than in non-responders (*p* = 0.070). In responders, high-frequency clonotypes increased (*p* = 0.109) while Shannon (*p* = 0.648) and Simpson (*p* = 0.927) index showed limited change, suggesting a more focused clonal expansion. In paired cases, the fraction of persistent clonotypes shared between pre- and post-treatment specimens was higher in responders than in non-responders (*p* = 0.05, Fig. 5d, e). Together, these data indicate distinct response-associated patterns of TCR repertoire remodeling, with responders showing preferential reinforcement of expanded and persistent clonotypes and non-responders showing repertoire broadening dominated by low-frequency clonotypes.

## Discussion

This study proides both the clinical efficacy and underlying biological mechanisms of peri-operative pembrolizumab plus lenvatinib in MM. Our study revealed a pCR rate of 9.5% and pathological response rate of 38.1% in the surgical population, with a median RFS was 14.8 months. Data from prospective studies investigating neoadjuvant or adjuvant approaches for MM remain sparse. In the S1801 study, neoadjuvant-adjuvant pembrolizumab improved outcomes in resectable melanoma with a pCR rate of 38%, but only four MM patients were included and efficacy data for this subgroup were not provided^12^. By contrast, NADINA showed a high pCR rate of 48% and reduced recurrence risk with neoadjuvant nivolumab plus ipilimumab in stage III melanoma, but MM was not enrolled^13^. A separate neoadjuvant trial testing the oncolytic virus (Orienx010) plus toripalimab in acral melanoma reported a pCR rate of 14.8% and a two-year RFS rate of 81.5%^25^.

In our cohort, patients achieving a pathological response tended to have longer median RFS than those without response (15.9 vs. 12.8 months), although this difference was not statistically significant. This pattern is in line with the only other neoadjuvant study in MM to date combined toripalimab with axitinib, which reported a PRR rate of 33.3% and mRFS of 9.5 months, with responders showing a numerically longer RFS than non-responders (11.7 vs. 6.2 months; *p* = 0.45)^26^. Taken together, available studies suggest that neoadjuvant immunotherapy regimens generally yield lower PRR in MM than in CM, and the lack of a statistically significant RFS separation among pathological responders may reflect the propensity of MM for early systemic dissemination^27–29^, together with limited sample size and event numbers in current studies. Larger cohorts with longer follow-up will be required to define the prognostic value of pathological response in MM and whether it translates into durable benefit comparable to that seen in CM.

In the adjuvant setting, a chemotherapy regimen achieved a mRFS of 20.8 months^30^, while anti-PD-1 monotherapy yielded a mRFS of 13.6 months^31^. Although the addition of lenvatinib in the neoadjuvant setting did not result in dramatic gains over adjuvant anti-PD-1 therapy alone, our results suggest some improvement, particularly in the tumor microenvironment. The majority of recurrences observed in our cohort were distant metastases, although local recurrence predominated in patients with vulvovaginal melanoma. This pattern may be influenced by anatomical constraints that limit the extent of surgical resection in the vulvovaginal region. In terms of safety, most TRAES were low grade. Grade 3 events occurred in only three individuals, and no grade 4 or 5 toxicities occurred. These results are consistent with safety profiles reported in other trials using pembrolizumab and lenvatinib combinations^32,33^.

Our omics analyses provided key mechanistic insights into features associated with response and TIME dynamics following neoadjuvant pembrolizumab plus lenvatinib in MM. At baseline, responders showed a pre-existing immune-active TIME, with higher infiltration of activated CD4⁺ and CD8⁺ T cells, and tumor cells displaying higher immune-related programs than non-responders. After treatment, DSP and mIHC further supported increased activated T cell signals in responders, including higher activated CD4⁺/CD8⁺ signatures and cell densities, together with shorter nearest-neighbor distances among activated CD4⁺ T cells, activated CD8⁺ T cells, and melanoma cells, suggesting enhanced immune–tumor engagement. Pathway analyses in responders also highlighted upregulation of immune-related programs, including T-cell receptor signaling, Toll-like receptor signaling, B-cell receptor signaling, and antigen processing and presentation. In addition, responders showed an increased representation of high-frequency clonotypes with limited changes in Shannon and Simpson indicators, indicating a more focused clonal expansion. Collectively, in our cohort, pathological response was associated with an inflamed baseline TIME, coordinated increases in activated T-cell signals and immune–tumor proximity, and preferential reinforcement of expanded and persistent clonotypes.

By contrast, baseline biopsies from non-responders showed lower infiltration of activated CD8⁺ and CD4⁺ T cells, suggesting a “cold” immune phenotype. Following treatment, immune infiltration increased in non-responders, including activated T cell subsets, partially narrowing baseline differences between response groups. At the tumor-intrinsic level, pembrolizumab and lenvatinib treatment enhanced the expression of genes involved in antigen presentation, interferon signaling, and chemokine production in non-responders. This finding implies that immune activation can be induced even in non-inflamed tumors. However, repertoire richness and Shannon diversity increased post-treatment while high-frequency clonotypes decreased, suggesting broader repertoire remodeling dominated by low-frequency clonotypes; this pattern may reflect bystander recruitment or ineffective antigen-driven expansion. Current neoadjuvant protocols in CM commonly use 6–8 weeks of therapy^32^. In our cohort, the post-treatment immune activation observed in some tumors with cold baseline TIME raises the question of whether a longer neoadjuvant course, and/or combination with adoptive T cell approaches (e.g., TIL therapy or engineered TCR therapies), could further enhance immune remodeling in selected patients with MM. These possibilities will need to be tested in future prospective studies.

Higher infiltration of activated T cells after neoadjuvant therapy was significantly correalted with prolonged RFS and DMFS. These associations were further validated using an external bulk RNA-seq cohort of patients with advanced MM receiving first-line atezolizumab plus bevacizumab, where higher activated CD4⁺/CD8⁺ T cell signals similarly correlated with favorable outcomes. Across both datasets, T cell–inflamed states were consistently linked to better clinical outcomes under ICI plus anti-angiogenic therapy. This concept is further supported by the vascular remodeling observed by mIHC: lenvatinib increased the proportion of CD31⁺αSMA⁺ vessels, consistent with vascular normalization that may facilitate immune-cell trafficking and augment the effects of pembrolizumab.

This study has several limitations. First, it is a single-arm design and relatively small sample size limit statistical power and the strength of the conclusions. Accordingly, the results should be considered exploratory and interpreted cautiously. Second, ROI placement prioritized intratumoral regions, resulting in sparse sampling of tumor-adjacent stroma and no dedicated evaluation of the tumor–stroma interface; future studies with more balanced compartment sampling will be needed to better characterize localization-dependent immune features in MM. Third, bulk TRB repertoire profiling does not allow inference of specific TCR–neoantigen pairings; therefore, the response-associated TCR remodeling patterns observed here should be further examined in larger MM cohorts using single-cell TCR sequencing paired with scRNA-seq. Finally, whether activated CD4⁺/CD8⁺ T cell signatures have predictive utility beyond prognostic association will require validation in biomarker-stratified clinical trials.

In conclusion, peri-operative pembrolizumab combined with lenvatinib was generally well tolerated and exhibited encouraging efficacy in MM, promoting tumor immunogenicity, vascular normalization, and enhanced TIME composition, including in tumors with relatively cold baseline immune landscapes. Activated CD4⁺ and CD8⁺ T cells were associated with favorable survial outcomes, and clinical benefit was characterized by preferential reinforcement of expanded and persistent clonotypes rather than indiscriminate repertoire broadening. Our findings provide a rational for ongoing efforts to optimize peri-operative therapy strategies in mucosal melanoma, a disease with historically limited treatment options and poor prognosis.

## Methods

### Study design and participants

The study was approved by the Peking University Cancer Hospital and Research Institute’s ethics committee, and conducted at the Melanoma and Sarcoma Department. All participants provided written informed consent. We confirm that the study design and all clinical procedures complied with all relevant ethical regulations regarding the use of human study participants. The trial was conducted in compliance with the Declaration of Helsinki. This study was a single-arm, open-label, single-center phase II trial evaluating the safety and efficacy of neoadjuvant pembrolizumab plus lenvatinib followed by adjuvant pembrolizumab monotherapy in patients with resectable MM. The trial was registered on ClinicalTrials.gov on November 9, 2020 (NCT04622566, https://clinicaltrials.gov/study/NCT04622566?term=NCT04622566&viewType=Card&rank=1). The study was designed as an exploratory, estimation-focused phase II trial rather than a confirmatory single-arm trial with a prespecified null pCR benchmark. Based on previously reported ORRs of 13–23% in metastatic MM from the KEYNOTE-151 trial, we hypothesized a target pCR rate of 30% for this neoadjuvant combination therapy. The sample size was determined using a precision-based strategy, aiming for a 95% CI width of ~40% around the pCR estimate. Allowing for a 10% dropout rate, we targeted an enrollment of 26 patients, consistent with the exploratory nature of the study and the rarity of MM. As a post hoc sensitivity analysis, an exact one-sample binomial test (one-sided α = 0.05) confirmed that a sample size of 25 would provide 80% power to detect a pCR rate of 30% against a null hypothesis of 10%. Patients eligible for this study were between 18 and 75 years old, had histologically confirmed resectable MM, an ECOG score of 0 or 1, and sufficient hepatic, renal, and hematologic function. Key exclusion criteria comprised prior systemic anti-cancer therapy, active autoimmune disease, chronic infections, or immunodeficiency. The study protocol and the integrated statistical analysis plan are provided in the Supplementary Information (Supplementary Note 1).

### Treatment protocol and clinical assessments

The exact dates of first and last patient enrolment were September 26, 2021 and April 4, 2023. Participants were administered lenvatinib at a daily oral dose of 20 mg alongside pembrolizumab given intravenously at 200 mg every three weeks for two treatment cycles over a 6-week neoadjuvant treatment period. Patients with resectable disease proceeded to definitive surgery within 1–4 weeks of completing neoadjuvant treatment. Following surgery, eligible patients initiated adjuvant pembrolizumab monotherapy (200 mg IV every three weeks) within 6 weeks, for up to 15 additional cycles. Sex was determined based on medical records, and no sex-based subgroup analysis was performed.

The primary endpoint of the study was pCR rate. The secondary endpoint of the study were 1-year RFS rate, OS, clinical response, major pathological response rate, surgical outcomes, and safety. The pathologic response rate was defined as the proportion of patients achieving pCR, near-pCR, or pPR in the resected specimen. Pathologic response rate was assessed in both the surgical cohort and the ITT population, with patients who did not undergo resection considered as non-responders for the primary endpoint analysis. Surgical specimens were centrally reviewed to assess pathological response. The pCR was defined as the absence of viable tumor cells in the resected specimen. Near-pCR was defined as ≤10% viable tumor cells, pPR as 11%–50% viable tumor cells, and pNR as >50% viable tumor cells in the tumor bed^34^. MPR was defined as ≤10% residual viable tumor cells in the resection specimen, comprising both pCR and near-pCR. EFS was defined as the time from treatment initiation to an event to the first occurrence of disease progression precluding surgery, recurrence after surgery, or death. All the secondary endpoints and biomarker analyses were pre-specified as exploratory rather than confirmatory. Postoperative surveillance included imaging every 12 weeks during the first year, every four months in the second year, and every six months from the third to fifth years, or until disease recurrence, death, or withdrawal from the study. Surgical outcomes will be reported separately and are not detailed in this manuscript. Safety was evaluated in all patients who received at least one dose of pembrolizumab or lenvatinib. Adverse events were continuously monitored and classified based on the National Cancer Institute’s Common Terminology Criteria for Adverse Events (CTCAE), version 5.0.

### WES data processing

Genomic DNA was extracted from FFPE tumor specimens and matched peripheral blood leukocytes, fragmented to ~200–300 bp, and quantified for library preparation. Libraries were constructed using a custom 53 Mb capture probe set (IDT, IA, USA) and sequenced as 100-bp paired-end reads on the DNBSEQ-T7RS platform (BGI, China). Reads were filtered to remove adaptor sequences and low-quality reads and aligned to the human reference genome (hg19) using BWA (v0.7.10). Somatic variants were identified from tumor–normal comparisons and restricted to non-synonymous coding variants. Variants with fewer than five high-quality reads in tumor samples (MAPQ ≥ 30; base quality ≥30) or with a variant allele frequency <3% were filtered out, and matched blood was used to exclude germline events. Copy-number profiles were inferred using CNVkit from normalized tumor-to-normal coverage ratios, with log2 copy-ratio ≥0.848 or ≤−0.737 classified as amplifications or deletions, respectively; tumor–normal pairs with excessive coverage variability were excluded during QC, and recurrent copy-number events were summarized using GISTIC2.0 (q < 0.1). TMB was calculated as the number of somatic, non-synonymous coding mutations with variant allele frequency ≥3% divided by the callable territory covered by the capture design (33 Mb). For neoantigen analysis, four-digit HLA class I genotypes were inferred from matched normal WES data using OptiType; candidate 8–11-mer mutant peptides were generated from non-silent mutations and evaluated with netMHCpan (v3.0), and putative neoantigens were defined by predicted binding affinity <500 nM and mutant-to-wild-type binding affinity ratio <0.7, with TNB reported as neoantigens per megabase. HLA LOH was assessed using HLALOH with HLA genotypes supported by OptiType and/or POLYSOLVER, and MSI status was evaluated using MSIsensor (v0.2).

### GeoMx digital spatial profiling

Four-micrometer sections from FFPE samples were deparaffinized and underwent antigen retrieval in Tris-EDTA buffer. RNA-binding proteins were removed using the GeoMx DSP RNA Slide Prep Kit (NanoString, USA), followed by fixation with 16% formaldehyde for five minutes at room temperature. Probes targeting 18,000 RNA molecules (GeoMx Panel and Seq Code kit, NanoString) were hybridized overnight. These probes, linked to UV-photocleavable DNA oligos containing unique molecular identifiers (UMIs), enabled precise spatial profiling. Slides were further stained with morphological markers S100 (NBP2-54426, Novus, 1:400) and Pmel17 (NBP2-34638, Novus, 1:400), CD45 (13917, Cell Signaling, 1:100) and Syto 13 (S7575, Thermo, 1:1000) for nuclear identification, followed by incubation with fluorescent secondary antibodies. Scanned slides were processed in a GeoMX Digital Spatial Profiler (NanoString) to select ROIs. ROIs were selected on scanned sections using histology and immunofluorescence signals and were restricted to on-tissue regions within pathologist-annotated tumor and stroma compartments, with a preference for intratumoral regions. DNA oligos in the ROIs were collected and prepared for subsequent sequencing using an Illumina NovaSeq 6000 platform.

Raw digital count conversion (DCC) files were normalized using ERCC RNA spike-in controls to mitigate system bias and adjust for ROI size variation. ROIs were included if they met criteria of a minimum surface area of 1.6 × 10⁴ μm² and at least 200 nuclei. ROIs with normalization positive factors outside 0.3–3.0 were excluded. Normalized data were processed using Q3 (third quartile) normalization, log-transformation, and optional median centering. Data processing and analysis were performed using DSP analysis software and R version 4.5.1. Significantly differentially expressed genes between groups were performed using the limma software, and the criteria of |log2 fold change | ≥ 1 and FDR < 0.05.

For functional interpretation, DEGs were subjected to GO/KEGG enrichment analysis using Metascape (enrichment *p* < 0.01), and pathway-level enrichment was additionally assessed by GSEA using the R package clusterProfiler with MSigDB gene sets, applying an adjusted *p*-value < 0.05. Immune phenotypes were derived from curated gene expression signatures using ssGSEA within CD45^+^ (immune compartment) ROIs, generating per-ROI immune cell signature scores. For heatmaps visualization, gene expression values were z-score standardized across samples. Volcano plots were generated using cutoffs of |log2 fold change | ≥ 1 and adjusted p-value < 0.05 (dashed lines). For paired analyses, ROI-level values were first averaged within each specimen to generate one value per sample.

### Multiplex immunohistochemistry

For immune phenotyping, FFPE sections (4 μm) underwent standard deparaffinization/rehydration and heat-induced antigen retrieval (EDTA buffer, pH 9.0; Panovue), followed by iterative TSA-based multiplex staining for GZMB (CST46890, Cell Signaling, 1:200), CD4 (ZA-0418, Zsbio, 1:200), Ki-67 (CST9027, Cell Signaling, 1:300), ICOS (CST89601S, Cell Signaling, 1:150), SOX10 (ZA-0624, Zsbio, ready-to-use), and CD8A (CST70306, Cell Signaling, 1:200) (Supplementary Table 2). Primary antibodies were applied in a pre-optimized sequence with HRP-conjugated secondary antibodies and TSA amplification, with heat-mediated stripping between cycles, and nuclei were counterstained with DAPI. Whole-slide images were acquired on an Olympus VS200 scanner. Tumor and stromal areas were reviewed by a pathologist and annotated to exclude regions unsuitable for analysis. Single-cell segmentation and marker-positivity calling were performed in QuPath (v0.5.1) using channel-specific intensity thresholds. Cell frequency and density were quantified from phenotype-positive and total cell counts and the corresponding analyzed area, and nearest-neighbor distances were calculated using Euclidean distance based on 2D cell coordinates exported from QuPath.

For vascular maturation, FFPE sections were stained for CD31, αSMA, and S100 using an Opal multiplex workflow (Akoya). After deparaffinized and rehydrated, antigen retrieval was carried out using AR6 buffer (Akoya Biosciences) in a microwave. Samples were treated with 3% hydrogen peroxide for 10 minutes to suppress endogenous peroxidase activity. Multiplex staining was performed through sequential cycles, each involving blocking with 1% BSA, primary antibody incubation, and succeeded by incubation with HRP-conjugated secondary antibody, and visualization using Opal fluorophores (Akoya). After each cycle, antibodies were stripped via heat-induced retrieval to allow the next round of staining. The antibody/fluorophore pairs used were CD31/Opal 620 (ab182981, Abcam, 1:2000), S100/Opal 570 (ab52642, Abcam, 1:1000), and αSMA/Opal 520 (ab124964, Abcam, 1:300) (Supplementary Table 2). Slides underwent counterstaining with spectral DAPI and were then mounted using antifade medium (Abcam). Images were captured with the Pannoramic Scan II Digital Scanncer (3DHISTECH).

### TCR sequencing

Immunosequencing of the CDR3 region of human TCRβ chains was performed using a multiplex PCR approach with a V- and J-targeted primer set designed to capture diverse V(D)J rearrangements^35^, followed by a second-round amplification with universal primers for library construction. Libraries were sequenced on the DNBSEQ-T7RS (BGI, China). CDR3 was defined according to IMGT as the amino acid sequence between the conserved cysteine in the V region and the conserved phenylalanine in the J region. V(D)J assignment and CDR3 extraction were performed using MiXCR. TCR repertoire diversity was summarized using Shannon and Simpson indices based on productive clonotypes; clonotype richness was defined as the number of unique productive clonotypes, and top clone abundance was defined as the frequency of the most abundant clonotype.

### Statistical analysis

Response rates were calculated as proportions and reported with two-sided 95% exact confidence intervals using the Clopper–Pearson method. For continuous variables, group comparisons between independent cohorts were conducted using the unpaired Student’s t-test or the Mann–Whitney U test, as appropriate. For paired pre- and post-treatment samples, the Wilcoxon rank-sum test was applied. Categorical variables were compared using the Fisher’s exact test. Time-to-event endpoints (RFS, EFS, DMFS, and OS) were analyzed using the Kaplan–Meier method and compared via the log-rank test in R with Survminer and Survival packages. Multivariable Cox proportional hazards regression models were used to assess the association between candidate biomarkers and time-to-event endpoints, adjusting for relevant clinical covariates. All statistical analyses were performed using R (version 4.5.1), with a significance threshold of *α* = 0.05. A *p*-value of less than 0.05 (two-sided) was considered statistically significant.

### Reporting summary

Further information on research design is available in the Nature Portfolio Reporting Summary linked to this article.

## Supplementary information

Supplementary Information

Reporting Summary

Transparent Peer Review file

## Source data

Source data

## Data Availability

All data supporting the findings of this study are available within the Article, Supplementary Information, and the Source Data files. De-identified patient data are provided in the Source Data. The WES data generated in this study have been deposited in the Genome Sequence Archive under accession code HRA015548. The GeoMx DSP data generated in this study are deposited in the Genome Sequence Archive under accession code HRA015543. The TCR sequencing data generated in this study are deposited in the Genome Sequence Archive under accession code HRA015547. The bulk RNA sequencing data of the validation cohort are deposited in the Genome Sequence Archive under accession code HRA008154. Qualified researchers may request access to the raw sequencing data to protect patient privacy. Requests should be directed to the corresponding author and will be formally reviewed by the institutional ethics committee based on the scientific merit of the proposal and the applicant’s institutional affiliation. Upon approval and the signing of a Data Transfer Agreement (DTA), data access will be granted for a period of 12 months. All requests will be processed within 3 weeks. The study protocol is available in the Supplementary Information file. Source data are provided with this paper.

## Code availability

The custom code used to generate the results and figures in this study is available without restriction at (https://github.com/JieDai-PKU/P2-PLMM^36^). Detailed instructions are provided in the README file on the repository. Any other standard software packages and algorithms used are cited in the Methods section.
