# Supplementary material for: A phase II peri-operative study of pembrolizumab plus lenvatinib for mucosal melanoma
Source: Nat Commun. 2026 May 16;17:6491. doi: 10.1038/s41467-026-73190-1 (PMC13376369; doi:10.1038/s41467-026-73190-1)
Supplement: Supplementary file 1 — Supplementary Information [file 41467_2026_73190_MOESM1_ESM.pdf]

Supplementary Information

A phase II peri-operative study of pembrolizumab plus lenvatinib for mucosal melanoma

Lili Mao[1†], Yumei Lai[2†], Hong Zheng[3†], Ming Cui[4†], Lifeng Li[5], Ziyi Liu[1], Hongyu Zhou[1], Linzi Sun[1], Caili Li[1], Xiaoting Wei[1], Junjie Gu[6], Xue Bai[1], Yan Kong[1], Chuanliang Cui[1], Zhihong Chi[1], Xinan Sheng[6], Bin Lian[1], Siming Li[1], Xieqiao Yan[6], Bixia Tang[6], Juan Li[6], Li Zhou[1], Xuan Wang[1], Jun Guo[1,6], Jie Dai[1*], Lu Si[1*]

[1] Key Laboratory of Carcinogenesis and Translational Research (Ministry of Education/Beijing), Department of Melanoma and Sarcoma, Peking University Cancer Hospital and Institute, Beijing 100142, China.

[2] Key Laboratory of Carcinogenesis and Translational Research (Ministry of Education/Beijing), Department of Pathology, Peking University Cancer Hospital and Institute, Beijing 100142, China.

[3] Key Laboratory of Carcinogenesis and Translational Research (Ministry of Education/Beijing), Department of Gynaecology, Peking University Cancer Hospital and Institute, Beijing 100142, China.

[4] Key Laboratory of Carcinogenesis and Translational Research (Ministry of Education/Beijing), Department of Gastrointestinal Surgery IV, Peking University Cancer Hospital and Institute, Beijing 100142, China.

[5] Geneplus-Beijing, Beijing 102206, China.

[6] Key Laboratory of Carcinogenesis and Translational Research (Ministry of Education/Beijing), Department of Genitourinary Oncology, Peking University Cancer Hospital and Institute, Beijing 100142, China.

[*]These authors jointly supervised this work.

[†]These authors contributed equally to this work.

Supplemental Table 1. Patient characteristics and samples for omics analysis

Patient ID	Location	Surgery	Response group	WES		DSP		mIHC-immune		TCR		mIHC-vascular	
				Pre	Post	Pre	Post	Pre	Post	Pre	Post	Pre	Post
P01	Vulvovaginal	Yes	Non-responder	No	Yes	No	No	No	Yes	No	Yes	No	No
P02	Head & Neck	Yes	Non-responder	Yes	Yes	Yes	Yes	No	Yes	Yes	Yes	Yes	Yes
P03	Anorectal	Yes	Responder	Yes	Yes	Yes	Yes	No	Yes	Yes	Yes	No	No
P04	Vulvovaginal	Yes	Responder	Yes	Yes	Yes	Yes	No	Yes	Yes	Yes	No	No
P05	Head & Neck	Yes	Non-responder	No	Yes	No	Yes	No	No	No	Yes	No	No
P06	Anorectal	No	NA	No	No	No	No	No	No	No	No	No	No
P07	Vulvovaginal	Yes	Responder	No	Yes	No	Yes	No	Yes	No	Yes	No	No
P08	Vulvovaginal	Yes	Non-responder	Yes	Yes	Yes	Yes	No	Yes	Yes	Yes	No	No
P09	Vulvovaginal	Yes	Responder	Yes	No	Yes	Yes	Yes	Yes	Yes	No	Yes	Yes
P10	Anorectal	No	NA	No	No	No	No	No	No	No	No	No	No
P11	Anorectal	No	NA	No	No	No	No	No	No	No	No	No	No
P12	Anorectal	Yes	Non-responder	Yes	Yes	Yes	Yes	Yes	Yes	Yes	Yes	Yes	Yes
P13	Vulvovaginal	Yes	Non-responder	No	Yes	No	No	No	Yes	No	Yes	No	No
P14	Vulvovaginal	Yes	Non-responder	Yes	Yes	Yes	Yes	Yes	Yes	No	Yes	No	No
P15	Head & Neck	No	NA	No	No	No	No	No	No	No	No	No	No
P16	Anorectal	Yes	Non-responder	No	Yes	Yes	Yes	Yes	Yes	No	Yes	Yes	Yes
P17	Vulvovaginal	Yes	Non-responder	Yes	Yes	Yes	Yes	Yes	Yes	Yes	Yes	No	No
P18	Head & Neck	Yes	Responder	Yes	Yes	No	Yes	Yes	Yes	Yes	Yes	No	No
P19	Esophageal	Yes	Responder	No	Yes	Yes	Yes	No	Yes	No	Yes	No	No
P20	Anorectal	Yes	Non-responder	No	No	Yes	Yes	Yes	No	No	No	Yes	Yes
P21	Anorectal	No	NA	No	No	No	No	No	No	No	No	No	No
P22	Head & Neck	Yes	Non-responder	No	Yes	No	No	No	Yes	No	Yes	No	No
P23	Vulvovaginal	Yes	Responder	No	No	No	Yes	No	Yes	No	Yes	No	No
P24	Vulvovaginal	Yes	Non-responder	No	Yes	No	Yes	No	Yes	No	Yes	Yes	Yes
P25	Vulvovaginal	Yes	Responder	No	Yes	No	Yes	No	Yes	No	Yes	No	No
P26	Esophageal	Yes	Non-responder	No	Yes	Yes	Yes	Yes	Yes	No	Yes	No	No

This table summarizes the clinical treatment responses and the availability of multi-omics data for each enrolled patient (n = 26). Pathological responses were evaluated for patients who underwent surgery, while patients who did not receive surgery are indicated as NA (not applicable). For each patient, the presence (Yes) or absence (No) of biospecimens and successful data generation is listed across pre- and post-neoadjuvant treatment time points for the following platforms: WES (whole-exome sequencing), DSP (digital spatial profiling), TCR (T-cell receptor sequencing), and mIHC (multiplex immunohistochemistry).

Supplemental Table 2. Details of antibodies used for multiple immunohistochemistry.

Antibody	Supplier	Catalog	Clone	Lot	Dilution
Granzyme B	Cell signaling	CST46890	D6E9W	6	1:200
CD4	Zsbio	ZM-0418	UMAB64	24050912	1:200
Ki-67	Cell signaling	CST9027	D2H10	10	1:300
ICOS	Cell signaling	CST89601S	D1K2T	11	1:150
SOX10	Zsbio	ZA-0624	EP268	25081752	Ready-to-use
CD8A	Cell signaling	CST70306	C8/144B	4	1:200
CD31	Abcam	ab182981	EPR17259	1075935-61	1:2000
S100	Abcam	ab52642	EP1576Y	1084971-21	1:1000
αSMA	Abcam	ab124964	EPR5368	1092972-41	1:300

Supplementary Figure 1

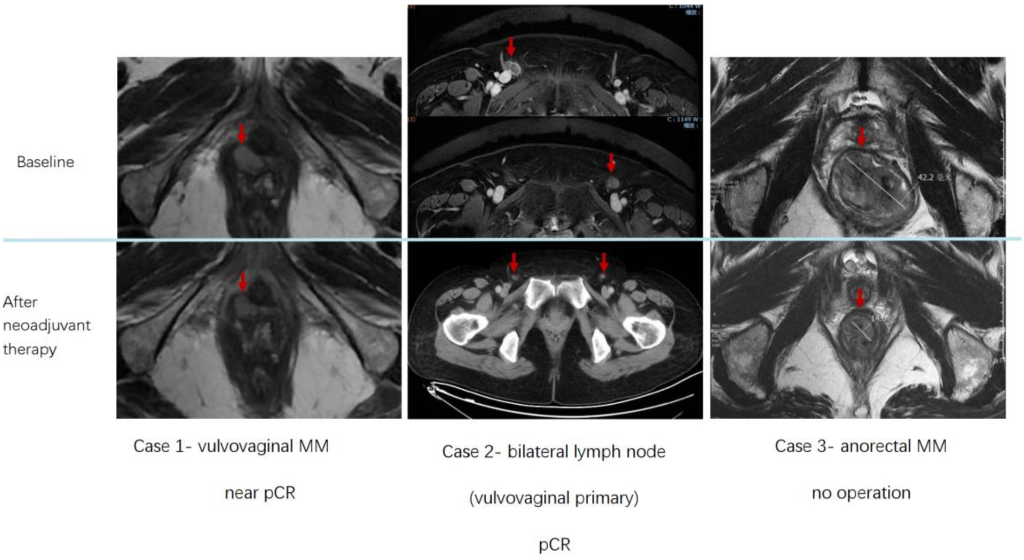

Supplementary Figure 1. Typical images of tumor lesions in patients pre- and post-neoadjuvant therapy. Case 1: Vulvovaginal mucosal melanoma patient, primary tumor shrinked after neoadjuvant therapy, achieved near pCR after surgery. Case 2: Vulvovaginal mucosal melanoma patient, bilateral lymph node shrinked after neoadjuvant therapy, achieved pCR after surgery. Case 3: Anorectal mucosal melanoma patient, primary tumor shrinked after neoadjuvant therapy, the patient declined pre-planned abdominoperineal resection.

Supplementary Figure 2

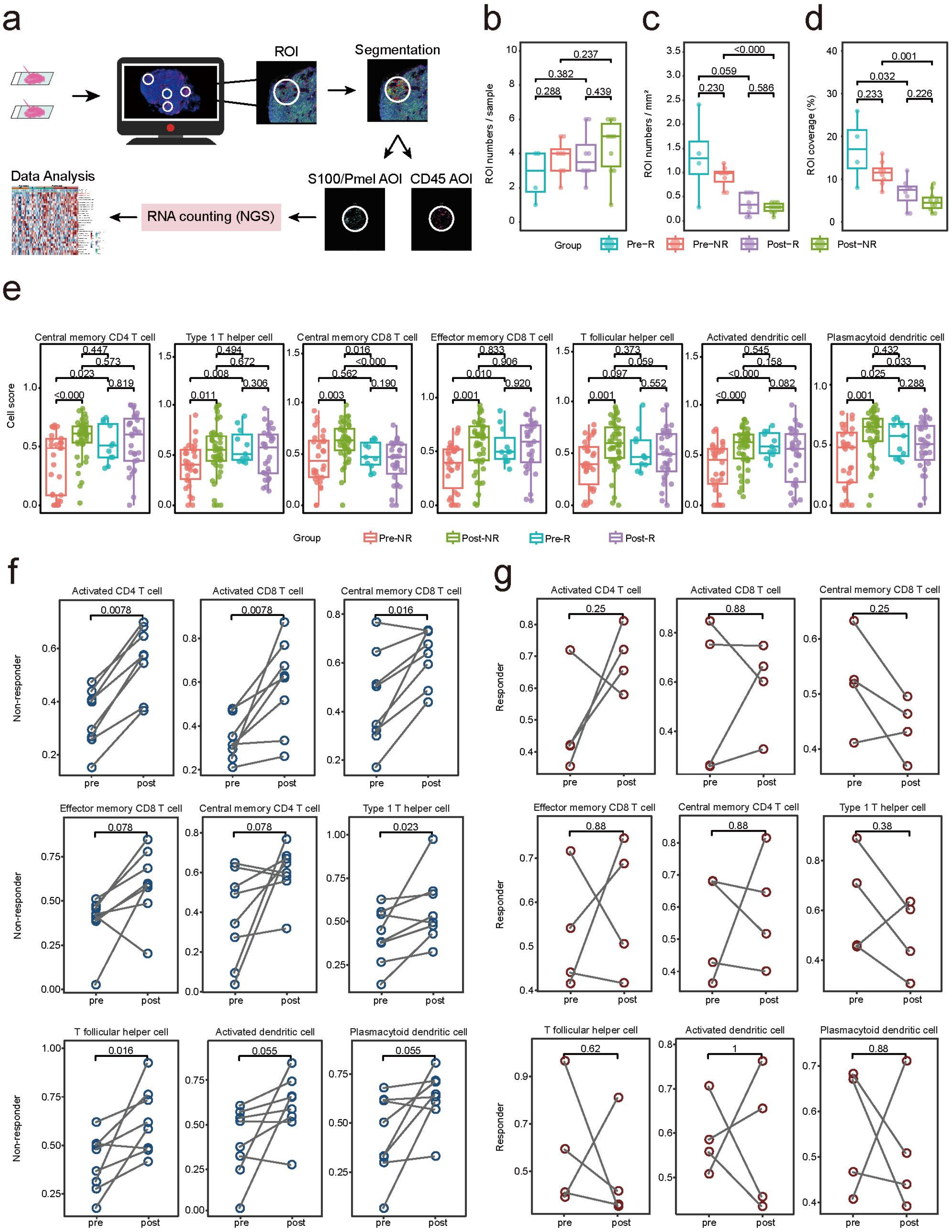

Supplementary Figure 2. DSP workflow and immune cell infiltration level before and after neoadjuvant treatment. a Schematic representation of the DSP workflow and analytical strategy. **b-d** Three indicators were calculated to quantify ROI sampling on the analyzable tissue section (defined as the on-tissue tumor/stroma mask after excluding off-tissue regions and artifacts): (**b**) the number of ROIs per case, (**c**) ROI density (ROIs per

mm^2 analyzable area), and (**d**) ROI coverage (the sum of ROI areas divided by the analyzable tissue area). *P* values were determined by two-sided Mann-Whitney U test. **e** Box plot of immune cell infiltration in ROIs of pre-NR (pre-non-responder, *n* = 30), post-NR (post-non-responder, *n* = 44), pre-R (pre-responser, *n* = 11), and post-R (post-responder, *n* = 31). For box plots (b-e), the center line represents the median, box limits represent lower and upper quartiles, and whiskers extend to minimum and maximum values; individual data points are overlaid. *P* values were determined by unpaired Student's t-test. **f** Immune cell infiltration score in paired non-responders (*n* = 8). **g** Immune cell infiltration score in paired responders (*n* = 4). For paired comparisons in (f, g), the mean signature score was calculated by averaging multiple ROIs from the same specimen. *P* values were determined by two-sided Wilcoxon test. Source data are provided as a Source Data file.

Supplementary Figure 3

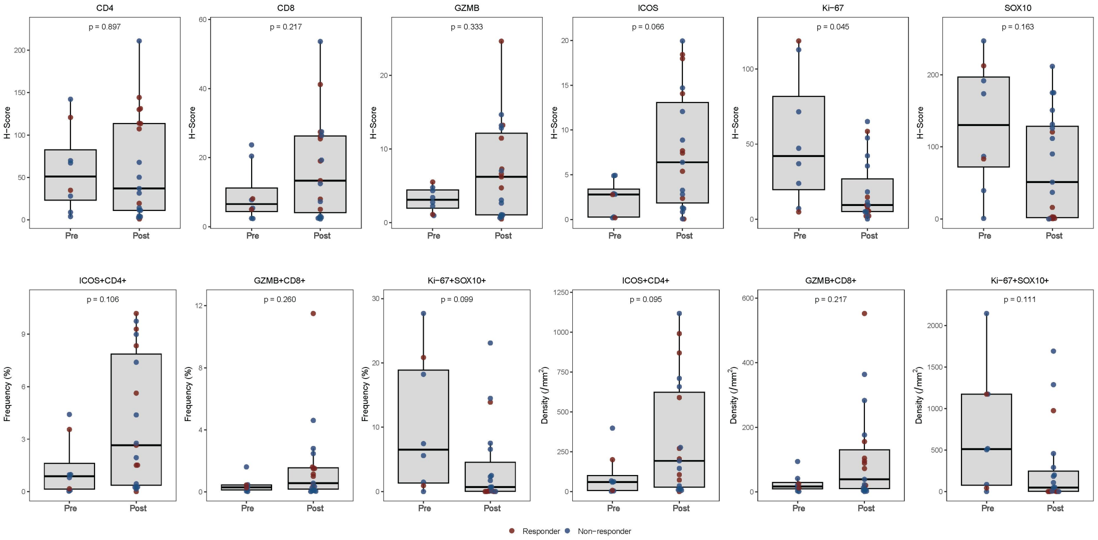

Supplementary Figure 3. Multiplex immunohistochemistry of pre- (*n* = 8) and post-neoadjuvant (*n* = 19) specimens. Box plots show H-scores, positive cell frequencies, and positive cell densities for activated $CD4^+$ T cells ($ICOS^+CD4^+$), activated $CD8^+$ T cells ($GZMB^+CD8^+$), and proliferating melanoma cells ($Ki\text{-}67^+SOX10^+$). For all box plots, the center line represents the median, box limits represent lower and upper quartiles, and whiskers extend to minimum and maximum values; individual data points are overlaid. *P* values were determined by two-sided Mann-Whitney U test. Source data are provided as a Source Data file.

Supplementary Note 1

Clinical Research Protocol

Protocol Title :	A Phase II Study of Neoadjuvant Lenvatinib and Pembrolizumab in Resectable mucosal Melanoma.
Protocol Number：	MISP# 59132
Supporter：	Merck Sharp & Dohme Corp.
Institution:	Peking University Cancer Hospital
Department：	Melanoma & Kidney Cancer
Principal Investigator：	Professor Jun Guo<br>Email: guoj307@126.com
Version Number：	V1.0
Version Date：	04/22/2020

Clinical Study Protocol

PROTOCOL SYNOPSIS

Title	**A Phase II Study of Neoadjuvant Lenvatinib and Pembrolizumab in Resectable mucosal Melanoma.**
Phase	**II**
Study Description	Mucosal melanoma is a rare type of melanoma in Caucasian population and a dominate subtype in Asia, which generally carries a worse prognosis than cutaneous melanoma. Surgery remains the primary therapeutic intervention for mucosal melanoma, and neoadjuvant therapy is still needed which can lead to improvements in outcomes by surgical resectability, local control and organ preservation.<br>Immunotherapy has showed promising activity in advanced melanoma. However, PD-1 antibodies, both Pembrolizumab and Nivolumab, showed much poorer response to mucosal melanoma than to cutaneous melanoma.<br>Another anti-PD-1 antibody JS001(Toripalimab, Shanghai Junshi Biosciences) even did not show any response to mucosal melanoma (ORR 0%, NCT03013101). Surprisingly, JS001 in combination with Axitinib, a selective inhibitor of vascular endothelial growth factor receptors (VEGFR) 1, 2, and 3, had shown remarkable anti-tumor activity (ORR 60.6%) and manageable Adverse Events (Grade 3/4 AE 18%) in advanced mucosal melanoma (NCT03086174).<br>Pembrolizumab showed modest objective response rate (ORR, 13%) in advanced mucosal melanoma in Chinese population (NCT02628067). Recent study also demonstrated that neoadjuvant therapy with one dose of Pembrolizumab could lead to pathological complete response (pCR) or major response in nearly 30% of resectable advanced melanoma and pCR was associated with reduced risk of recurrence and improved survival.<br>Lenvatinib is also a kinase inhibitor that inhibits the kinase activities of VEGFR1,2, 3 which has been approved by FDA for differentiated thyroid cancer, advanced renal cell carcinoma and hepatocellular carcinoma.<br>we hypothesize that neoadjuvant Pembrolizumab in combination with Lenvatinib could result in higher anti-tumor activity with lower toxicity and prolong RFS and OS in high-risk resectable mucosal melanoma.<br>In this study, we will assess the efficacy and safety of the combination Pembrolizumab and Lenvatinib as neoadjuvant treatment in resectable mucosal melanoma.

Objective & Endpoints	**Primary Objective:**<br>To determine the pathological response rate(pCR)<br>**Secondary Objectives**<br>● 1-year relapse-free survival (RFS) rate<br>● Overall survival (OS)<br>● Clinical Response<br>● Major pathologic response rate<br>● Surgical outcomes<br>a.Episodes of infection requiring antibiotics and/or wound drainage.<br>b. Duration of time from surgery end (time to recovery) to removal of wound drain because of ceased / minimal drainage<br>c. Episodes of seroma formation at the wound site requiring any intervention and volume of seroma drainage<br>d. Episodes of bleeding from the wound requiring a return to theatre and / or blood transfusion post-surgery.<br>e. Number of episodes (and patient number) of wound separation requiring any intervention<br>f. Incidence of lymphoedema or worsening lymphedema<br>g. An assessment of 'operability' at the time of surgery compared to the baseline evaluation.<br>h. The time from surgery completion to wound healing<br><br>● **Safety**
**Study Population**	The study will enroll 26 patients who have histologically confirmed resectable mucosal melanoma with sufficient disease to enable,only cases where a complete surgical resection with tumour-free margins can safely be achieved are defined as resectable.<br>Both male and female patients age of 18 years or older who meet the inclusion criteria could be eligible to participate in the study after completing the study enrollment screening test and procudures.
**Description of Sites**	The study will be conducted at Peking University Oncology Hospital, Melanoma & Kidney Center.
**Study Treatment**	Each cycle is defined as 21 days treatment of Pembrolizumab in combination with Lenvatinib.<br>Pembrolizumab 200mg IV day 1 of every 21 day-cycles;<br>Lenvatinib 20mg will be given orally once daily for 21-day cycles<br>After completion of neoadjuvant treatment, Pembrolizumab will be as adjuvant treatment for 15 cycles, 200mg IV day 1 of every 21 day-cycles.
**Study Duration**	24 months

TABLE OF CONTENTS

1.BACKBROUND AND RATIONALE 8
Rationale 9
2.OBJECTIVES AND HYPOTHESES 11
2.1 Primary Objective and hypothesis 11
2.2 Secondary Objectives and hypothesis 11
3. STUDY DESIGN 12
3.1 Overall design 12
3.2 Study Duration 13
3.3 Participant Replacement Strategy 13
3.4 Subject Withdrawal/Discontinuation Criteria 13
3.4.1 Discontinuation of Treatment 13
3.4.2 Withdrawal from the Trial 14
3.5 Eligibility 14
3.5.1 Inclusion criteria 14
3.5.2 Exclusion criteria 15
4.STUDY INTERVENSION 18
4.1 Study Intervention(s) Administered 18
4.2 Dose Modification 18
4.2.1 Pembrolizumab 18
4.2.2 Lenvatinib 21
4.2.3 Dose Modifications for Overlapping Toxicities 22
4.3 Rescue Medications and Supportive Care 23
4.3.1 Supportive Care Guidelines for Pembrolizumab 23
4.3.2 Supportive Care Guidelines for Lenvatinib 27
4.4 Concomitant Therapy 30
4.4.1 Allowed Concomitant Medication(s) 31
4.4.2 Prohibited Concomitant Medication(s) 31
4.4.3 Drug Interactions 32
5. STUDY ASSESSMENTS AND PROCEDURES 32
5.1 Administrative Procedures 33

5.1.1 Informed Consent 33
5.1.1.1 General Informed Consent 33
5.1.1.2 Consent and Collection of Specimens for Future Biomedical Research 33
5.1.2 Inclusion/Exclusion Criteria 33
5.1.3 Medical History 34
5.1.4 Concomitant Medications 34
5.1.4.1 Prior Medications 34
5.1.4.2 Concomitant Medications 34
5.1.5 Study Intervention Administration 34
5.1.5.1 Timing of Dose Administration 34
5.1.5.2 Compliance 35
5.1.6 Discontinuation and Withdrawal 35
5.1.6.1 Discontinuation and Withdrawal from the main study 35
5.1.6.1 Withdrawal From Future Biomedical Research 35
5.1.7 Demography 36
5.2 Clinical Procedures/Assessments 36
5.2.1 Adverse Event Monitoring 36
5.2.2 12-Lead Electrocardiogram 36
5.2.3 Echocardiography or Multigated Acquisition Scan 36
5.2.4 Physical Examination 37
5.2.4.1 Full Physical Examination 37
5.2.5 Vital Signs 37
5.2.6 Eastern Cooperative Oncology Group Performance Status 37
5.2.7 Tumor Tissue Biopsy and Sample Collection 37
5.2.8 Optional Blood Sample Collection for Future Biomedical Research 38
5.2.9 Imaging Disease Assessment 38
5.2.9.1 Initial Tumor Imaging 38
5.2.9.2 Tumor Imaging during the Study 39
5.2.9.3 End-of-Treatment and Follow-up Imaging 39
5.2.10 Endoscopy assessment 39
5.2.11 Definitive surgery 40
5.2.12 Surgical outcomes 40
5.3 Laboratory Procedures/Assessments 40
5.3.1 Laboratory Safety Evaluations (Hematology, Chemistry, and Urinalysis) 41

5.4 Visit Requirements 42
5.4.1 Screening 42
5.4.2 Treatment Cycles 43
5.4.2.1 Neoadjuvant Treatment Phase 43
5.4.2.2 Adjuvant Treatment Phase 43
5.4.3 Definitive Surgery 43
5.4.4 End of treatment Visit 44
5.4.5 Safety Follow-up Visits 44
5.4.6 Long Term Follow-Up for Disease Status and Survival 44
5.4.7 Discontinued Subjects Continuing to be Monitored in the Trial 44
5.4.8 Study Calendar 45
6. Assessing and Recording Adverse Event 48
6.2.1 Time Period and Frequency for Collecting AE, SAE, and Other Reportable Safety Event Information 48
6.2.2 Method of Detecting AEs, SAEs, and Other Reportable Safety Events 50
6.2.3 Follow-up of AE, SAE, and Other Reportable Safety Event Information 50
6.2.4 Sponsor Responsibility for Reporting Adverse Events 50
6.2.5 Pregnancy and Exposure During Breastfeeding 51
6.2.6 Events of Clinical Interest (ECIs) 51
*Note: These criteria are based upon available regulatory guidance documents. The purpose of the criteria is to specify a threshold of abnormal hepatic tests that may require an additional evaluation for an underlying etiology. 51
7. STATISTICAL ANALYSIS PLAN 52
7.1 Variables/Time Points of Interest 52
7.2 Statistical Methods 52
7.3 Power/Sample Size: 52
7.4 Future Collateral Biomarker Research: 53
APPENDICES 54
Appendix 1: ECOG Performance Status 54
Appendix 2: Common Terminology Criteria for Adverse Events V5.0 (CTCAE) 55
Appendix 3: Adverse Events: Definitions and Procedures for Recording, Evaluating, Follow-up, and Reporting 56
Definition of AE 56

Definition of SAE 57
Additional Events Reported in the Same Manner as SAE 58
Recording AE and SAE 59
Reporting of AEs, SAEs, and Other Reportable Safety Events to the MSD 62

1.BACKBROUND AND RATIONALE

Background

Pembrolizumab is a potent humanized immunoglobulin G4 (IgG4) monoclonal antibody (mAb) with high specificity of binding to the programmed cell death 1 (PD 1) receptor, thus inhibiting its interaction with programmed cell death ligand 1 (PD-L1) and programmed cell death ligand 2 (PD-L2). Based on preclinical in vitro data, pembrolizumab has high affinity and potent receptor blocking activity for PD 1. Pembrolizumab has an acceptable preclinical safety profile and is in clinical development as an intravenous (IV) immunotherapy for advanced malignancies. Keytruda® (pembrolizumab) is indicated for the treatment of patients across a number of indications because of its mechanism of action to bind the PD-1 receptor on the T cell. For more details on specific indications refer to the Investigator brochure.

Pharmaceutical and Therapeutic Background

The importance of intact immune surveillance function in controlling outgrowth of neoplastic transformations has been known for decades [Disis, 2010]. Accumulating evidence shows a correlation between tumor-infiltrating lymphocytes in cancer tissue and favorable prognosis in various malignancies. In particular, the presence of CD8+ T-cells and the ratio of CD8+ effector T cells/FoxP3+ regulatory T-cells (T-regs) correlates with improved prognosis and long-term survival in solid malignancies, such as ovarian, colorectal, and pancreatic cancer; hepatocellular carcinoma; malignant melanoma; and renal cell carcinoma. Tumor-infiltrating lymphocytes can be expanded ex vivo and reinfused, inducing durable objective tumor responses in cancers such as melanoma [Dudley et al., 2005; Hunder et al., 2008].

The PD-1 receptor-ligand interaction is a major pathway hijacked by tumors to suppress immune control. The normal function of PD-1, expressed on the cell surface of activated T cells under healthy conditions, is to down-modulate unwanted or excessive immune responses, including autoimmune reactions. PD-1 (encoded by the gene Pdcd1) is an immunoglobulin (Ig) superfamily member related to cluster of differentiation 28 (CD28) and cytotoxic T-lymphocyte-associated protein 4 (CTLA-4) that has been shown to negatively regulate antigen receptor signaling upon engagement of its ligands (PD L1 and/or PD-L2) [Greenwald et al., 2005; Okazaki et al., 2001].

The structure of murine PD-1 has been resolved [Zhang et al., 2004]. PD-1 and its family members are type I transmembrane glycoproteins containing an Ig-variable–type (IgV type) domain responsible for ligand binding and a cytoplasmic tail responsible for the binding of signaling molecules. The cytoplasmic tail of PD-1 contains 2 tyrosine-based signaling motifs, an immunoreceptor tyrosine-based inhibition motif, and an immunoreceptor tyrosine-based switch motif. Following T-cell stimulation, PD-1 recruits the tyrosine phosphatases, SHP-1 and SHP-2, to the immunoreceptor tyrosine-based switch motif within its cytoplasmic tail, leading to the dephosphorylation of effector molecules such as CD3 zeta (CD3ζ), protein kinase C-theta (PKCθ), and zeta-chain-associated protein kinase (ZAP70), which are involved in the CD3 T-cell signaling cascade [Okazaki et al., 2001; Chemnitz et al., 2004; Sheppard et al., 2004; and Riley, 2009]. The mechanism by which PD-1 down-modulates T cell responses is similar to, but distinct from,

that of CTLA-4, because both molecules regulate an overlapping set of signaling proteins [Parry et al., 2005; Francisco, 2010]. As a consequence, the PD 1/PD-L1 pathway is an attractive target for therapeutic intervention in mealnoma.

Rationale

Rationale for the Trial and Selected Population

Mucosal melanoma is a rare type of melanoma in Caucasian population and a dominate subtype in Asia, which generally carries a worse prognosis than cutaneous melanoma. Surgery remains the primary therapeutic intervention for mucosal melanoma, and neoadjuvant therapy is still needed which can lead to improvements in outcomes by surgical resectability, local control and organ preservation.

Immunotherapy has showed promising activity in advanced melanoma. However, PD-1 antibodies, both Pembrolizumab and Nivolumab, showed much poorer response to mucosal melanoma than to cutaneous melanoma. Another anti-PD-1 antibody JS001(Toripalimab, Shanghai Junshi Biosciences) even did not show any response to mucosal melanoma (ORR 0%, NCT03013101). Surprisingly, JS001 in combination with Axitinib, a selective inhibitor of vascular endothelial growth factor receptors (VEGFR) 1, 2, and 3, had shown remarkable anti-tumor activity (ORR 60.6%) and manageable Adverse Events (Grade 3/4 AE 18%) in advanced mucosal melanoma (NCT03086174).

Pembrolizumab showed modest objective response rate (ORR, 13%) in advanced mucosal melanoma in Chinese population (NCT02628067). Recent study also demonstrated that neoadjuvant therapy with one dose of Pembrolizumab could lead to pathological complete response (pCR) or major response in nearly 30% of resectable advanced melanoma and pCR was associated with reduced risk of recurrence and improved survival. Lenvatinib is also a kinase inhibitor that inhibits the kinase activities of VEGFR1,2, 3 which has been approved by FDA for differentiated thyroid cancer, advanced renal cell carcinoma and hepatocellular carcinoma. Therefore, it is interesting to investigate whether combination of Pembrolizumab with Lenvatinib could improve pCR rate and consequent survival in resectable mucosal melanoma compared with neoadjuvant Pembrolizumab monotherapy.

Justification for Dose

Pembrolizumab

The planned dose of pembrolizumab for this study is 200 mg every 3 weeks (Q3W). Based on the totality of data generated in the Keytruda development program, 200 mg Q3W is the appropriate dose of pembrolizumab for adults across all indications and regardless of tumor type. As outlined below, this dose is justified by:

- Clinical data from 8 randomized studies demonstrating flat dose- and exposure-efficacy relationships from 2 mg/kg Q3W to 10 mg/kg every 2 weeks (Q2W),

- Clinical data showing meaningful improvement in benefit-risk including overall survival at 200 mg Q3W across multiple indications, and

- Pharmacology data showing full target saturation in both systemic circulation (inferred from pharmacokinetic [PK] data) and tumor (inferred from physiologically-based PK [PBPK] analysis) at 200 mg Q3W

Among the 8 randomized dose-comparison studies, a total of 2262 participants were enrolled with melanoma and non-small cell lung cancer (NSCLC), covering different disease settings (treatment naïve, previously treated, PD-L1 enriched, and all-comers) and different treatment settings (monotherapy and in combination with chemotherapy). Five studies compared 2 mg/kg Q3W versus 10 mg/kg Q2W (KN001 Cohort B2, KN001 Cohort D, KN002, KN010, and KN021), and 3 studies compared 10 mg/kg Q3W versus 10 mg/kg Q2W (KN001 Cohort B3, KN001 Cohort F2 and KN006). All of these studies demonstrated flat dose- and exposure-response relationships across the doses studied representing an approximate 5- to 7.5 fold difference in exposure. The 2 mg/kg (or 200 mg fixed-dose) Q3W provided similar responses to the highest doses studied. Subsequently, flat dose-exposure-response relationships were also observed in other tumor types including head and neck cancer, bladder cancer, gastric cancer and classical Hodgkin Lymphoma, confirming 200 mg Q3W as the appropriate dose independent of the tumor type. These findings are consistent with the mechanism of action of pembrolizumab, which acts by interaction with immune cells, and not via direct binding to cancer cells.

Additionally, pharmacology data clearly show target saturation at 200 mg Q3W. First, PK data in KN001 evaluating target-mediated drug disposition (TMDD) conclusively demonstrated saturation of PD-1 in systemic circulation at doses much lower than 200 mg Q3W. Second, a PBPK analysis was conducted to predict tumor PD-1 saturation over a wide range of tumor penetration and PD-1 expression. This evaluation concluded that pembrolizumab at 200 mg Q3W achieves full PD-1 saturation in both blood and tumor.

Finally, population PK analysis of pembrolizumab, which characterized the influence of body weight and other participant covariates on exposure, has shown that the fixed-dosing provides similar control of PK variability as weight based dosing, with considerable overlap in the distribution of exposures from the 200 mg Q3W fixed dose and 2 mg/kg Q3W dose. Supported by these PK characteristics, and given that fixed-dose has advantages of reduced dosing complexity and reduced potential of dosing errors, the 200 mg Q3W fixed-dose was selected for evaluation across all pembrolizumab protocols.

Lenvatinib

The dosing regimen of lenvatinib was selected based on the results of the Phase 1b portion of Study 111, the primary endpoint of which was to determine the MTD and RP2D for lenvatinib in combination with pembrolizumab 200 mg Q3W. Thirteen participants (lenvatinib 24 mg/day + pembrolizumab 200 mg IV Q3W: n=3; lenvatinib 20 mg/day +pembrolizumab 200 mg: n=10) were enrolled in the Phase 1b portion of the study. Eight of the participants had RCC, 2 had NSCLC, 2 had esophageal cancer, and 1 had melanoma. There were 2 dose-limiting toxicities (DLTs) at the lenvatinib 24 mg/day + pembrolizumab 200 mg dose IV Q3W (1 participant had Grade 3 arthralgia, and another had Grade 3 fatigue); hence, this was defined as the toxic dose.

Neither of these participants had melanoma. No DLTs were reported in the next 10 participants (expansion part), all of whom received the lenvatinib 20 mg/day plus pembrolizumab 200 mg Q3W dose. Based on review of all of the clinical data from these 23 participants, the MTD and RP2D were determined to be 20 mg lenvatinib daily in combination with a fixed dose of 200 mg pembrolizumab given

Q3W.

2.OBJECTIVES AND HYPOTHESES

2.1 Primary Objective and hypothesis

Objective: To evaluate the rate of pCR

Complete pathologic response(pCR)was defined as the absence of viable tumor based on hematoxylin and eosin (H&E) staining.

Hypothesis: Pembrolizumab in combination with lenvatinib will demonstrate higher pCR rate

2.2 Secondary Objectives and hypothesis

Objective: 1-year relapse-free survival (RFS) rate

Hypothesis: Pembrolizumab in combination with lenvatinib will result in higher 1-year RFS rate

Objective: Overall survival (OS)

Objective: Clinical Response

Objective: Major pathologic response rate

A major pathologic response was defined as less than 10% of viable tumor.

Objective: Surgical outcomes

a. Episodes of infection requiring antibiotics and/or wound drainage.
b. Duration of time from surgery end (time to recovery) to removal of wound drain because of ceased / minimal drainage
c. Episodes of seroma formation at the wound site requiring any intervention and volume of seroma drainage
d. Episodes of bleeding from the wound requiring a return to theatre and / or blood transfusion post-surgery.
e. Number of episodes (and patient number) of wound separation requiring any intervention

f. Incidence of lymphoedema or worsening lymphedema

g. An assessment of 'operability' at the time of surgery compared to the baseline evaluation.

h. The time from surgery completion to wound healing

Objective: Safety

3. STUDY DESIGN

3.1 Overall design

This is a single arm, open label study exploring the efficacy and safety of neoadjuvant Lenvatinib and Pembrolizumab in resectable mucosal Melanoma in China.

Approximately,26 patients will be enrolled into the study from Peking University Cancer Hospital.

Participants will receive Lenvatinib once a day (QD) for 6 weeks plus pembrolizumab on Day 1 of each 21-day cycle (Q3W) for 2 cycles in the neoadjuvant phase.

An assessment of response will be made after completing neoadjuvant therapy based on tumor imaging and endoscopy(if applicable).

If disease remains resectable based on the updated scans and the assessment of the treating surgical oncologist after neoadjuvant treatment, patients will undergo definitive surgical excision of visible disease. The window for performing surgery should be at least1 week, up to 4 weeks after the completion of neoadjuvant treatment.

If at any point during the course of neoadjuvant therapy there is clinical evidence of worsening of disease and/or objective evidence to suggest increase of disease dimensions regardless of whether the increase meets RECIST criteria, patient will be referred to the surgeon for review of the need to schedule surgery at an earlier time.

If surgery must be scheduled earlier for reasons other than disease progression or intolerable toxicity from study treatment, it is important to ensure that at least 2 doses of scheduled Pembrolizumab have been given.

Treatments should be discontinued for patients who refuse surgery or for whom surgery could be delay, per investigator's decision, for reasons other than adverse events; deterioration of health from melanoma (not related to progression of disease); deterioration of health from non-melanoma causes.

Pathologic assessment will be performed after surgery.

Initiation of adjuvant treatment should be no later than 6 weeks after completion of surgery. Patients will then receive 15 cycles Pembrolizumab 200mg, q3w maintenance treatment after surgery.

Following complete surgical resection of melanoma, patients will undergo CT scan of the same anatomical areas scanned prior to surgery and any further areas of concern every 12 weeks (+/- 7 days) until disease recurrence, death, lost to follow-up or patient withdrawal from the study, Additionally, endoscopy, if applicable, of the primary tumor site and MRI of the brain will be performed every 6 months(+/-1 month) and every year(+/-1 month) respectively.

Physical examinations will also be performed for both disease surveillance and monitoring of study treatment adverse events.

Participant who discontinue adjuvant therapy earlier for reasons other than disease recurrence will be followed with the same criteria until disease recurrence, death, lost to follow-up or patient withdrawal from the study.

All participants will be followed for survival until death, withdrawal of consent, or the end of study.

3.2 Study Duration

The study begins when the first participant signs the ICF and will last for approximately 2 years.

3.3 Participant Replacement Strategy

Any patients who discontinues from study treatment or withdraws from the study prior to the first dose of Pembrolizumab will be replaced unless where treatment related toxicities, disease progression or death have occurred.

,

3.4 Subject Withdrawal/Discontinuation Criteria

3.4.1 Discontinuation of Treatment

Discontinuation of treatment does not represent withdrawal from the trial.

As certain data on clinical events beyond treatment discontinuation may be important to the study, they must be collected through the subject's last scheduled follow-up, even if the subject has discontinued treatment. Therefore, all subjects who discontinue trial treatment prior to completion of the treatment will continue to participate in the trial.

Subjects may discontinue treatment at any time for any reason or be dropped from treatment at the discretion of the investigator should any untoward effect occur. In addition, a subject may be discontinued from treatment by the investigator if treatment is inappropriate, the trial plan is violated, or for administrative and/or other safety reasons.

A subject must be discontinued from treatment, but continue to be monitored in the trial for any of

the following reasons:

- The subject or subject's legally acceptable representative requests to discontinue treatment.
- Unacceptable adverse experiences
- Intercurrent illness that prevents further administration of treatment
- Investigator's decision to withdraw the subject from study treatment due to disease progression, recurrence or other reasons.
- The subject has a confirmed positive serum pregnancy test
- Noncompliance with trial treatment or procedure requirements

Following discontinuation of treatment, each subject will be followed for 30 days for any adverse events (SAEs will be collected for 90 days after completion of treatment or 30 days following completion of treatment if the subject initiates new anticancer therapy, whichever is earlier).

Discontinuation from treatment is "permanent." Once a subject is discontinued, he/she shall not be allowed to restart treatment.

3.4.2 Withdrawal from the Trial

A subject must be withdrawn from the trial if the subject or subject's legally acceptable representative withdraws consent from the trial.

If a subject withdraws from the trial, they will no longer receive treatment or be followed at scheduled protocol visits.

3.5 Eligibility

3.5.1 Inclusion criteria

1. Be willing and able to provide written informed consent for the trial.
2. Be a male or female subject and is 18-75 years of age on day of signing informed consent.
3. Have histologically or cytologically confirmed resectable mucosal melanoma. Only cases where a complete surgical resection with tumour-free margins can safely be achieved, as assessed by the surgeon, are defined as resectable.
4. Newly diagnosed melanoma without any previous anti-cancer treatment
5. Patients must be able to provide a biopsy at baseline
6. Able to swallow and retain oral medication

7. Be medically fit enough to undergo surgery as determined by the treating medical and surgical oncology team
8. Have Eastern Cooperative Oncology Group (ECOG) performance status of 0 or 1 performed within 10 days of treatment initiation.
9. Demonstrate adequate organ function as defined in Table 1. All screening labs should be performed within 1 week prior to treatment initiation.
10. Adequately controlled blood pressure (BP) with or without antihypertensive medications, defined as BP ⩽ 150/90 mmHg at screening and no change in antihypertensive medications within 1 week before treatment initiation.
11. Males and female subjects of childbearing potential must be willing to use an adequate method of contraception, for the course of the study through 120 days after the last dose of study medication.
12. (Female subject of childbearing potential) Have a negative urine or serum pregnancy test within 1 week prior to receiving the first dose of study medication. If the urine test is positive or borderline a serum pregnancy test will be required.

3.5.2 Exclusion criteria

1. Has a known additional malignancy that is progressing or requires active treatment, except for adequately treated basal cell or squamous cell skin cancer or in situ cervical cancer.
2. Has known central nervous system (CNS) metastases and/or carcinomatous meningitis.
3. Has received any prior systemic anti-cancer treatment for melanoma
4. Has received prior lenvatinib
5. Has received prior therapy with an anti-PD-1 or anti-PD-L1 agent
6. Is currently participating in or has participated in an interventional clinical trial with an investigational compound or device within 4 weeks of the first dose of treatment in this current trial.

   Note: subject should be excluded if he/she received an investigational agent with anti-cancer or anti-proliferative intent within the last 12 months.
7. Has received a live vaccine within 30 days of the first dose of study treatment.

   Note: Seasonal influenza vaccines for injection are generally inactivated flu vaccines and are allowed; however intranasal influenza vaccines (e.g., FluMist ®) are live attenuated vaccines, and are not allowed.
8. Has an active autoimmune disease that has required systemic treatment in past 2 years (i.e., with use of disease modifying agents, corticosteroids or immunosuppressive drugs).

Replacement therapy (e.g., thyroxine, insulin, or physiologic corticosteroid replacement therapy for adrenal or pituitary insufficiency) is not considered a form of systemic treatment.

9. Has a diagnosis of immunodeficiency or is receiving systemic steroid therapy (in dosing exceeding 10mg daily of prednisone equivalent) or any other form of immunosuppressive therapy within 1 week prior to the first dose of trial treatment.

10. Has a known history of Human Immunodeficiency Virus (HIV) (HIV 1/2 antibodies).

11. Has known active Hepatitis B (e.g., HBsAg reactive) or Hepatitis C (e.g., HCV RNA [qualitative] is detected).

12. Has a history of (non-infectious) pneumonitis that required steroids or current pneumonitis

13. Has an active infection requiring systemic therapy.

14. Has presence of gastrointestinal condition including malabsorption, gastrointestinal anastomosis, or any other condition that might affect the absorption of lenvatinib.

15. Has had a major surgery within 4 weeks prior to initiation of treatment. Adequate wound healing after major surgery must be assessed clinically and have resolved completely.

16. Has a pre-existing Grade ≥3 gastrointestinal or non-gastrointestinal fistula.

17. Has radiographic evidence of major blood vessel invasion/infiltration. The degree of tumor invasion/infiltration of major blood vessels should be considered because of the potential risk of severe hemorrhage associated with tumor shrinkage/necrosis following lenvatinib therapy.

18. Has clinically significant hemoptysis or tumor bleeding within 2 weeks prior to the first dose of study drug.

19. Has clinically significant cardiovascular disease within 12 months of the first dose of study intervention including New York Heart Association Class III or IV congestive heart failure, unstable angina, myocardial infarction, cerebral vascular accident, or cardiac arrhythmia associated with hemodynamic instability.

    Note: Medically controlled arrhythmia would be permitted.

20. Has urine protein ≥1 g/24-hour.

    Note: Participants with >1+ proteinuria on urine dipstick will undergo 24-hour urine collection for quantitative assessment of proteinuria.

21. Has prolongation of QTc interval (calculated using Fridericia's formula) to >480 msec.

22. Has left ventricular ejection fraction (LVEF) below the institutional normal range as determined by multi-gated acquisition scan (MUGA) or echocardiogram.

23. Has a history or current evidence of any condition, therapy, lab abnormality or other circumstance that might expose the subject to risk by participating in the trial, confound the results of the trial, or interfere with the subject's participation for the full duration of the trial.

24. Has known psychiatric or substance abuse disorders that would interfere with cooperation with the requirements of the trial.

25. Is pregnant or breastfeeding or expecting to conceive children within the projected duration of the trial, starting with the screening visit through 6 months after the last dose of study medication.

26. Has a known hypersensitivity to the components of the study therapy or its analogs.

27. Has a known history of active TB (Bacillus Tuberculosis)

28. Female participants who are breastfeeding are not eligible for enrollment

Table Adequate Organ Function Laboratory Values

System	Laboratory Value
Hematological	
Absolute neutrophil count (ANC)	⩾1500/μL
Platelets	⩾100 000/μL
Hemoglobin	⩾9.0 g/dL or ⩾5.6 mmol/L[1]
Renal	
Creatinine OR Measured or calculated[2] creatinine clearance (GFR can also be used in place of creatinine or CrCl)	⩽1.5 × ULN OR ⩾30 mL/min for participant with creatinine levels >1.5 × institutional ULN
Hepatic	
Total bilirubin	⩽ 1.5 ×ULN OR direct bilirubin ⩽ULN for participants with total bilirubin levels >1.5 × ULN except for unconjugated hyperbilirubinemia of Gilbert's syndrome.
AST (SGOT) and ALT (SGPT), and ALP	⩽ 2.5 × ULN (⩽ 5 × ULN for participants with liver metastases)[3]
Coagulation	
International normalized ratio (INR) OR prothrombin time (PT) Activated partial thromboplastin time (aPTT)/PTT	⩽ 1.5 × ULN unless participant is receiving anticoagulant therapy as long as PT or PTT is within therapeutic range of intended use of anticoagulants

[1]Criteria must be met without erythropoietin dependency and without packed red blood cell (pRBC) transfusion within last 2 weeks.
[2]Creatinine clearance (CrCl) should be calculated per institutional standard.
[3]Participants with ALP values >3 times the ULN and known to have bone metastases can be included. Note: This table includes eligibility-defining laboratory value requirements for treatment; laboratory value requirements should be adapted according to local regulations and

guidelines for the administration of specific chemotherapies.
Abbreviations: ALT (SGPT)=alanine aminotransferase (serum glutamic pyruvic transaminase); ALP: alkaline phosphatase; AST (SGOT)=aspartate aminotransferase (serum glutamic oxaloacetic transaminase); CrCl =creatinine clearance; GFR=glomerular filtration rate; ULN=upper limit of normal.

4.STUDY INTERVENSION

4.1 Study Intervention(s) Administered

Lenvatinib will be administered 20mg orally once a day (QD) for 6 weeks during the neoadjuvant phase.

Pembrolizumab will be administered at a fix dose of 200 mg by intravenous (IV) infusion on Day 1 of each 21-day cycle (Q3W) for 2 cycles during the neoadjuvant phase and 17 cycles following surgery.

4.2 Dose Modification

4.2.1 Pembrolizumab

Adverse events (both non-serious and serious) associated with pembrolizumab exposure may represent an immunologic etiology. These AEs may occur shortly after the first dose or several months after the last dose of treatment. Pembrolizumab must be withheld for drug related toxicities or severe or life-threatening AEs as per Table below. See Rescue Medications & Supportive Care for Supportive Care Guidelines, including use of corticosteroids.

Table Dose Modification Guidelines for Drug-Related Adverse Events (Pembrolizumab)

Toxicity	Hold Treatment For Grade	Timing for Restarting Treatment	Treatment Discontinuation
Diarrhea/Colitis	2-3	Toxicity resolves to Grade 0-1.	Toxicity does not resolve within 6 weeks of last dose or inability to reduce corticosteroid to 10 mg or less of prednisone or equivalent per day

			within 6 weeks.
	4	Permanently discontinue	Permanently discontinue
AST, ALT, or Increased Bilirubin	2	Toxicity resolves to Grade 0-1	Toxicity does not resolve within 6 weeks of last dose.
	3-4	Permanently discontinue	Permanently discontinue
Type 1 diabetes mellitus (if new onset) including<br>Diabetic ketoacidosis (DKA) or Hyperglycemia (≥ Grade 3) if associated with ketosis (ketonuria) or metabolic acidosis	T1DM or 3-4	Hold pembrolizumab for new onset Type 1 diabetes mellitus or Grade 3-4 hyperglycemia associated with evidence of beta cell failure.	Resume pembrolizumab when patients are clinically and metabolically stable.
Hypophysitis	2-4	Toxicity resolves to Grade 0-1. Therapy with pembrolizumab can be continued while endocrine replacement therapy is instituted	Toxicity does not resolve within 6 weeks of last dose or inability to reduce corticosteroid to 10 mg or less of prednisone or equivalent per day within 6 weeks.
Hyperthyroidism	3	Toxicity resolves to Grade 0-1	Toxicity does not resolve within 6 weeks of last dose or inability to reduce corticosteroid to 10 mg or less of prednisone or equivalent per day within 6 weeks.
	4	Permanently discontinue	Permanently discontinue
Hypothyroidism		Therapy with pembrolizumab can	Therapy with pembrolizumab can

		be continued while thyroid replacement therapy is instituted	be continued while thyroid replacement therapy is instituted.
Infusion Reaction	3-4	Permanently discontinue	Permanently discontinue
Pneumonitis	2	Toxicity resolves to Grade 0-1	Toxicity does not resolve within 6 weeks of last dose or inability to reduce corticosteroid to 10 mg or less of prednisone or equivalent per day within 6 weeks.
	3-4 or recurrent 2	Permanently discontinue	Permanently discontinue
Renal Failure or Nephritis	2	Toxicity resolves to Grade 0-1	Toxicity does not resolve within 6 weeks of last dose or inability to reduce corticosteroid to 10 mg or less of prednisone or equivalent per day within 6 weeks.
	3-4	Permanently discontinue	Permanently discontinue
All Other Drug-Related Toxicity[1]	3 or Severe	Toxicity resolves to Grade 0-1	Toxicity does not resolve within 6 weeks of last dose or inability to reduce corticosteroid to 10 mg or less of prednisone or equivalent per day within 6 weeks.
	4	Permanently discontinue	Permanently discontinue

Note: Permanently discontinue for any severe or Grade 3 (Grade 2 for recurrent pneumonitis)

drug-related AE that recurs or any life-threatening event.

[1]Patients with intolerable or persistent Grade 2 drug-related AE may hold study medication at physician discretion. Permanently discontinue study drug for persistent Grade 2 adverse reactions for which treatment with study drug has been held, that do not recover to Grade 0-1 within 6 weeks of the last dose.

Dosing interruptions are permitted in the case of medical/surgical events or logistical reasons not related to study therapy (e.g., elective surgery, unrelated medical events, patient vacation, and/or holidays). Subjects should resume study therapy within 6 weeks of the scheduled interruption, unless otherwise discussed with the investigator. The reason for interruption should be documented in the patient's study record.

4.2.2 Lenvatinib

Lenvatinib dose reduction and interruption for participants who experience lenvatinib-pembrolizumab combination therapy-related toxicity will be in accordance with the dose modification guidelines described in Table. An interruption of study treatment for more than 28 days will require additional approval before treatment can be resumed.

The starting dose of lenvatinib is 20 mg/day. Dose reductions of lenvatinib occur in succession based on the previous dose level (14, 10, and 8 mg/day). Any dose reduction below 8 mg/day must be discussed. Once the study drug dose has been reduced, it may not be increased at a later date, unless the dose has been mistakenly decreased; in this situation, additional approval is required to increase the dose.

Table Dose Modification Guidelines for Lenvatinib-related Adverse Events

Treatment-related Toxicity[a,b]	Management	Dose Adjustment
Grade 1 or Tolerable Grade 2		
	Continue treatment	No change
Intolerable Grade 2[c, d] or Grade 3[e]		
First occurrence	Interrupt lenvatinib until resolved to Grade 0-1, or tolerable Grade 2	Reduce lenvatinib dose to 14mg once a day based on starting dose (1-level reduction)
Second occurrence (same toxicity or new toxicity)	Interrupt lenvatinib until resolved to Grade 0-1, or tolerable Grade 2	Reduce lenvatinib dose to 10 mg once a day (2-level reduction);
Third occurrence (same toxicity or new toxicity)	Interrupt lenvatinib until resolved to Grade 0-1, or tolerable Grade 2	Reduce lenvatinib dose to 8 mg orally once a day (1-level reduction)
Fourth occurrence (same toxicity or new toxicity)	Interrupt lenvatinib	Discussion
Grade 4[f]: Discontinue Study Treatment		

Note: For grading see CTCAE version 4.0. Collect all AE grades (ie, decreasing and increasing CTCAE grade).

[a] An interruption of study treatment for more than 28 days will be discussed before treatment can be resumed.

[b] Initiate optimal medical management for nausea, vomiting, hypertension, hypothyroidism and/or diarrhea prior to any lenvatinib interruption or dose reduction.

[c] Applicable only to Grade 2 toxicities judged by the participant and/or physician to be intolerable.

[d] Obese participants (BMI ≥30) with weight loss do not need to return to their baseline weight or within 10% of their baseline weight (ie, Grade 1 weight loss). These participants may restart study treatment at a lower dose once their weight remains stable for at least 1 week, and they reach at least a BMI of 25. The new stable weight should be used as the new baseline for further dose reductions.

[e] For asymptomatic laboratory abnormalities, such as Grade ≥3 elevations of amylase and lipase that are not considered clinically relevant by the investigator, continuation of treatment should be discussed with Sponsor.

[f] Excluding laboratory abnormalities judged to be non-life-threatening, in which case manage as Grade 3.

Abbreviations: AE = adverse event; BMI = body mass index; CTCAE = Common Terminology Criteria for Adverse Events.

4.2.3 Dose Modifications for Overlapping Toxicities

Based on the known toxicity profiles of pembrolizumab and lenvatinib, certain treatment related AEs are uniquely associated with one drug versus the other. For example, hypertension, arterial thrombotic events, proteinuria, and hemorrhagic events are known risks for lenvatinib treatment, while immune-related AEs are risks for pembrolizumab treatment. However, certain AEs, such as diarrhea, hypothyroidism, and liver enzyme elevation, may be initially considered attributable to either study drug. Therefore, evaluation of attribution is important for determining the study drug most likely related to the AE, or an alternative etiology, and subsequently proper clinical management. The following aspects should be considered:

1. Timing of AE onset

Since lenvatinib is dosed daily and continuously due to a relatively short half-life (~28 hours), and pembrolizumab is dosed Q3W due to a long half-life, lenvatinib can be interrupted to assess whether an AE improves/resolves with dechallenge (ie, interruption of treatment) based on the following 2 scenarios:

- If an AE is identified during a treatment cycle (ie, between 2 pembrolizumab doses), only lenvatinib dose interruption is needed.
- If an AE is identified at the beginning of a treatment cycle, lenvatinib can be interrupted and dosing of pembrolizumab should be held.

If the participant recovers from an AE in response to lenvatinib interruption (ie, positive dechallenge), the event is more likely to be related to lenvatinib. Otherwise, after excluding other alternative explanations, an immune-related AE should be considered.

2. Severity of AE

If an AE is suspected to be treatment related and is severe/life threatening at the time of onset or is rapidly worsened, action including interrupting both drugs and initiating treatment with a corticosteroid (with exception of hypothyroidism, TIDM) and other supportive care should be taken promptly.

4.3 Rescue Medications and Supportive Care

4.3.1 Supportive Care Guidelines for Pembrolizumab

Subjects should receive appropriate supportive care measures as deemed necessary by the treating investigator. Suggested supportive care measures for the management of adverse events with potential immunologic etiology are outlined below. Where appropriate, these guidelines include the use of oral or intravenous treatment with corticosteroids as well as additional anti-inflammatory agents if symptoms do not improve with administration of corticosteroids. Note that several courses of steroid tapering may be necessary as symptoms may worsen when the steroid dose is decreased. For each disorder, attempts should be made to rule out other causes such as metastatic disease or bacterial or viral infection, which might require additional supportive care. The treatment guidelines are intended to be applied when the investigator determines the events to be related to pembrolizumab.

Refer to dose modification.

It may be necessary to perform conditional procedures such as bronchoscopy, endoscopy, or skin photography as part of evaluation of the event.

Pneumonitis:

- For Grade 2 events, treat with systemic corticosteroids. When symptoms improve to Grade 1 or less, steroid taper should be started and continued over no les s than 4 weeks.
- For Grade 3-4 events, immediately treat with intravenous steroids. Administer additional anti-inflammatory measures, as needed.
- Add prophylactic antibiotics for opportunistic infections in the case of prolonged steroid administration.

Diarrhea/Colitis:

Subjects should be carefully monitored for signs and symptoms of enterocolitis (such as diarrhea, abdominal pain, blood or mucus in stool, with or without fever) and of bowel perforation (such as peritoneal signs and ileus).

- All subjects who experience diarrhea/colitis should be advised to drink liberal quantities of clear fluids. If sufficient oral fluid intake is not feasible, fluid and electrolytes should be substituted via IV infusion. For Grade 2 or higher diarrhea, consider GI consultation and endoscopy to confirm or rule out colitis.
- For Grade 2 diarrhea/colitis, administer oral corticosteroids.
- For Grade 3 or 4 diarrhea/colitis, treat with intravenous steroids followed by high dose oral steroids.
- When symptoms improve to Grade 1 or less, steroid taper should be started and continued over no less than 4 weeks.

Type 1 diabetes mellitus (T1DM) (if new onset, including diabetic ketoacidosis [DKA]) or ≥ Grade 3 Hyperglycemia, if associated with ketosis (ketonuria) or metabolic acidosis (DKA)

For T1DM or Grade 3-4 Hyperglycemia

- Insulin replacement therapy is recommended for Type I diabetes mellitus and for Grade 3-4 hyperglycemia associated with metabolic acidosis or ketonuria.
- Evaluate patients with serum glucose and a metabolic panel, urine ketones, glycosylated hemoglobin, and C-peptide.

Hypophysitis:

- For Grade 2 events, treat with corticosteroids. When symptoms improve to Grade 1 or less, steroid taper should be started and continued over no less than 4 weeks. Replacement of appropriate hormones may be required as the steroid dose is tapered.
- For Grade 3-4 events, treat with an initial dose of IV corticosteroids followed by oral corticosteroids. When symptoms improve to Grade 1 or less, steroid taper should be started and continued over no less than 4 weeks. Replacement of appropriate hormones may be required as the steroid dose is tapered.

Hyperthyroidism or Hypothyroidism:

Thyroid disorders can occur at any time during treatment. Monitor patients for changes in thyroid function (at the start of treatment, periodically during treatment, and as indicated based on clinical evaluation) and for clinical signs and symptoms of thyroid disorders.

- Grade 2 hyperthyroidism events (and Grade 2-4 hypothyroidism):
  - In hyperthyroidism, non-selective beta-blockers (e.g. propranolol) are suggested as initial therapy.
  - In hypothyroidism, thyroid hormone replacement therapy, with levothyroxine or liothyronine, is indicated per standard of care.
- Grade 3-4 hyperthyroidism
  - Treat with an initial dose of IV corticosteroid followed by oral corticosteroids. When symptoms improve to Grade 1 or less, steroid taper should be started and continued over

no less than 4 weeks. Replacement of appropriate hormones may be required as the steroid dose is tapered.

Hepatic:

- For Grade 2 events, monitor liver function tests more frequently until returned to baseline values (consider weekly).
    - Treat with IV or oral corticosteroids
- For Grade 3-4 events, treat with intravenous corticosteroids for 24 to 48 hours.
- When symptoms improve to Grade 1 or less, a steroid taper should be started and continued over no less than 4 weeks.

Renal Failure or Nephritis:

- For Grade 2 events, treat with corticosteroids.
- For Grade 3-4 events, treat with systemic corticosteroids.
- When symptoms improve to Grade 1 or less, steroid taper should be started and continued over no less than 4 weeks.

Management of Infusion Reactions:

Signs and symptoms usually develop during or shortly after drug infusion and generally resolve completely within 24 hours of completion of infusion. Table 6 shows treatment guidelines for subjects who experience an infusion reaction associated with administration of pembrolizumab.

Table Infusion Reaction Treatment Guidelines

NCI CTCAE Grade	Treatment	Premedication at subsequent dosing
Grade 1<br>Mild reaction; infusion interruption not indicated; intervention not indicated	Increase monitoring of vital signs as medically indicated until the subject is deemed medically stable in the opinion of the investigator.	None
Grade 2<br>Requires infusion interruption but responds promptly to symptomatic treatment (e.g., antihistamines, NSAIDS, narcotics, IV fluids); prophylactic medications indicated for ≤24 hrs	Stop Infusion.<br>Additional appropriate medical therapy may include but is not limited to:<br>IV fluids<br>Antihistamines<br>NSAIDS<br>Acetaminophen<br>Narcotics<br>Increase monitoring of vital signs as medically indicated until the subject is deemed	Subject may be premedicated 1.5 h (± 30 minutes) prior to infusion of pembrolizumab with:<br>Diphenhydramine 50 mg p.o.(or equivalent dose of antihistamine).<br>Acetaminophen 500-1000 mg p.o. (or equivalent dose of antipyretic).

	medically stable in the opinion of the investigator.<br>If symptoms resolve within one hour of stopping drug infusion, the infusion may be restarted at 50% of the original infusion rate (e.g. from 100 mL/hr to 50 mL/hr). Otherwise dosing will be held until symptoms resolve and the subject should be premedicated for the next scheduled dose.	
Grades 3 or 4<br>Grade 3:<br>Prolonged (i.e., not rapidly responsive to symptomatic medication and/or brief interruption of infusion); recurrence of symptoms following initial improvement; hospitalization indicated for other clinical sequelae (e.g., renal impairment, pulmonary infiltrates)<br>Grade 4:<br>Life-threatening; pressor or ventilatory support indicated	Stop Infusion.<br>Additional appropriate medical therapy may include but is not limited to:<br>IV fluids<br>Antihistamines<br>NSAIDS<br>Acetaminophen<br>Narcotics<br>Oxygen<br>Pressors<br>Corticosteroids<br>Epinephrine<br>Increase monitoring of vital signs as medically indicated until the subject is deemed medically stable in the opinion of the investigator.<br>Hospitalization may be indicated.<br>Subject is permanently discontinued from further trial treatment administration.	No subsequent dosing

Appropriate resuscitation equipment should be available in the room and a physician readily available during the period of drug administration.

For Further information, please refer to the Common Terminology Criteria for Adverse Events (CTCAE)v 4.0 at http://ctep.cancer.gov

4.3.2 Supportive Care Guidelines for Lenvatinib

Management of Hypertension

Investigators should therefore ensure that participants enrolled to receive treatment with lenvatinib have BP of ≤150/90 mm Hg at the time of study entry and, if known to be hypertensive, have been on a stable dose of antihypertensive therapy for at least 1 week before C1D1. Early detection and effective management of hypertension are important to minimize the need for lenvatinib dose interruptions and reductions.

Regular assessment of BP should be as detailed in the SoA. Hypertension will be graded using NCI CTCAE v4.0, based on BP measurements only (and not on the number of antihypertensive medications).

If the participant's initial BP measurement is elevated (ie, systolic BP ≥140 mm Hg or diastolic BP ≥90 mm Hg), the BP measurement should be repeated at least 5 minutes later. One BP assessment is defined as the mean value of 2 measurements at least 5 minutes apart. If the BP assessment (ie, the mean of the 2 BP measurements obtained at least 5 minutes apart) is elevated (systolic BP ≥140 mm Hg or diastolic BP ≥90 mm Hg), a confirmatory assessment should be obtained at least 30 minutes later by performing 2 measurements (at least 5 minutes apart) to yield a mean value.

Antihypertensive agents should be started as soon as elevated BP (systolic BP ≥140 mm Hg or diastolic BP ≥90 mm Hg) is confirmed on 2 assessments at least 30 minutes apart. The choice of antihypertensive treatment should be individualized to the participant's clinical circumstances and follow standard medical practice. For previously normotensive participants, appropriate antihypertensive therapy should be started when systolic BP ≥140 mm Hg or diastolic BP ≥90 mm Hg is first observed on 2 assessments at least 30 minutes apart. For those participants already on antihypertensive medication, treatment modification may be necessary if hypertension persists.

Lenvatinib should be withheld in any instance where a participant is at imminent risk to develop a hypertensive crisis or has significant risk factors for severe complications of uncontrolled hypertension (eg, BP ≥160/100 mm Hg, significant risk factors for cardiac disease, intracerebral hemorrhage, or other significant co-morbidities). Once the participant has been on the same antihypertensive medications for at least 48 hours and the BP is controlled, lenvatinib should be resumed as described below.

Participants who have had systolic BP ≥160 mm Hg or diastolic BP ≥100 mm Hg must have their BP monitored on Day 15 (or more frequently as clinically indicated) until systolic BP has been ≤150 mm Hg and diastolic BP has been ≤95 mm Hg for 2 consecutive treatment cycles. If a repeat event of systolic BP ≥160 mm Hg or diastolic BP ≥100 mm Hg occurs, the participant must resume the Day 15 evaluation until systolic BP has been ≤150 mm Hg and diastolic BP has been ≤95 mm Hg for 2 consecutive treatment cycles.

The following guidelines should be followed for the management of systolic BP ≥160 mm Hg or diastolic BP ≥100 mm Hg confirmed on 2 BP assessments at least 30 minutes apart:

1. Continue study drug and institute antihypertensive therapy for participants not already receiving antihypertensive therapy. Note: Eligibility of a participant that is receiving ≥ 3 antihypertensive medications prior to study entry will require Sponsor approval.

2. For those participants already on antihypertensive medication, the dose of the current agent may be increased, if appropriate, or 1 or more agents of a different class of antihypertensive should be added. Study treatment can be continued without dose modification.

3. If systolic BP ≥160 mm Hg or diastolic BP ≥100 mm Hg persists despite maximal antihypertensive therapy, then lenvatinib administration should be interrupted and restarted at 1 dose level reduction only when systolic BP ≤150 mm Hg and diastolic BP ≤95 mm Hg and the participant has been on a stable dose of antihypertensive medication for at least 48 hours.

    - If systolic BP ≥160 mm Hg or diastolic BP ≥100 mm Hg recurs on the first dose reduction despite optimal management of hypertension with antihypertensive medications (either by dose increase or the addition of a different class of antihypertensive), then lenvatinib administration should be interrupted and restarted at an additional dose reduction only when systolic BP ≤150 mm Hg and diastolic BP ≤95 mm Hg and the participant has been on a stable dose of antihypertensive medication for at least 48 hours.
    - If systolic BP ≥160 mm Hg or diastolic BP ≥100 mm Hg recurs on the second dose reduction despite optimal management of hypertension with antihypertensive medications (either by dose increase or the addition of a different class of antihypertensive), then lenvatinib administration should be interrupted and restarted at a third dose reduction only when systolic BP ≤150 mm Hg and diastolic BP ≤95 mm Hg and the participant has been on a stable dose of antihypertensive medication for at least 48 hours.
    - Additional dose reduction should be discussed with Sponsor.

The following guidelines should be followed for the management of Grade 4 hypertension (life-threatening consequences):

1. Institute appropriate medical management
2. Discontinue study drug

<u>Management of Proteinuria</u>

Regular assessment of proteinuria should be conducted as detailed in the SoA. Guidelines for assessment and management of proteinuria are as follows:

Detection and Confirmation:

- Perform urine dipstick testing per the SoA
- A 24-hour urine collection initiated as soon as possible and at least within 72 hours (or

an immediate spot urine protein-to-creatinine ratio [UPCR] test) is required in the following situations:

- The first (initial) occurrence of ≥2+ proteinuria on urine dipstick while on study drug

- A subsequent increase in severity of urine dipstick proteinuria occurring on the

same lenvatinib dose level

- When there has been a lenvatinib dose reduction and at the new dose level the urine protein dipstick result is ≥2+.

- A 24-hour urine collection (initiated as soon as possible and at least within 72 hours) to verify the grade of proteinuria is required when UPCR is ≥2.4.

Grading of Proteinuria:

- Grading according to NCI CTCAE v4.0 will be based on the 24-hour urinary protein result if one has been obtained. Management of lenvatinib administration will be based on the grade of proteinuria according to Dose modification Table:
- In the event of nephrotic syndrome, lenvatinib must be discontinued.

Monitoring:

- Urine dipstick testing for participants with proteinuria ≥2+ should be performed on D15 (or more frequently as clinically indicated) until the results have been 1+ or negative for 2 consecutive treatment cycles.
- Proteinuria monitoring can be performed at the local laboratory or investigator site but must be managed by the site physician.

Management of Diarrhea

An anti-diarrheal agent should be recommended to the participant at the start of study treatment and participants should be instructed and educated to initiate anti-diarrheal treatment at the first onset of soft bowel movements. The choice of anti-diarrheal agent should be individualized to the participant's clinical circumstances and follow standard medical practice. If signs/symptoms of diarrhea persist despite optimal medical management,

instructions contained in Dose modification Table should be followed.

Management of Hepatotoxicity

Liver function tests (alanine transaminase [ALT], aspartate transaminase [AST], bilirubin levels) should be conducted as detailed in the SoA and as clinically indicated. If signs/symptoms indicating liver injury occur, instructions contained in Dose modification Table should be followed. Appropriate supportive care should be provided together with close monitoring. If

hepatic failure occurs, the study drug must be discontinued.

Management of Thromboembolic Events

Participants should be advised to pay attention to symptoms suggestive of venous thromboembolic

events which include acute onset of shortness of breath, dyspnea, chest pain, cough, hemoptysis, tachypnea, tachycardia, cyanosis, and DVT signs including lower-extremity swelling and warmth to touch or tenderness. In case any of these symptoms appear, participants should be instructed to report such symptoms promptly to the treating physician. If a thromboembolic event is confirmed, instructions contained in Dose modification Table should be followed. Appropriate supportive care should be provided together with close monitoring. Lenvatinib must be discontinued for a Grade 3 thromboembolic event that requires urgent intervention. If a participant experiences life-threatening (Grade 4) thromboembolic reactions, including pulmonary embolism, the study drug must be discontinued.

Arterial thromboembolic events (eg, new onset, worsening, or unstable angina, myocardial infarction, transient ischemic attack, and cerebrovascular accident) of any grade require study treatment discontinuation.

Management of Posterior Reversible Encephalopathy Syndrome/Reversible Encephalopathy Syndrome/Reversible Posterior Leukoencephalopathy Syndrome

Posterior Reversible Encephalopathy Syndrome/Reversible Encephalopathy Syndrome/ Reversible Posterior Leukoencephalopathy Syndrome (PRES/RPLS) is a neurological disorder that can present with headache, seizure, lethargy, confusion, altered mental function, blindness, and other visual or neurological disturbances. Mild to severe hypertension may be present. MRI is necessary to confirm the diagnosis of PRES/RPLS. Appropriate measures should be taken to control BP. In participants with signs or symptoms of PRES/RPLS, instructions in Dose modification Table should be followed.

Management of Hypocalcemia

Serum calcium should be monitored per the SoA. Corrected serum calcium should be used to assess the grade of hypocalcemia per CTCAE v4.0, using the following formula:

Corrected calcium = ([4 − serum albumin in g/dL] × 0.8 + serum calcium).

The formula is not applicable when serum albumin concentration is normal (>4 g/dL); in such situations, the total (uncorrected) serum calcium should be used instead.

Hypocalcemia should be treated per institutional guidelines (eg, using appropriate calcium, magnesium, and vitamin D supplementation) until resolution.

Management of Hemorrhage

Instructions in Dose Modification Table should be followed for the management of hemorrhage. Either resume at a reduced dose or discontinue lenvatinib depending on the severity and persistence of hemorrhage.

4.4 Concomitant Therapy

Medications or vaccinations specifically prohibited in the exclusion criteria are not allowed during the treatment period. If there is a clinical indication for any medication or vaccination specifically prohibited, discontinuation from study treatment may be required. The final decision on any

supportive therapy or vaccination rests with the investigator. However, the decision to continue the participant on study treatment requires the mutual agreement of the investigator and the participant.

Any medication (including over-the-counter medications) or therapy administered to the participant during the study (starting at the date of informed consent) will be recorded on the appropriate case report form (CRF). The investigator will record the AE for which the concomitant medication/therapy was administered on the appropriate CRF. If the concomitant medication/therapy is being administered for a medical condition present at the time of entry into the study, the investigator will record the medical condition on the appropriate CRF.

All prior medications (including over-the-counter medications) administered 30 days before the first dose of study drug and any concomitant therapy administered to the participant during the course of the study (starting at the date of informed consent) until 30 days after the final dose of study drug will be recorded. Additionally, all diagnostic, therapeutic, or surgical procedures relating to malignancy should be recorded. Any medication that is considered necessary for the participant's health and that is not expected to interfere with the evaluation of or interact with the study medication may be continued during the study.

4.4.1 Allowed Concomitant Medication(s)

Treatment of complications or AEs, or therapy to ameliorate symptoms (including blood products, blood transfusions, fluid transfusions, antibiotics, and antidiarrheal drugs), may be given at the discretion of the investigator, unless it is expected to interfere with the evaluation of (or to interact with) the study medication. Anti-emetic or any other prophylaxis should be considered in accordance with institutional guidelines. Bisphosphonates (including denosumab) are also allowed.

4.4.2 Prohibited Concomitant Medication(s)

Participants are prohibited from receiving the following therapies during the Screening and Treatment Phase of this study:

• Concurrent anticancer therapies such as chemotherapy, other targeted therapies (eg, tyrosine kinase inhibitors), anti-tumor interventions (surgical resection, surgical debulking of tumor, etc.), or cancer immunotherapy not specified in this protocol.

• Other concurrent investigational drugs.

• Live vaccines within 30 days and while participating in the study. Examples of live vaccines include, but are not limited to, the following: measles, mumps, rubella, chicken pox, yellow fever, rabies, BCG, and typhoid (oral) vaccine. Seasonal influenza vaccines for injection are generally killed virus vaccines and are allowed. However, intranasal influenza vaccines (eg, Flu-Mist®) are live attenuated vaccines, and are not allowed.

• Systemic glucocorticoids for any purpose other than to modulate symptoms from an AE that is

suspected to have immunologic etiology. Physiologic doses of corticosteroids not exceeding 10 mg daily of prednisone equivalent may be used during the study.

Note: Inhaled steroids are allowed for management of asthma or seasonal allergies.

• Adjuvant radiotherapy is not allowed unless surgical margins are found to be positive.

For participants who, in an assessment by the investigator, require the use of any of the afore mentioned treatments for clinical management, continuation of the study medication and further participation in the study must be discussed.

If participants receive additional anticancer therapies, this will be judged to represent evidence of disease progression or recurrence, and study medication will be discontinued. These participants should complete all end of treatment assessments and continue to be followed for survival in the Follow-up Period.

4.4.3 Drug Interactions

Lenvatinib's weak in vitro inhibitory and induction potential on cytochrome P450 (CYP) enzymes (Study No. XT063020) suggests a low risk of lenvatinib interference with the PK of other drugs metabolized by CYP enzymes which are co-administered in usual clinic practice.

Nonclinical studies identify CYP3A4 as the important CYP isozyme responsible for human hepatic metabolism of lenvatinib. However, clinical studies conducted showed that coadministration of lenvatinib with either inducers or inhibitors of CYP3A4/P-glycoprotein are not of clinical concern. The main metabolic pathways for lenvatinib in humans were identified as enzymatic (CYP3A and aldehyde oxidase) and non-enzymatic processes (LENVIMA® Package Insert).

No formal PK drug interaction studies have been conducted with pembrolizumab.

Pembrolizumab is a mAb; PK interactions with lenvatinib (and vice-versa) are not expected.

5. STUDY ASSESSMENTS AND PROCEDURES

The Schedule of Event-Appendix summarizes the trial procedures to be performed at each visit. Individual trial procedures are described in detail below. It may be necessary to perform these procedures at unscheduled time points if deemed clinically necessary by the investigator.

Furthermore, additional evaluations/testing may be deemed necessary by the investigator and for reasons related to subject safety.

5.1 Administrative Procedures

5.1.1 Informed Consent

The investigator or qualified designee must obtain documented consent from each potential subject or each subject's legally acceptable representative prior to participating in a clinical trial or Future Biomedical Research. If there are changes to the subject's status during the trial (e.g., health or age of majority requirements), the investigator or qualified designee must ensure the appropriate consent is in place.

5.1.1.1 General Informed Consent

Consent must be documented by the subject's dated signature or by the subject's legally acceptable representative's dated signature on a consent form along with the dated signature of the person conducting the consent discussion.

A copy of the signed and dated consent form should be given to the subject before participation in the trial.

The initial informed consent form, any subsequent revised written informed consent form and any written information provided to the subject must receive the IRB/ERC's approval/favorable opinion in advance of use. The subject or his/her legally acceptable representative should be informed in a timely manner if new information becomes available that may be relevant to the subject's willingness to continue participation in the trial. The communication of this information will be provided and documented via a revised consent form or addendum to the original consent form that captures the subject's dated signature or by the subject's legally acceptable representative's dated signature.

Specifics about a trial and the trial population will be added to the consent form template at the protocol level.

The informed consent will adhere to IRB/ERC requirements, applicable laws and regulations.

5.1.1.2 Consent and Collection of Specimens for Future Biomedical Research

The investigator or qualified designee will explain the Future Biomedical Research consent to the subject, answer all of his/her questions, and obtain written informed consent before performing any procedure related to the Future Biomedical Research sub-trial. A copy of the informed consent will be given to the subject.

5.1.2 Inclusion/Exclusion Criteria

All inclusion and exclusion criteria will be reviewed by the Investigator or qualified designee to ensure that the subject qualifies for the trial.

5.1.3 Medical History

A medical history will be obtained by the investigator or qualified designee. Medical history will include all active conditions, and any condition diagnosed within the prior 10 years that are considered to be clinically significant by the Investigator. Any autoimmune disorders, regardless of onset date, should be recorded.

Details regarding subject’s Melanoma diagnosis and status at baseline must be thoroughly evaluated by the investigator or qualified designee including:date of initial diagnosis, stage at diagnosis, primary tumor location and histology subtype, primary and sentinel lymph node biopsies and results, etc. Refer to Entry Criteria to ensure subject’s disease status meets the relevant inclusion and exclusion criteria for study entry.

5.1.4 Concomitant Medications

5.1.4.1 Prior Medications

The investigator or qualified designee will review prior medication use, including any protocol-specified washout requirement, and record prior medication taken by the subject within 30 days prior to the first dose of trial treatment.

5.1.4.2 Concomitant Medications

The investigator or qualified designee will record medication, if any, taken by the participant during the study. Concomitant medications will be recorded for 30 days after the last dose of study treatment.

5.1.5 Study Intervention Administration

Lenvatinib may be administered at home except on C1D1, C2D1 during neoadjuvant phase; on these days, lenvatinib will be taken in the clinic.

Administration of pembrolizumab will be witnessed by the investigator and/or study staff.

5.1.5.1 Timing of Dose Administration

5.1.5.1.1 Lenvatinib

Lenvatinib20mg once daily will be taken orally with water (with or without food) at approximately the same time each day in 21-day cycles. However, on C1D1, and C2D1, lenvatinib will be administered in the clinic 0-4 hours after completion of pembrolizumab administration. Participants should not take their study medication on C2D1 before their appointment.

If a lenvatinib dose is missed and cannot be taken within 12 hours, then that dose should be

skipped, and the next dose should be taken at the usual time of administration.

5.1.5.1.2 Pembrolizumab

Pembrolizumab will be administered as a 30-minute IV infusion on Day 1 of each 21-day cycle. Sites should make every effort to target infusion timing to be as close to 30 minutes as possible. However, given the variability of infusion pumps from site to site, a window of−5 minutes and +10 minutes is permitted (ie, infusion time is 30 minutes [−5 min/+10 min]).

After Cycle 1 Day 1, pembrolizumab may be administered up to 3 days before or after the scheduled Day 1 of each subsequent cycle due to administrative reasons.

5.1.5.2 Compliance

Interruptions from the protocol specified treatment ⩾ 28 days (lenvatinib) or ⩾ 6 weeks (pembrolizumab) need to be discussed for continuation of study treatment.

Lenvatinib compliance will be calculated based on the drug accountability documented by the site staff and monitored by the designee. The objective is 100% compliance and investigators and their staff should evaluate compliance at each visit and take appropriate steps to optimize compliance.

Administration of pembrolizumab will be witnessed by the investigator and/or qualified designee. The total volume of study intervention infused will be compared with the total volume prepared to determine compliance with each dose administered.

5.1.6 Discontinuation and Withdrawal

5.1.6.1 Discontinuation and Withdrawal from the main study

Participants who discontinue study intervention prior to completion of the treatment period should be encouraged to continue to be followed for all remaining study visits.

When a participant withdraws from participation in the study, all applicable activities scheduled for the discontinuation visit (EOT visit) should be performed (at the time of withdrawal). Any AEs that are present at the time of withdrawal should be followed in accordance with the safety requirements outlined in the safety reviewing section. If discontinuation occurs approximately 30 days after the last dose of study treatment, the 30-day Safety Follow-up Visit is not required. In this situation, all procedures required at the 30-day Safety Visit and EOT are performed once and entered into the EOT visit only.

5.1.6.1 Withdrawal From Future Biomedical Research

Subjects may withdraw their consent for Future Biomedical Research and have their specimens and all derivatives destroyed. Subjects may withdraw consent at any time by contacting the principal investigator for the main trial. Any analyses in progress at the time of request for

destruction or already performed prior to the request being received by the investigator will continue to be used as part of the overall research trial data and results. No new analyses would be generated after the request is received.

5.1.7 Demography

Participant demography information will be collected at the Screening visit. Demography information includes date of birth (or age), sex, race/ethnicity.

5.2 Clinical Procedures/Assessments

5.2.1 Adverse Event Monitoring

The investigator or qualified designee will assess each participant to evaluate for potential new or worsening AEs as specified in the Trial Flow Chart and more frequently if clinically indicated. Adverse experiences will be graded and recorded throughout the study and during the follow-up period according to NCI CTCAE Version 5.0(see Appendix 2). Toxicities will be characterized in terms regarding seriousness, causality, toxicity grading, and action taken with regard to trial treatment.

5.2.2 12-Lead Electrocardiogram

A 12-lead electrocardiogram (ECG) will be performed at Screening, using local standard procedures at the time points specified in schedule of event. Clinically significant abnormal findings should be recorded as medical history.

Additional assessments will be performed prior to definitive surgery and at EOT visit, at Safety Follow Up visits and assessments may be performed as clinically necessary.

5.2.3 Echocardiography or Multigated Acquisition Scan

An ECHO or MUGA scan will be required at screening for subjects. The assessment method will be at the investigator 's discretion.

Additional assessments will be performed prior to definitive surgery and at EOT visit, at Safety Follow Up visits and assessments may be performed as clinically necessary.

5.2.4 Physical Examination

5.2.4.1 Full Physical Examination

The investigator or qualified clinical designee will perform a complete physical examination during the screening period. Clinically significant abnormal findings should be recorded as medical history. Full physical examinations should be performed at the discretion of the physician according to the subject's signs and symptoms during study.

5.2.4.2 Directed Physical Examination

The investigator or qualified clinical designee will perform directed physical examinations to assess subject's status according to the time points as specified in schedule of event as needed according to subject's signs and symptoms. New clinically significant abnormal findings should be recorded as AEs.

5.2.5 Vital Signs

The Investigator or qualified clinical designee will take vital signs at screening, prior to the administration of each dose of trial treatment, prior to definitive surgery, at EOT visit, at the Safety Follow Up visits as specified in schedule of event. Vital signs should include temperature, pulse, respiratory rate, weight and blood pressure. Height will be measured at screening only. Vital signs should be taken prior to treatment administration.

5.2.6 Eastern Cooperative Oncology Group Performance Status

The investigator or qualified clinical designee will assess ECOG performance status (schedule of event – ECOG Performance Status Scale) at screening, prior to dosing on Day 1 of each treatment cycle, at EOT visit and Safety Follow Up visits as specified in schedule of event.

5.2.7 Tumor Tissue Biopsy and Sample Collection

Subjects are required to have tumor biopsy performed during the Screening Period

During screening, FFPE tumor tissue samples or slides obtained at subject's initial diagnosis maybe submitted for confirmation of subject's melanoma status, only if a new biopsy cannot be obtained due to site inaccessibility or a medical contraindication.

An optional biopsy should be obtained on Day 22 of neoadjuvant phase.

Tumor tissue samples will also be collected at definitive surgery for subjects.

Tumor biopsy will be performed only if the consent for Future Biomedical Research has been

obtained.

5.2.8 Optional Blood Sample Collection for Future Biomedical Research

20ml of venous peripheral blood will be collected in sodium heparin vacutainersat baseline, 8ml of venous peripheral blood will be collected on neoadjuvant phase day 8, day 22, which complete the first week and third week neoadjuvant treatment respectively.

20 ml venous peripheral bloodwill be collected on surgery, then 8ml blood be collected on day 22 after completed surgery 3 weeks. If patients happen PD within 1 year after surgery, 20 ml venous peripheral blood will be collected at PD time point. If no PD within 1 year, 8ml blood will be collected at 1 year after surgery.

Blood sample will be used for safety evaluation and potential biomarker reserch. Blood Sample Collection will be performed only if the protocol ICF and additional Future Biomedical Research ICF all have been obtained.

5.2.9 Imaging Disease Assessment

In general, imaging should include the chest, abdomen, and pelvis. Tumor imaging of the chest is to be acquired by computed tomography (CT). For the abdomen and pelvis, contrast-enhanced magnetic resonance imaging (MRI) may be used when CT with iodinated contrast is contraindicated. Contrast-enhanced MRI is the strongly preferred modality for imaging the brain. The same imaging technique regarding modality and the use of contrast should be used in a participant throughout the study to optimize the reproducibility of the assessment of existing and new tumor burden and improve the accuracy of the assessment of response or progression based on imaging.

5.2.9.1 Initial Tumor Imaging

Initial tumor imaging at Screening must be performed within 28 days prior to the date of treatment start.

Tumor imaging performed as part of routine clinical management is acceptable for use as screening tumor imaging if it is of diagnostic quality and performed within 28 days prior to the date of treatment start. Brain imaging must be performed at screening. If MRI is medically contraindicated, CT is an acceptable alternative.

Tumor imaging at baseline includes the following:

- CT (preferred) or MRI of the abdomen and pelvis, must include IV contrast
- CT of the chest
- CT of the neck if disease locates in oral cavity and pharyngeal region

- Brain MRI (preferred) or CT of the brain, must include IV contrast
- PET/CT

RECIST 1.1 is encouraged for the measurement of disease. However, given that early stage mucosal diseases are generally scattered in distribution and small in size, it is sometimes difficult to detect them with regular CT or MRI. Therefore, in such case PET/CT should be taken together with endoscopy when measuring the size of the disease.

5.2.9.2 Tumor Imaging during the Study

The first on-study imaging assessment should be performed at completion of neoadjuvant therapy prior to surgery for assessment of tumor response and eligibility for surgery. A second imaging assessment, which includes only the sites where disease used to locate, should be performed after surgery but prior to initiation of adjuvant therapy. Subsequent tumor imaging should be performed after surgery at a frequency of Q12W (±7 days) or more frequently if clinically indicated. Imaging timing can be adjusted for delays in cycle starts. Imaging should continue to be performed until recurrent or metastatic disease is identified by the Investigator, the start of new anticancer treatment, withdrawal of consent, or death, whichever occurs first.

Following initial brain imaging, subsequent brain imaging will be performed once a year (±1 month) from the end of surgery, or sooner if clinically indicated, until Year 2.

A second PET/CT will be performed after surgery but prior to initiation of adjuvant therapy.

5.2.9.3 End-of-Treatment and Follow-up Imaging

For participants who discontinue study treatment, tumor imaging should be performed at the time of treatment discontinuation (±4-week window). If previous imaging was obtained within 4 weeks prior to the date of discontinuation, then imaging at treatment discontinuation is not mandatory. For participants who discontinue study treatment due to documented disease progression or recurrence, this is the final required tumor imaging.

For participants who discontinue for reasons other than disease progression or recurrence, imaging should be performed at regular frequency as stated above.

5.2.10 Endoscopy assessment

If applicable, endoscopy, including but not limit to colonoscopy, proctoscopy, gastroscopy, colposcopy and nasopharyngoscopy, should be performed at baseline and after neoadjuvant therapy for assessment of tumor response, thereafter, it should be performed at a frequency of every 6 month after surgery.

5.2.11 Definitive surgery

Approximately 1-3 weeks following completion of the Neoadjuvant Treatment Phase or Early Discontinuation, subjects will undergo definitive surgery. Details regarding date of surgery, type of surgery, tumor resectability etc. will be collected. Detailed pathological assessment of pCR, major pathologic response and surgical margins will be performed by the pathologist on all the tissues removed during the surgery and recorded. Complete pathologic response was defined as the absence of viable tumor based on hematoxylin and eosin (H&E) staining. A major pathologic response was defined as less than 10% of viable tumor.

5.2.12 Surgical outcomes

Surgical outcomes, as outlined below should be recorded till complete recovery from the surgery.

i. Episodes of infection requiring antibiotics and/or wound drainage.

j. Duration of time from surgery end (time to recovery) to removal of wound drain because of ceased / minimal drainage

k. Episodes of seroma formation at the wound site requiring any intervention and volume of seroma drainage

l. Episodes of bleeding from the wound requiring a return to theatre and / or blood transfusion post-surgery.

m. Number of episodes (and patient number) of wound separation requiring any intervention

n. Incidence of lymphoedema or worsening lymphedema

o. An assessment of 'operability' at the time of surgery compared to the baseline evaluation.

5.3 Laboratory Procedures/Assessments

The investigator or medically qualified designee must review the laboratory report。 The laboratory reports must be filed with the source documents. Clinically significant abnormal laboratory findings are those which are not associated with the underlying disease, unless judged by the investigator to be more severe than expected for the participant's condition.

If laboratory values from nonprotocol specified laboratory assessments require a change in study participant management or are considered clinically significant by the investigator (eg, SAE or AE or dose modification), then the results must be collected.

For any laboratory tests with values considered clinically significantly abnormal during participation in the study or within 30 days after the last dose of study intervention, every attempt should be made to perform repeat assessments until the values return to normal or baseline or if a

new baseline is established as determined by the investigator.

Details regarding specific laboratory procedures/assessments to be performed in this trial are provided below.

5.3.1 Laboratory Safety Evaluations (Hematology, Chemistry, and Urinalysis)

Table Laboratory Tests

Hematology	Chemistry	Urinalysis	Other
Hematocrit	Albumin	Blood	Serum β-human Chorionic Gonadotropin (β-hCG)[a]
Hemoglobin	Alkaline Phosphatase	Glucose	PT (INR)[b]
Platelet Count	Alanine Aminotransferase (ALT)	Protein	Total Thriiodothyronine (T3)[c]
White Blood Cell -WBC (total and differential)	Aspartate Aminotransferase (AST)	Specific Gravity	Free Thyroxine (FT4)
Red Blood Cell Count	Carbon Dioxide (CO_2 or Bicarbonate)	Microscopic exam, if abnormal results are noted	Thyroid Stimulating Hormone (TSH)
Absolute Neutrophil Count	Calcium	Urine Pregnancy Test[a]	
Absolute Lymphocyte Count	Chloride		
	Creatinine or Creatinine clearance (CrCl)		
	Glucose		
	Lactate Dehydrogenase (LDH)		
	Phosphorus		
	Potassium		
	Sodium		
	Total Bilirubin		
	Direct Bilirubin, if Total Bilirubin is elevated above the upper limit of normal		
	Total Protein		

Blood Urea Nitrogen
or Urea[g]
Uric Acid

a Perform on women of childbearing potential only. Urine pregnancy test is preferred. If the urine test is positive or cannot be confirmed as negative, a serum pregnancy test will be required. The serum or urine pregnancy test should be performed within 1 week prior to first dose of trial treatment

b Coagulation factors (PT/INR and aPTT/PTT) should be tested as part of the screening procedures. Any subject receiving anticoagulant therapy should have coagulation factors monitored closely throughout the trial.

c Total T3 is preferred; if not available free T3 may be tested.

Laboratory tests for screening should be performed within 1 week of treatment initiation. After the Neoadjuvant Treatment Phase, Treatment 1 Cycle 1, pre-dose laboratory procedures can be conducted up to 72 hours prior to dosing. Results must be reviewed by the investigator or qualified designee and found to be acceptable prior to each dose of trial treatment.

For women of reproductive potential, a serum or urine pregnancy test should be performed within 1 week prior to first dose of trial treatment. If urine pregnancy results are positive or cannot be confirmed as negative, a serum pregnancy test performed by the local study site laboratory will be required.

Participants with >1+ proteinuria on urine dipstick during screening will undergo 24-hour urine collection for quantitative assessment of proteinuria. Participants with urine protein ≥ 1 g/24-hour will not be eligible.

Once participants are enrolled, urine dipstick testing for participants with proteinuria ≥2+should be performed on Day 22 (or more frequently as clinically indicated) until the results have been 1+ or negative for 2 consecutive treatment cycles. If a new event of proteinuria ≥2+ occurs, the participant must resume the Day 22 urine dipstick testing for evaluation of proteinuria until results are 1+ or negative for 2 consecutive treatment cycles.

For participants with proteinuria ≥2+, please refer dose modification section

5.4 Visit Requirements

5.4.1 Screening

Approximately 28 days prior to treatment start, potential subjects will be evaluated to determine that they fulfill the entry requirements.

Results of a test performed prior to the subject signing consent as part of routine clinical management are acceptable in lieu of a screening test if performed within the specified time frame.

Screening procedures are to be completed within 28 days prior to treatment start except for the

following:

- Laboratory tests and ECOG performance status are to be performed within 1 weekdays prior to treatment initiation.
- For women of reproductive potential, a urine and/or serum pregnancy test will be performed within 1 week prior to receiving the first dose of study medication.

Subjects may be rescreened twice after initially failing to meet the inclusion/exclusion criteria. Results from assessments performed during the initial screening period are acceptable in lieu of a repeat screening test if performed within the specified time frame and the inclusion/exclusion criteria is met.

5.4.2 Treatment Cycles

Visit timing requirements during the treatment period are as follows:

- Assessments/procedures should be performed on Day 1 for each cycle, unless otherwise specified.
- Treatment cycles are 3 weeks (21 days).
- The window for each visit is ± 3 days, unless otherwise noted.

5.4.2.1 Neoadjuvant Treatment Phase

Visit timing requirements during the Neoadjuvant Treatment Phase are as follows:

- Assessments/procedures should be performed on Day 1 for each cycle , unless otherwise specified in the flow chart.
- Treatment cycles are 3 weeks (21 days).
- If the treatment is delayed, all procedures should be performed based on the new dosing schedule.

5.4.2.2 Adjuvant Treatment Phase

The Adjuvant Treatment Phase is expected to start within 6 weeks after definitive surgery . If the subject is found to have disease progression prior to surgery, they will not proceed to the adjuvant treatment phase.

5.4.3 Definitive Surgery

Definitive surgery will be performed approximately 1-3 weeks following discontinuation or completion of study treatment in the Neoadjuvant Treatment Phase.

5.4.4 End of treatment Visit

The End of treatment Visit should be conducted if subject complete designated treatment or discontinues all protocol -specified treatment after Treatment initiation. If the End of treatment Visit occurs 30 days from the last dose of study treatment, at the time of the mandatory Safety Follow Up Visit, procedures listed in the End of treatment Visit should be performed.

5.4.5 Safety Follow-up Visits

Mandatory Safety Follow-up Visits should be conducted approximately 30 days (±3 days) following end of the last dosing. If an Early Discontinuation visit occurs, then every attempt should be made to perform a 30-Day Safety Follow Up visit (30 days ± 3 days).

All AEs that occur prior to the Safety Follow-up Visit should be recorded. Subjects with an AE Grade >1 will be followed until resolution of the AE to Grade 0-1.

5.4.6 Long Term Follow-Up for Disease Status and Survival

The schedule for in-office Long Term Follow-Up visits is as follows: for the first 2 years following adjuvant treatment, assessments will occur every 12 weeks (± 7days); then every 6 months (± 7 days) in years later for assessment of recurrent or metastatic disease. Additional tests/investigations/imaging assessments for recurrent or metastatic disease (e.g. bone/liver scan) will be at the discretion of subject's treating physician.

If a subject does not return for protocol-specified visit assessments in the Long Term Follow Up period, all attempts should be made to contact the subject via telephone for Survival Status every 6 months (± 1 month). If a subject ends treatment in the Neoadjuvant or Adjuvant Treatment Phases for disease progression or recurrence, the subject should be followed every 6 months ± 1 month via telephone for Survival Status.

The subject will be contacted by to assess for disease and survival status until disease recurrence or death, withdrawal of consent for the trial, whichever comes first. Date of disease recurrence should be collected. For a subject who dies during the follow up period, date and cause of death should be collected.

5.4.7 Discontinued Subjects Continuing to be Monitored in the Trial

Subjects who discontinue treatment will be followed up by telephone every 6 months (±1 month) for OS until consent withdrawal from trial, becoming lost to follow-up, death or end of the study, whichever comes first.

Date of disease recurrence or metastatic progression should be recorded in the appropriate eCRF. For subjects who die during the follow up period, the date and cause of death should be recorded.

5.4.8 Study Calendar

**Trial Period**	**Screening Phase**	**Neoadjuvant Treatment Phase[a]**			**Definitive Surgery**	**Post-surgery assesment**		**Adjuvant Treatment Phase[a]**	**EOT visit[b]**	**Safety Follow-up[b,c]**	**Long Term Follow-up[d]**
											~2yr
Visit timing/ Cycle day	-28 to -1d	Day1	Day 8	Day 22	1 to 3w	0 to 6w	1 year	C1D1-C15D1	At DC	30 days after last Dose	Q12w
Scheduling Window	-28 to -1d	\		±3d	1 to 3w	0 to 6w		±3d		±3d	±1m
**Informed Consent**	**X**										
**Inclusion/Exclusion Criteria**	**X**										
**Demographics**	**X**										
**Medical History**	**X**										
**Concomitant Medication**	**X**	**X**		**X**	**X**	**X**	**X**	**X**	**X**	**X**	
**Pembrolizumab**		**X**		**X**				**X**			
**Lenvatinib[e]**		**X**		**X**							
**Survival Status**		**X**		**X**	**X**	**X**		**X**	**X**	**X**	**X**
**Review Adverse Eventsf**	**X**	**X**		**X**	**X**	**X**	**X**	**X**	**X**	**X**	**X**
**ECG and LVEF**	**X**				**X**		**X**		**X**	**X**	
**PE/Vitals/ECOG**	**X**	**X**		**X**	**X**	**X**	**X**	**X**	**X**	**X**	
**Tumor**	**X**			**X**	**X**						

**biopsy**											
**blood[g]**	**X**		**X**	**X**	**X**	**X**	**X**		**X[l]**		
**Pregnancy Test –Urine or Serum -HCG**	**X**										
**HIV, HBV, HCV**	**X**				**X**						
**Laboratory test[h]**	**X**	**X**		**X**	**X**	**X**	**X**	**X**	**X**	**X**	
**Pathology assessment**						**X**					
**Surgical outcomes**						**X**					
**Tumor assessment –chest, abdomen and pelvis (CT/MRI)[i]**	**X**				**X**	**X**	**X**	**X**	**X**		**X**
**PET/CT**	**X**					**X**					
**Endoscopy[j]**	**X**				**X**			**X**	**X**		**X**
**Brain MRI[k]**	**X**						**X**	**X**			**X**

a. In general, assessments/procedures are performed on Day 1 of each cycle prior to dosing of any study treatment unless otherwise specified. Each treatment cycle is 3 weeks (21 days). If the treatment is delayed, all procedures should be performed based on the new dosing schedule. Adjuvant treatment is expected to start no later than 4 weeks post definitive surgery.
b. If EOT visit occurs ~30 days from last dose of study treatment, a 30-day safety follow-up visit is not required. In this situation, all procedures required at the 30-day Safety Visit and EOT are performed once and entered the EOT visit only. End of treatment will be defined as the date when the participant discontinues all trial treatments.
c. The Safety Follow Up visit should be performed 30 days after the lost dose of study

treatment. If an Early Discontinuation occurs, then every attempt should be made to perform a 30-Day Safety Follow Up visit (30 days ± 3 days).

d. For participant who discontinue adjuvant therapy earlier for reasons other than disease recurrence, follow-up visits to monitor disease status until recurrence or initiation of a new anticancer therapy. Participants who has not received definite surgery or adjuvant therapy with disease recurrence will be followed for survival only.

e. Lenvatinib is administrated once daily throughout the neoadjuvant phase at a fixed dose of 20mg.

f. Report non-serious AEs occurring within 30 days after the last dose of study intervention. Report SAEs occurring within 90 days after the last dose of study intervention, or 30 days after the last dose of study intervention if a new anticancer therapy is initiated, whichever is earlier.

g. 20ml of venous peripheral blood will be collected in sodium heparin vacutainersat baseline, 8ml of venous peripheral blood will be collected on neoadjuvant phase day 8, day 22, which complete the first week and third week neoadjuvant treatment respectively.

   20 ml venous peripheral bloodwill be collected on surgery, then 8ml blood be collected on day 22 after completed surgery 3 weeks. If patients happen PD within 1 year after surgery, 20 ml venous peripheral blood will be collected at PD time point. If no PD within 1 year, 8ml blood will be collected at 1 year after surgery.

   Blood sample will be used for safety evaluation and potential biomarker research. Blood Sample Collection will be performed only if the protocol ICF and additional Future Biomedical Research ICF all have been obtained.

h. Laboratory test consist of hematology, biochemistry, urinalysis, coagulation and thyroid function and should be performed within 1 week prior to treatment initiation during the screening phase. Thereafter, it can be conducted up to 72 hours prior to each dosing.

i. Prior scans performed within the screening period but before signing informed consent may be used if consistent with protocol requirements. Post-surgery scan only cover the sites where diseases used to locate. All imaging visits have a scheduling window of ±7d. Imaging to be performed Q12W or sooner if clinically indicated from the date of start of treatment until disease recurrence or the follow-up reaches 2 years. Recurrence will be verified prior to DC of study treatment. For participants who DC for reasons other than recurrence, imaging should be performed Q12W until recurrence. Imaging at EOT is not required if the previous tumor imaging assessment was within 4 weeks

j. Endoscopy, including but not limit to colonoscopy, proctoscopy, gastroscopy, colposcopy and nasopharyngoscopy, should be performed at baseline, after completing neoadjuvant therapy and at a frequency of every 6 month post surgery, if applicable to the primary tumor site.

k. Brain MRI should be performed at baseline and every year thereafter.

l. If patients happen PD within 1 year after surgery, blood sample will be collected at PD time point.

6. Assessing and Recording Adverse Event

The definitions of an AE or SAE, as well as the method of recording, evaluating, and assessing causality of AE and SAE and the procedures for completing and transmitting AE, SAE, and other reportable safety event reports can be found in Appendix 3.

Adverse events, SAEs, and other reportable safety events will be reported by the participant (or, when appropriate, by a caregiver, surrogate, or the participant's legally authorized representative).

The investigator and any designees are responsible for detecting, documenting, and reporting events that meet the definition of an AE or SAE as well as other reportable safety events. Investigators remain responsible for following up AEs, SAEs, and other reportable safety events for outcome.

The investigator, who is a qualified physician, will assess events that meet the definition of an AE or SAE as well as other reportable safety events with respect to seriousness, intensity/toxicity and causality.

6.2.1 Time Period and Frequency for Collecting AE, SAE, and Other Reportable Safety Event Information

All AEs, SAEs, and other reportable safety events that occur after the consent form is signed but before intervention allocation/randomization must be reported by the investigator if the participant is receiving placebo run-in or other run-in treatment, if the event cause the participant to be excluded from the study, or is the result of a protocol-specified intervention, including but not limited to washout or discontinuation of usual therapy, diet, or a procedure.

- All AEs from the time of intervention allocation/randomization through 30 days following cessation of study intervention must be reported by the investigator.
- All AEs meeting serious criteria, from the time of intervention allocation/randomization through 90 days following cessation of study intervention or 30 days following cessation of study intervention if the participant initiates new anticancer therapy, whichever is earlier, must be reported by the investigator.

- All pregnancies and exposure during breastfeeding, from the time of intervention allocation/randomization through 120 days following cessation of study intervention, or 30 days following cessation of study intervention if the participant initiates new anticancer therapy must be reported by the investigator.
- Additionally, any SAE brought to the attention of an investigator at any time outside of the time period specified above must be reported immediately to Merck if the event is considered drug-related.

Investigators are not obligated to actively seek AEs or SAEs or other reportable safety events in former study participants. However, if the investigator learns of any SAE, including a death, at any time after a participant has been discharged from the study, and he/she considers the event to be reasonably related to the study intervention or study participation, the investigator must promptly notify Merck.

All initial and follow-up AEs, SAEs, and other reportable safety events will be recorded and reported to Merck within the time frames as indicated in Table 7.

Table 7 Reporting Time Periods and Time Frames for Adverse Events and Other Reportable Safety Events

**Type of Event**	**<u>Reporting Time Period:</u>** **Consent to Randomization/ Allocation**	**<u>Reporting Time Period:</u>** **Randomization/ Allocation through Protocol-specified Follow-up Period**	**<u>Reporting Time Period:</u>** **After the Protocol-specified Follow-up Period**	**Time Frame to Report Event and Follow-up Information to Merck:**
Serious Adverse Event (SAE) including Cancer and Overdose	Report if:<br>- due to protocol-specified intervention<br>- causes exclusion<br>- participant is receiving placebo run-in or other run-in treatment	Report all	Report if:<br>- drug/vaccine related.<br>(Follow ongoing to outcome)	Within 24 hours of learning of event
Pregnancy/Lactation Exposure	Report if:<br>- due to intervention	Report all	Previously reported – Follow to completion/termination;	Within 24 hours of learning

Type of Event	Reporting Time Period: Consent to Randomization/ Allocation	Reporting Time Period: Randomization/ Allocation through Protocol-specified Follow-up Period	Reporting Time Period: After the Protocol-specified Follow-up Period	Time Frame to Report Event and Follow-up Information to Merck:
	- causes exclusion		report outcome	of event
Event of Clinical Interest (require regulatory reporting)	Report if:<br>- due to intervention<br>- causes exclusion	Report<br>- potential drug-induced liver injury (DILI)<br>- require regulatory reporting	Not required	Within 24 hours of learning of event

6.2.2 Method of Detecting AEs, SAEs, and Other Reportable Safety Events

Care will be taken not to introduce bias when detecting AEs and/or SAEs and other reportable safety events. Open-ended and nonleading verbal questioning of the participant is the preferred method to inquire about AE occurrence.

6.2.3 Follow-up of AE, SAE, and Other Reportable Safety Event Information

After the initial AE/SAE report, the investigator is required to proactively follow each participant at subsequent visits/contacts. All AEs, SAEs, and other reportable safety events including pregnancy and exposure during breastfeeding, events of clinical interest (ECIs), cancer, and overdose will be followed until resolution, stabilization, until the event is otherwise explained, or the participant is lost to follow-up.. In addition, the investigator will make every attempt to follow all nonserious AEs that occur in randomized participants for outcome. Further information on follow-up procedures is given in Appendix 3.

6.2.4 Sponsor Responsibility for Reporting Adverse Events

All Adverse Events will be reported to regulatory authorities, IRB/IECs and investigators in

accordance with all applicable country specific regulatory requirements, global laws and regulations.

6.2.5 Pregnancy and Exposure During Breastfeeding

Although pregnancy and infant exposure during breastfeeding are not considered AEs, any pregnancy or infant exposure during breastfeeding in a participant (spontaneously reported to the investigator or their designee) that occurs during the study are reportable to Merck.

All reported pregnancies must be followed to the completion/termination of the pregnancy. Pregnancy outcomes of spontaneous abortion, missed abortion, benign hydatidiform mole, blighted ovum, fetal death, intrauterine death, miscarriage, and stillbirth must be reported as serious events (Important Medical Events). If the pregnancy continues to term, the outcome (health of infant) must also be reported.

6.2.6 Events of Clinical Interest (ECIs)

Selected nonserious and SAEs are also known as ECIs and must be reported to MSD.

Events of clinical interest for this study include:

1. An overdose of pembrolizumab that is not associated with clinical symptoms or abnormal laboratory results. For purposes of this study, an overdose of pembrolizumab will be defined as any dose of 1,000 mg or greater (≥5 times the indicated dose). No specific information is available on the treatment of overdose of pembrolizumab. In the event of overdose, the participant should be observed closely for signs of toxicity. Appropriate supportive treatment should be provided if clinically indicated. If an adverse event(s) is associated with ("results from") the overdose of a MSD product, the adverse event(s) is reported as a serious adverse event, even if no other seriousness criteria are met.

2. An elevated AST or ALT lab value that is greater than or equal to 3X the upper limit of normal and an elevated total bilirubin lab value that is greater than or equal to 2X the upper limit of normal and, at the same time, an alkaline phosphatase lab value that is less than 2X the upper limit of normal, as determined by way of protocol-specified laboratory testing or unscheduled laboratory testing.*

*Note: These criteria are based upon available regulatory guidance documents. The purpose of the criteria is to specify a threshold of abnormal hepatic tests that may require an additional evaluation for an underlying etiology. MSDMSD

7. STATISTICAL ANALYSIS PLAN

7.1 Variables/Time Points of Interest

- Pathological response rate
- 1-year relapse-free survival (RFS) rate
- Overall survival (OS)
- Surgical outcomes
- Safety

7.2 Statistical Methods

The population to be included in the Intention to Treat (ITT) analysis will be all patients who receive at least 1 treatment with Pembrolizumab. Patients who received at least 1 cycle of Pembrolizumab but did not undergo surgery were included in the toxicity analyses.

RFS was measured from the date of surgery until disease recurrence or death. OS was measured from the date of enrollment to date of death from any cause and was censored at the date of the last follow-up visit for patients who were alive at that time. Time-to-event endpoints (OS, RFS) were analyzed using the Kaplan–Meier (KM) method.

7.3 Power/Sample Size:

Sample size estimation was based on pCR rate. Although observations showed that 30% of patients achieved a complete pathologic response with neoadjuvant pembrolizumab monotherapy in resectable melanoma (mainly cutaneous), no direct data provide pCR rate in mucosal subtype. According to results from KEYNOTE-151 and previous data from shoushtari (Cancer, 122, 21, (3263-3266), (2016)), ORR rate of metastatic mucosal melanoma was form 13% to 23%, thus we assumed that the pCR rate would be 30% after neoadjuvant pembrolizumab plus levantinib combination in mucosal melanoma. Target pCR rate is 30% and 23 patients was needed with confidence interval width of 40%.

Sample size is 26 subjects considering 10% dropout rate.

7.4 Future Collateral Biomarker Research:

Tissue being collected in the study will be kept for a future separated biomarker analysis study of neoadjuvant treatment in mucosal melanoma.

APPENDICES

Appendix 1: ECOG Performance Status

Grade	Description
0	Normal activity. Fully active, able to carry on all pre-disease performance without restriction.
1	Symptoms, but ambulatory. Restricted in physically strenuous activity, but ambulatory and able to carry out work of a light or sedentary nature (e.g., light housework, office work).
2	In bed <50% of the time. Ambulatory and capable of all self-care, but unable to carry out any work activities. Up and about more than 50% of waking hours.
3	In bed >50% of the time. Capable of only limited self-care, confined to bed or chair more than 50% of waking hours.
4	100% bedridden. Completely disabled. Cannot carry on any self-care. Totally confined to bed or chair.
5	Dead.

* As published in Am. J. Clin. Oncol.: *Oken, M.M., Creech, R.H., Tormey, D.C., Horton, J., Davis, T.E., McFadden, E.T., Carbone, P.P.: Toxicity And Response Criteria Of The Eastern Cooperative Oncology Group. Am J Clin Oncol 5:649-655, 1982.* The Eastern Cooperative Oncology Group, Robert Comis M.D., Group Chair.

Appendix 2: Common Terminology Criteria for Adverse Events V5.0 (CTCAE)

The descriptions and grading scales found in the revised NCI Common Terminology Criteria for Adverse Events (CTCAE) version 5.0 will be utilized for adverse event reporting. (http://ctep.cancer.gov/reporting/ctc.html)

Appendix 3: Adverse Events: Definitions and Procedures for Recording, Evaluating, Follow-up, and Reporting

Definition of AE

AE definition

- An AE is any untoward medical occurrence in a clinical study participant, temporally associated with the use of study intervention, whether or not considered related to the study intervention.
- NOTE: An AE can therefore be any unfavorable and unintended sign (including an abnormal laboratory finding), symptom, or disease (new or exacerbated) temporally associated with the use of a study intervention.
- NOTE: For purposes of AE definition, study intervention (also referred to as MSD product) includes any pharmaceutical product, biological product, vaccine, diagnostic agent, or protocol specified procedure whether investigational or marketed (including placebo, active comparator product, or run-in intervention), manufactured by, licensed by, provided by, or distributed by MSD for human use in this study.

Events meeting the AE definition

- Any abnormal laboratory test results (hematology, clinical chemistry, or urinalysis) or other safety assessments (eg, ECG, radiological scans, vital signs measurements), including those that worsen from baseline, considered clinically significant in the medical and scientific judgment of the investigator.
- Exacerbation of a chronic or intermittent pre-existing condition including either an increase in frequency and/or intensity of the condition.
- New conditions detected or diagnosed after study intervention administration even though it may have been present before the start of the study.
- Signs, symptoms, or the clinical sequelae of a suspected drug-drug interaction.
- Signs, symptoms, or the clinical sequelae of a suspected overdose of either study intervention or a concomitant medication.

- For all reports of overdose (whether accidental or intentional) with an associated AE, the AE term should reflect the clinical symptoms or abnormal test result. An overdose without any associated clinical symptoms or abnormal laboratory results is reported using the terminology “accidental or intentional overdose without adverse effect.”

Events NOT meeting the AE definition

- Medical or surgical procedure (eg, endoscopy, appendectomy): the condition that leads to the procedure is the AE.
- Situations in which an untoward medical occurrence did not occur (social and/or convenience admission to a hospital).
- Anticipated day-to-day fluctuations of pre-existing disease(s) or condition(s) present or detected at the start of the study that do not worsen.
- Surgery planned prior to informed consent to treat a pre-existing condition that has not worsened.

Definition of SAE

If an event is not an AE per definition above, then it cannot be an SAE even if serious conditions are met.

An SAE is defined as any untoward medical occurrence that, at any dose:

a. Results in death

b. Is life-threatening

- The term “life-threatening” in the definition of “serious” refers to an event in which the participant was at risk of death at the time of the event. It does not refer to an event, which hypothetically might have caused death, if it were more severe.

c. Requires inpatient hospitalization or prolongation of existing hospitalization

- Hospitalization is defined as an inpatient admission, regardless of length of stay, even if the hospitalization is a precautionary measure for continued observation. (Note: Hospitalization for an elective procedure to treat a pre-existing condition that has not worsened is not an SAE. A pre-existing condition is a clinical condition that is diagnosed prior to the use of a MSD product and is documented in the participant's medical history.

d. Results in persistent or significant disability/incapacity

- The term disability means a substantial disruption of a person's ability to conduct normal life functions.
- This definition is not intended to include experiences of relatively minor medical significance such as uncomplicated headache, nausea, vomiting, diarrhea, influenza, and accidental trauma (eg, sprained ankle) that may interfere with or prevent everyday life functions but do not constitute a substantial disruption.

e. Is a congenital anomaly/birth defect

- In offspring of participant taking the product regardless of time to diagnosis.

f. Other important medical events

- Medical or scientific judgment should be exercised in deciding whether SAE reporting is appropriate in other situations such as important medical events that may not be immediately life-threatening or result in death or hospitalization but may jeopardize the participant or may require medical or surgical intervention to prevent 1 of the other outcomes listed in the above definition. These events should usually be considered serious.
- Examples of such events include invasive or malignant cancers, intensive treatment in an emergency room or at home for allergic bronchospasm, blood dyscrasias or convulsions that do not result in hospitalization, or development of drug dependency or drug abuse.

Additional Events Reported in the Same Manner as SAE

Additional events that require reporting in the same manner as SAE

In addition to the above criteria, AEs meeting either of the below criteria, although not serious per ICH definition, are reportable to MSD in the same time frame as SAEs to meet certain local requirements. Therefore, these events are considered serious by MSD for collection purposes.

- Is a new cancer (that is not a condition of the study)

- Is associated with an overdose of pembrolizumab

Recording AE and SAE

AE and SAE recording

- When an AE/SAE occurs, it is the responsibility of the investigator to review all documentation (eg, hospital progress notes, laboratory, and diagnostics reports) related to the event.
- The investigator will record all relevant AE/SAE information on the worksheets at each examination.
- There may be instances when copies of medical records for certain cases are requested by the MSD. In this case, all participant identifiers, with the exception of the participant number, will be blinded on the copies of the medical records before submission to the MSD.
- The investigator will attempt to establish a diagnosis of the event based on signs, symptoms, and/or other clinical information. In such cases, the diagnosis (not the individual signs/symptoms) will be documented as the AE/SAE.

Assessment of intensity/toxicity

- An event is defined as "serious" when it meets at least 1 of the predefined outcomes as described in the definition of an SAE, not when it is rated as severe.

1. The investigator will make an assessment of intensity for each AE and SAE (and other reportable safety event) according to the NCI Common Terminology for Adverse Events (CTCAE), version 5. Any AE that changes CTCAE grade over the course of a given episode will have each change of grade recorded on the worksheets.
   - Grade 1: Mild; asymptomatic or mild symptoms; clinical or diagnostic observations only; intervention not indicated.
   - Grade 2: Moderate; minimal, local or noninvasive intervention indicated; limiting age-appropriate instrumental activities of daily living (ADL).
   - Grade 3: Severe or medically significant but not immediately life-threatening; hospitalization or prolongation of hospitalization indicated; disabling; limiting self-care ADL.
   - Grade 4: Life threatening consequences; urgent intervention indicated.
   - Grade 5: Death related to AE.

Assessment of causality

1.Did MSD product cause the AE?

2.The determination of the likelihood that MSD product caused the AE will be provided by an investigator who is a qualified physician. The investigator's signed/dated initials on the source document or worksheet that supports the causality noted on the AE form, ensures that a medically qualified assessment of causality was done. This initialed document must be retained for the required regulatory time frame. The criteria below are intended as reference guidelines to assist the investigator in assessing the likelihood of a relationship between the test product and the AE based upon the available information.

3.The following components are to be used to assess the relationship between MSD's product and the AE; the greater the correlation with the components and their respective elements (in number and/or intensity), the more likely MSD product caused the AE:

- **Exposure:** Is there evidence that the participant was actually exposed to MSD product such as: reliable history, acceptable compliance assessment (pill count, diary, etc.), expected pharmacologic effect, or measurement of drug/metabolite in bodily specimen?

- **Time Course:** Did the AE follow in a reasonable temporal sequence from administration of MSD product? Is the time of onset of the AE compatible with a drug-induced effect (applies to studies with investigational medicinal product)?

- **Likely Cause:** Is the AE not reasonably explained by another etiology such as underlying disease, other drug(s)/vaccine(s), or other host or environmental factors.

- **Dechallenge:** Was MSD product discontinued or dose/exposure/frequency reduced?

- If yes, did the AE resolve or improve?

- If yes, this is a positive dechallenge.

- If no, this is a negative dechallenge.

- (Note: This criterion is not applicable if: (1) the AE resulted in death or permanent disability; (2) the AE resolved/improved despite continuation of the MSD product; (3) the study is a single-dose drug study; or (4) MSD product(s) is/are only used 1 time.)

- **Rechallenge:** Was the participant re-exposed to MSD product in this study?

- If yes, did the AE recur or worsen?
- If yes, this is a positive rechallenge.
- If no, this is a negative rechallenge.

(Note: This criterion is not applicable if: (1) the initial AE resulted in death or permanent disability, or (2) the study is a single-dose drug study; or (3) MSD product(s) is/are used only 1 time.)

NOTE: IF A RECHALLENGE IS PLANNED FOR AN AE THAT WAS SERIOUS AND MAY HAVE BEEN CAUSED BY MSD PRODUCT, OR IF RE-EXPOSURE TO MSD’S PRODUCT POSES ADDITIONAL POTENTIAL SIGNIFICANT RISK TO THE PARTICIPANT THEN THE RECHALLENGE MUST BE APPROVED IN ADVANCE BY THE SPONSOR AS PER DOSE MODIFICATION GUIDELINES IN THE PROTOCOL, AND IF REQUIRED, THE INIRB/IEC.

4.**Consistency with study intervention profile:** Is the clinical/pathological presentation of the AE consistent with previous knowledge regarding MSD product or drug class pharmacology or toxicology?

5.The assessment of relationship will be reported on the case report forms/worksheets by an investigator who is a qualified physician according to his/her best clinical judgment, including consideration of the above elements.

6.Use the following scale of criteria as guidance (not all criteria must be present to be indicative of MSD product relationship).

- Yes, there is a reasonable possibility of MSD product relationship:
- There is evidence of exposure to the MSD product. The temporal sequence of the AE onset relative to the administration of MSD product is reasonable. The AE is more likely explained by MSD product than by another cause.
- No, there is not a reasonable possibility of MSD product relationship:
- Participant did not receive the MSD product OR temporal sequence of the AE onset relative to administration of the MSD product is not reasonable OR the AE is more likely explained by another cause than the MSD product. (Also entered for a participant with overdose without an associated AE.)

7.For each AE/SAE, the investigator must document in the medical notes that he/she has reviewed the AE/SAE and has provided an assessment of causality.

8. There may be situations in which an SAE has occurred and the investigator has minimal information to include in the initial report to the MSD. However, it is very important that the investigator always make an assessment of causality for every event before the initial transmission of the SAE data to MSD.

9. The investigator may change his/her opinion of causality in light of follow-up information and send an SAE follow-up report with the updated causality assessment.

10. The causality assessment is 1 of the criteria used when determining regulatory reporting requirements.

11. For studies in which multiple agents are administered as part of a combination regimen, the investigator may attribute each AE causality to the combination regimen or to a single agent of the combination. In general, causality attribution should be assigned to the combination regimen (ie, to all agents in the regimen). However, causality attribution may be assigned to a single agent if in the investigator's opinion, there is sufficient data to support full attribution of the AE to the single agent.

Follow-up of AE and SAE

- The investigator is obligated to perform or arrange for the conduct of supplemental measurements and/or evaluations as medically indicated to elucidate the nature and/or causality of the AE or SAE as fully as possible. This may include additional laboratory tests or investigations, histopathological examinations, or consultation with other health care professionals.
- The investigator will submit any updated SAE data to MSD Within 24 hours of learning of event.

Reporting of AEs, SAEs, and Other Reportable Safety Events to the MSD

SAE reports and any other relevant safety information are to be forwarded to the Merck Global Safety facsimile number: 86 10 5860 9044.

A copy of all 15 Day Reports and Annual Progress Reports is submitted as required by FDA, European Union (EU), Pharmaceutical and Medical Devices agency (PMDA) or other local regulators. Investigators will cross reference this submission according to local regulations to the Merck Investigational Compound Number (IND, CSA, etc.) at the time of submission. Additionally, investigators will submit a copy of these reports to Merck & Co., Inc. (Attn: Worldwide Product Safety; FAX 215-661-6229) at the time of submission to FDA.

Section/topic	No	CONSORT 2025 checklist item description	Reported on page no.
**Title and abstract**			
Title and structured abstract	1a	Identification as a randomised trial	N/A
	1b	Structured summary of the trial design, methods, results, and conclusions	2
**Open science**			
Trial registration	2	Name of trial registry, identifying number (with URL) and date of registration	14
Protocol and statistical analysis plan	3	Where the trial protocol and statistical analysis plan can be accessed	14
Data sharing	4	Where and how the individual de-identified participant data (including data dictionary), statistical code and any other materials can be accessed	19
Funding and conflicts of interest	5a	Sources of funding and other support (eg, supply of drugs), and role of funders in the design, conduct, analysis and reporting of the trial	23
	5b	Financial and other conflicts of interest of the manuscript authors	24
**Introduction**			
Background and rationale	6	Scientific background and rationale	3
Objectives	7	Specific objectives related to benefits and harms	4
**Methods**			
Patient and public involvement	8	Details of patient or public involvement in the design, conduct and reporting of the trial	14
Trial design	9	Description of trial design including type of trial (eg, parallel group, crossover), allocation ratio, and framework (eg, superiority, equivalence, non-inferiority, exploratory)	14
Changes to trial protocol	10	Important changes to the trial after it commenced including any outcomes or analyses that were not prespecified, with reason	N/A
Trial setting	11	Settings (eg, community, hospital) and locations (eg, countries, sites) where the trial was conducted	14
Eligibility criteria	12a	Eligibility criteria for participants	15
	12b	If applicable, eligibility criteria for sites and for individuals delivering the interventions (eg, surgeons, physiotherapists)	N/A
Intervention and comparator	13	Intervention and comparator with sufficient details to allow replication. If relevant, where additional materials describing the intervention and comparator (eg, intervention manual) can be accessed	N/A
Outcomes	14	Prespecified primary and secondary outcomes, including the specific measurement variable (eg, systolic blood pressure), analysis metric (eg, change from baseline, final value, time to event), method of aggregation (eg, median, proportion), and time point for each outcome	15
Harms	15	How harms were defined and assessed (eg, systematically, non-systematically)	15
Sample size	16a	How sample size was determined, including all assumptions supporting the sample size calculation	14
	16b	Explanation of any interim analyses and stopping guidelines	N/A
Randomisation:			
Sequence generation	17a	Who generated the random allocation sequence and the method used	N/A
	17b	Type of randomisation and details of any restriction (eg, stratification, blocking and block size)	N/A

			Reported on page no.
Allocation concealment mechanism	18	Mechanism used to implement the random allocation sequence (eg, central computer/telephone; sequentially numbered, opaque, sealed containers), describing any steps to conceal the sequence until interventions were assigned	N/A
Implementation	19	Whether the personnel who enrolled and those who assigned participants to the interventions had access to the random allocation sequence	N/A
Blinding	20a	Who was blinded after assignment to interventions (eg, participants, care providers, outcome assessors, data analysts)	N/A
	20b	If blinded, how blinding was achieved and description of the similarity of interventions	N/A
Statistical methods	21a	Statistical methods used to compare groups for primary and secondary outcomes, including harms	19
	21b	Definition of who is included in each analysis (eg, all randomised participants), and in which group	4
	21c	How missing data were handled in the analysis	15
	21d	Methods for any additional analyses (eg, subgroup and sensitivity analyses), distinguishing prespecified from post hoc	N/A
**Results**			
Participant flow, including flow diagram	22a	For each group, the numbers of participants who were randomly assigned, received intended intervention, and were analysed for the primary outcome	N/A
	22b	For each group, losses and exclusions after randomisation, together with reasons	N/A
Recruitment	23a	Dates defining the periods of recruitment and follow-up for outcomes of benefits and harms	14
	23b	If relevant, why the trial ended or was stopped	N/A
Intervention and comparator delivery	24a	Intervention and comparator as they were actually administered (eg, where appropriate, who delivered the intervention/comparator, how participants adhered, whether they were delivered as intended (fidelity))	N/A
	24b	Concomitant care received during the trial for each group	N/A
Baseline data	25	A table showing baseline demographic and clinical characteristics for each group	25
Numbers analysed, outcomes and estimation	26	For each primary and secondary outcome, by group:<br>● the number of participants included in the analysis<br>● the number of participants with available data at the outcome time point<br>● result for each group, and the estimated effect size and its precision (such as 95% confidence interval)<br>● for binary outcomes, presentation of both absolute and relative effect size	4-5
Harms	27	All harms or unintended events in each group	5-6
Ancillary analyses	28	Any other analyses performed, including subgroup and sensitivity analyses, distinguishing pre-specified from post hoc	6
**Discussion**			
Interpretation	29	Interpretation consistent with results, balancing benefits and harms, and considering other relevant evidence	10-13
Limitations	30	Trial limitations, addressing sources of potential bias, imprecision, generalisability, and, if relevant, multiplicity of analyses	13

Citation: Hopewell S, Chan AW, Collins GS, Hróbjartsson A, Moher D, Schulz KF, et al. CONSORT 2025 Statement: updated guideline for reporting randomised trials. BMJ. 2025; 388:e081123.
https://dx.doi.org/10.1136/bmj-2024-081123
© 2025 Hopewell et al. This is an Open Access article distributed under the terms of the Creative Commons Attribution License (https://creativecommons.org/licenses/by/4.0/), which permits unrestricted use, distribution, and reproduction in any medium, provided the original work is properly cited.

*We strongly recommend reading this statement in conjunction with the CONSORT 2025 Explanation and Elaboration and/or the CONSORT 2025 Expanded Checklist for important clarifications on all the items. We also recommend reading relevant CONSORT extensions. See www.consort-spirit.org.
