# Supplementary material for: A phase II peri-operative study of pembrolizumab plus lenvatinib for mucosal melanoma
Source: Nat Commun. 2026 May 16;17:6491. doi: 10.1038/s41467-026-73190-1 (PMC13376369; doi:10.1038/s41467-026-73190-1)
Supplement: Supplementary file 2 — Reporting Summary [file 41467_2026_73190_MOESM2_ESM.pdf]

nature portfolio

Corresponding author(s): Lu Si, Jie Dai

Last updated by author(s): April 14, 2026

Reporting Summary

Nature Portfolio wishes to improve the reproducibility of the work that we publish. This form provides structure for consistency and transparency in reporting. For further information on Nature Portfolio policies, see our Editorial Policies and the Editorial Policy Checklist.

Statistics

For all statistical analyses, confirm that the following items are present in the figure legend, table legend, main text, or Methods section.

n/a	Confirmed	
☐	☒	The exact sample size (*n*) for each experimental group/condition, given as a discrete number and unit of measurement
☐	☒	A statement on whether measurements were taken from distinct samples or whether the same sample was measured repeatedly
☐	☒	The statistical test(s) used AND whether they are one- or two-sided<br>*Only common tests should be described solely by name; describe more complex techniques in the Methods section.*
☐	☒	A description of all covariates tested
☐	☒	A description of any assumptions or corrections, such as tests of normality and adjustment for multiple comparisons
☐	☒	A full description of the statistical parameters including central tendency (e.g. means) or other basic estimates (e.g. regression coefficient) AND variation (e.g. standard deviation) or associated estimates of uncertainty (e.g. confidence intervals)
☐	☒	For null hypothesis testing, the test statistic (e.g. *F*, *t*, *r*) with confidence intervals, effect sizes, degrees of freedom and *P* value noted<br>*Give P values as exact values whenever suitable.*
☒	☐	For Bayesian analysis, information on the choice of priors and Markov chain Monte Carlo settings
☐	☒	For hierarchical and complex designs, identification of the appropriate level for tests and full reporting of outcomes
☐	☒	Estimates of effect sizes (e.g. Cohen's *d*, Pearson's *r*), indicating how they were calculated

Our web collection on statistics for biologists contains articles on many of the points above.

Software and code

Policy information about availability of computer code

Data collection	No software was used for data collection.
Data analysis	All analyses were conducted in R version 4.5.1 and the packages limma 3.64.3, clusterProfiler 4.18.4, org.Hs.eg.db 3.18.0, GSEA4.1.0, fgsea 2.0.4 GseaVis 0.0.5, pheatmap 1.0.13, ComplexHeatmap 2.24.1, GetoptLong 1.0.5, reshape2 1.4.5, ggpubr 0.6.3, ggrepel 0.9.8, ggtext 0.1.2, openxlsx 4.2.8.1, survival 3.8-6, survminer 0.5.2, tidyverse 2.0.0 (which includes ggplot2 4.0.2, dplyr 1.2.1, tidyr 1.3.2, readr 2.2.0, purrr 1.2.2, tibble 3.3.1, stringr 1.6.0, forcats 1.0.1, and lubridate 1.9.5). We used the softwares OptyType 1.3.2, POLYSOLVER1.0, MSisensor 0.2, ABSOLUTE 1.2, PyClone 0.13.1, GISTIC 2.0.22, fastp 0.19.5, samtools 1.1, bowtie2 2.3.5.1, vep 93.7, pvacseq 1.5.9, tcR 2.3.2, immunarch 0.6.5, MixCR 3.0.3, VDJtools 1.2.1, Metascape 3.5.20240901.

For manuscripts utilizing custom algorithms or software that are central to the research but not yet described in published literature, software must be made available to editors and reviewers. We strongly encourage code deposition in a community repository (e.g. GitHub). See the Nature Portfolio guidelines for submitting code & software for further information.

Data

Policy information about availability of data

All manuscripts must include a data availability statement. This statement should provide the following information, where applicable:

- Accession codes, unique identifiers, or web links for publicly available datasets
- A description of any restrictions on data availability
- For clinical datasets or third party data, please ensure that the statement adheres to our policy

The WES data generated in this study have been deposited in the the Genome Sequence Archive under accession code HRA015548 [https://ngdc.cncb.ac.cn/gsa-human/browse/HRA015548]. The GeoMx DSP data generated in this study are deposited in the the Genome Sequence Archive under accession code HRA015543 [https://ngdc.cncb.ac.cn/gsa-human/browse/HRA015543]. The TCR sequencing data generated in this study are deposited in the the Genome Sequence Archive under accession code HRA015547 [https://ngdc.cncb.ac.cn/gsa-human/browse/HRA015547]. The bulk RNA sequencing data of the validation cohort are deposited in the the Genome Sequence Archive under accession code HRA008154 [https://ngdc.cncb.ac.cn/gsa-human/browse/HRA008154]. The data is available under restricted access due to patient privacy, access can be obtained by contacting the corresponding author upon reasonable request. Requests will be reviewed by the institutional ethics committee and will be processed within 3 weeks. Data will be shared to the qualified researchers after a Data Transfer Agreement (DTA) is signed, data access will be granted for a period of 12 months. The study protocol is available in the Supplementary Information file. The remaining data are available within the Article, Supplementary Information. Source data are provided with this paper.

Research involving human participants, their data, or biological material

Policy information about studies with human participants or human data. See also policy information about sex, gender (identity/presentation), and sexual orientation and race, ethnicity and racism.

Reporting on sex and gender	In this study, we enrolled 26 patients, of whom 7 were males and 19 were females. Given the exploratory purpose of the study and the limited sample size, sex-based subgroup analysis was not performed.
Reporting on race, ethnicity, or other socially relevant groupings	Not applicable.
Population characteristics	We recruited 26 patients with resectable mucosal melanoma (aged 49–75 years).
Recruitment	The recruitment for this melanoma-focused clinical trial implemented through the following structured approaches:<br>In-hospital recruitment: With the support of specialized departments including the Melanoma Department, Bone and Soft Tissue Oncology, Gynecologic Oncology Department, Head and Neck Oncology Surgery Department, and Gastrointestinal Oncology Department at PKUCH, our sub-investigators and research nurses will introduce this study to eligible melanoma patients in inpatient wards and outpatient clinics.<br>External recruitment: Eligible melanoma patients will be referred to this trial by physicians from collaborating external hospitals.<br>Advertisement recruitment: Promotional posters and informational brochures will be distributed at medical institutions commonly accessed by melanoma patients. Details of the trial and recruitment procedures will also be published on the official website of Peking University Cancer Hospital. Additionally, recruitment advertisements will be disseminated through diverse media channels—including WeChat (official hospital accounts and dedicated melanoma patient groups), professional medical websites, and specialized medical forums.<br>Patient referral recruitment: Individuals are encouraged to refer potential melanoma candidates for trial screening. Recruitment efforts will also be promoted within dedicated melanoma patient support groups and mutual-aid organizations.<br>Informed consent: Our research team will ensure that all potential participants receive comprehensive, accurate information about the trial's content—with no overstatement of potential benefits—to support their fully voluntary, informed decision-making.<br>It should be noted that this is a single-arm trial focused on melanoma, characterized by a relatively small sample size and no control group was incorporated into the study design. Therefore, the efficacy outcomes reported herein may be subject to selection bias and should be interpreted as preliminary findings.
Ethics oversight	The study was approved by the Peking University Cancer Hospital and Research Institute's ethics committee, and conducted at the Melanoma and Sarcoma Department. All participants provided written informed consent. We confirm that the study design and all clinical procedures complied with all relevant ethical regulations regarding the use of human study participants. The trial was conducted in compliance with the Declaration of Helsinki.

Note that full information on the approval of the study protocol must also be provided in the manuscript.

Field-specific reporting

Please select the one below that is the best fit for your research. If you are not sure, read the appropriate sections before making your selection.

[x] Life sciences [ ] Behavioural & social sciences [ ] Ecological, evolutionary & environmental sciences

For a reference copy of the document with all sections, see nature.com/documents/nr-reporting-summary-flat.pdf

Life sciences study design

All studies must disclose on these points even when the disclosure is negative.

Sample size	The study was designed as an exploratory, estimation-focused phase II trial rather than a confirmatory single-arm trial with a prespecified null pCR benchmark. Based on previously reported ORRs of 13–23% in metastatic MM from the KEYNOTE-151 trial, we hypothesized a target pCR rate of 30% for this neoadjuvant combination therapy. The sample size was determined using a precision-based strategy, aiming for a 95% CI width of approximately 40% around the pCR estimate. Allowing for a 10% dropout rate, we targeted an enrollment of 26 patients, consistent with the exploratory nature of the study and the rarity of MM. As a post hoc sensitivity analysis, an exact one-sample binomial test (one-sided $\alpha = 0.05$) confirmed that a sample size of 25 would provide 80% power to detect a pCR rate of 30% against a null hypothesis of 10%.
Data exclusions	Key exclusion criteria of patient included any prior systemic anti-cancer treatment for melanoma, complications such as active bleeding, perforation, or the need for emergency surgery,pre-existing or coexisting other malignant tumors (excluding those curatively treated and disease-free for more than 5 years), any active or historical autoimmune disease or systemic immune suppression.<br>For WES, DSP, TCR-seq, and mIHC analysis, data exclusions were based on pre-established quality control criteria.
Replication	This study was a single-arm trial with a relatively small sample size. A large clinical multi-center randomized trial is required.
Randomization	This study was a single-arm trial with a relatively small sample size, and randomization was not relevant to our study.
Blinding	This study was a single-arm trial with a relatively small sample size, and blinding was not relevant to our study.

Reporting for specific materials, systems and methods

We require information from authors about some types of materials, experimental systems and methods used in many studies. Here, indicate whether each material, system or method listed is relevant to your study. If you are not sure if a list item applies to your research, read the appropriate section before selecting a response.

Materials & experimental systems

n/a	Involved in the study	
☐	☒	Antibodies
☒	☐	Eukaryotic cell lines
☒	☐	Palaeontology and archaeology
☒	☐	Animals and other organisms
☐	☒	Clinical data
☒	☐	Dual use research of concern
☒	☐	Plants

Methods

n/a	Involved in the study	
☒	☐	ChIP-seq
☒	☐	Flow cytometry
☒	☐	MRI-based neuroimaging

Antibodies

Antibodies used	DSP: S100 (NBP2-54426, Novus, 1:400), Pmel17 (NBP2-34638, Novus, 1:400), CD45 (13917, Cell Signaling, 1:100), Syto 13 (S7575, Thermo, 1:1000)<br>mIHC: Granzyme B (CST46890, Cell Signaling, 1:200), CD4 (ZM-0418, Zsbio, 1:200), Ki-67 (CST9027, Cell Signaling, 1:300), ICOS (CST89601S, Cell Signaling, 1:150), SOX10 (ZA-0624, Zsbio, ready-to-use), CD8A (CST70306, Cell Signaling , 1:200), CD31/Opal 620 (ab182981, Abcam, 1:2000), S100/Opal 570 (ab52642, Abcam, 1:1000), αSMA/Opal 520 (ab124964, Abcam, 1:300).
Validation	All antibodies are commercially available and validated by manufactures. The validation information is listed below:<br>S100 (NBP2-54426, Novus):<br>https://www.novusbio.com/products/s100b-antibody-s100b-1706r_nbp2-54426<br>Pmel17 (NBP2-34638, Novus):<br>https://www.novusbio.com/products/pmel17-silv-antibody-hmb45_nbp2-34638<br>CD45 (13917, Cell Signaling):<br>https://www.cellsignal.com/products/primary-antibodies/cd45-intracellular-domain-d9m8i-rabbit-monoclonal-antibody/13917<br>Syto 13 (S7575, Thermo):<br>https://www.thermofisher.cn/order/catalog/product/S7575<br>Granzyme B (CST46890, Cell Signaling):<br>https://www.cellsignal.com/products/primary-antibodies/granzyme-b-d6e9w-rabbit-monoclonal-antibody/46890<br>CD4 (ZM-0418, Zsbio):<br>http://www.zsbio.com/product/ZM-0418<br>Ki-67 (CST9027, Cell Signaling):<br>https://www.cellsignal.com/products/primary-antibodies/ki-67-d2h10-rabbit-monoclonal-antibody/9027<br>ICOS (CST89601S, Cell Signaling):<br>https://www.cellsignal.com/products/primary-antibodies/icos-d1k2t-rabbit-monoclonal-antibody/89601<br>SOX10 (ZA-0624, Zsbio):<br>http://www.zsbio.com/product/ZA-0624

CD8A (CST70306, Cell Signaling):
https://www.cellsignal.com/products/primary-antibodies/cd8-alpha-c8-144b-mouse-monoclonal-antibody/70306
CD31/Opal 620 (ab182981, Abcam):
https://www.abcam.com/en-us/products/primary-antibodies/cd31-antibody-epr17259-ab182981
S100/Opal 570 (ab52642, Abcam):
https://www.abcam.com/en-us/products/primary-antibodies/s100-beta-antibody-ep1576y-astrocyte-marker-ab52642
αSMA/Opal 520 (ab124964, Abcam):
https://www.abcam.com/en-us/products/primary-antibodies/alpha-smooth-muscle-actin-antibody-epr5368-ab124964

Clinical data

Policy information about clinical studies

All manuscripts should comply with the ICMJE guidelines for publication of clinical research and a completed CONSORT checklist must be included with all submissions.

Clinical trial registration	NCT04622566
Study protocol	The study protocol is available as Clinical Trial Protocol in the Supplementary Information file.
Data collection	The data were collected at Peking University Cancer Hospital (PKUCH) in Beijing, China. Between September 2021 and April 2023, 26 patients were enrolled in the study, with patient recruitment and data collection conducted over this period.<br>In this trial, the patients underwent a comprehensive baseline assessment at PKUCH, which involved the collection of demographic information, medical history, and disease characteristics prior to enrollment. Systematic physical examinations, alongside relevant laboratory and imaging tests, were conducted both pre- and post-treatment. Radiological evaluations of tumors—utilizing enhanced thoraco-abdominal CT scans and PET-CT—were performed at baseline and preoperatively.<br>Resected tumors underwent detailed pathological examination, and the regression of resected tumors was evaluated by assessing the percentage of residual viable tumor within the microscopically identified tumor bed (via routine hematoxylin and eosin [H&E] staining). All tissue selection and quality control procedures were overseen by PKUCH's institutional Quality Assurance & Control Committee (comprising 3 attending pathologists and 2 attending surgeons), with unanimous consensus required for the evaluation of each case.<br>Patients received postoperative surveillance consistent with PKUCH's clinical protocols: quarterly clinical assessments (including carcinoembryonic antigen testing), enhanced thoracoabdominal CT scans every 3 months throughout follow-up.
Outcomes	The primary endpoint of the study was pCR rate. The secondary endpoint of the study were 1-year RFS rate, OS, clinical response, major pathological response rate, surgical outcomes, and safety. The pathologic response rate was defined as the proportion of patients achieving pCR, near-pCR, or pPR in the resected specimen. Pathologic response rate was assessed in both the surgical cohort and the ITT population, with patients who did not undergo resection considered as non-responders for the primary endpoint analysis. Surgical specimens were centrally reviewed to assess pathological response. The pCR was defined as the absence of viable tumor cells in the resected specimen. Near-pCR was defined as ≤10% viable tumor cells, pPR as 11%–50% viable tumor cells, and pNR as >50% viable tumor cells in the tumor bed34. MPR was defined as ≤10% residual viable tumor cells in the resection specimen, comprising both pCR (0% viable tumor) and near-pCR (>0% to ≤10% viable tumor). EFS was defined as the time from treatment initiation to an event to the first occurrence of disease progression precluding surgery, recurrence after surgery, or death. All the secondary endpoints and biomarker analyses were pre-specified as exploratory rather than confirmatory. Postoperative surveillance included imaging every 12 weeks during the first year, every four months in the second year, and every six months from the third to fifth years, or until disease recurrence, death, or withdrawal from the study. Surgical outcomes will be reported separately and are not detailed in this manuscript. Safety was evaluated in all patients who received at least one dose of pembrolizumab or lenvatinib. Adverse events were continuously monitored and classified based on the National Cancer Institute's Common Terminology Criteria for Adverse Events (CTCAE), version 5.0.

Plants

Seed stocks	*Report on the source of all seed stocks or other plant material used. If applicable, state the seed stock centre and catalogue number. If plant specimens were collected from the field, describe the collection location, date and sampling procedures.*
Novel plant genotypes	*Describe the methods by which all novel plant genotypes were produced. This includes those generated by transgenic approaches, gene editing, chemical/radiation-based mutagenesis and hybridization. For transgenic lines, describe the transformation method, the number of independent lines analyzed and the generation upon which experiments were performed. For gene-edited lines, describe the editor used, the endogenous sequence targeted for editing, the targeting guide RNA sequence (if applicable) and how the editor was applied.*
Authentication	*Describe any authentication procedures for each seed stock used or novel genotype generated. Describe any experiments used to assess the effect of a mutation and, where applicable, how potential secondary effects (e.g. second site T-DNA insertions, mosiacism, off-target gene editing) were examined.*
