# Supplementary material for: A phase II peri-operative study of pembrolizumab plus lenvatinib for mucosal melanoma
Source: Nat Commun. 2026 May 16;17:6491. doi: 10.1038/s41467-026-73190-1 (PMC13376369; doi:10.1038/s41467-026-73190-1)
Supplement: Supplementary file 3 — Transparent Peer Review file [file 41467_2026_73190_MOESM3_ESM.pdf]

nature portfolio

Peer Review File

A phase II peri-operative study of pembrolizumab plus lenvatinib for mucosal melanoma

Corresponding Author: Professor Lu Si

This file contains all reviewer reports in order by version, followed by all author rebuttals in order by version.

Version 0:

Reviewer comments:

Reviewer #1

(Remarks to the Author)
The authors report a prospective phase II study investigating the clinical efficacy and underlying biological mechanisms of neoadjuvant pembrolizumab plus lenvatinib in mucosal melanoma patients with resectable disease. Overall 26 patients received treatment and 21 underwent surgery
The study is interesting but several points need to be addressed

1) The RFS was not significantly different in path responseders vs non-responders, how the authors could explain this finding when compared to CM? It's important to include CIs and p values when comparisons are resported
2) There is no TCR clonality evaluation against neoantigens, can the authors report some experiments?
3) The authors state that they found predictive and prognostic biomarkers, but with the single arm design the predictive role cannot be demonstrated
4) The inflamed phenotype on TME after neoadjuvant therapy is not different in responders vs no-responders, any data on specific TCR clonality? How can the authors demonstrate that a specific immune response is generated in both groups?
5) It's not clear to me how a longer treatment can improve prognosis as hypothesized by the authors in patients with a switch from colf to hot TME, tumour resident T cell clones data again should be included

Reviewer #2

(Remarks to the Author)
1. The sample size justification is precision-based (targeting a 40% CI width around an assumed 30% pCR rate). While acceptable for estimation, this approach limits the ability to draw firm conclusions about whether the regimen is sufficiently active for further testing. Unlike a formal Phase II design, it does not specify null and alternative response rates, type I error, or power, and therefore does not provide a go/no-go framework for clinical decision-making.

2. The primary endpoint is listed as “pathologic response rate.” Please clarify whether this refers specifically to pCR, or whether near-pCR and pPR were prespecified as contributing to the composite “pathological response rate.”

3. For patients who declined surgery due to personal preferences, how were they handled in the primary analyses? Treating them as non-responders in ITT is consistent with strict definitions but should be clearly described.

4. Please distinguish clearly between the intention-to-treat population (n=26) and the surgery population (n=21). In the abstract, presenting both can cause confusion. If ITT was the primary analytic set, then all main results should be based on ITT.

5. Define “event” in EFS clearly (progression, relapse, second malignancy, death, etc.). Ensure consistency with protocol.

6. Please clarify how circulating CD4+/CD8+ T cells are being positioned as prognostic or predictive biomarkers. The current data do not appear sufficient to justify this claim. To establish prognostic value, the biomarker should be shown to be independently associated with clinical outcomes, typically using multivariable models that adjust for known risk factors, and accompanied by measures of discrimination, calibration, and clinical utility. To establish predictive value, evidence of a biomarker–treatment interaction is required. Analyses based on small subgroups or descriptive trends are not adequate.

7. Multiple exploratory endpoints (RFS, EFS, DMFS, biomarker analyses) are presented without adjustment for multiplicity. Please acknowledge that p-values and associations should be interpreted cautiously. The study is underpowered for subgroup analyses (e.g., site-specific outcomes, biomarker-defined subgroups). These results should be clearly labeled as exploratory.

Reviewer #3

(Remarks to the Author)
The authors present the results of a phase II, single-arm, open label trial evaluating perioperative pembrolizumab and lenvatinib in patients with resectable mucosal melanoma which demonstrated modest but interesting clinical outcomes data, and includes the use of spatial transcriptomics to investigate the potential of T cell subsets as biomarkers of treatment response. The authors should be commended on the scope of their work given the rarity of mucosal melanoma, and their ambitious undertaking in applying such a resource-intensive technology at this scale, however the technology employed (DSP) does not allow spatial analysis beyond compartment level and the depth of the analysis presented here leaves somewhat to be desired. While this represents a significant contribution, there are several points that could benefit from further clarification:

Major:
1. The Methods section leaves a significant amount to be desired. For the spatial transcriptomics data, there very limited information provided about how the analysis was performed, how the phenotypes were generated beyond the software used.
2. Please clarify criteria for ROI inclusion, how many ROIs were included per case and compare that to the total number of potential ROIs over the analyzable section. Figure 2a could be a useful area to clarify this issue, as some of the ROIs in the diagram do not consist of tissue but also the whole tissue does not have gridded ROIs. ROI selection is critical in not generating biased data.
3. Do any of the samples include tumor-adjacent stroma? In the DSP analysis, mIF is used to determine if a given pixel is immune vs tumor, but the immune microenvironment is highly dependent on localization within the tumor vs the stroma vs the tumor stroma interface. Was any analysis performed which investigated these relationships?
4. Please define the cohort referred to here better: lines 162-164 "These prognostic associations were further validated in an independent cohort of advanced MM patients treated with first-line atezolizumab plus bevacizumab, where elevated infiltration of activated $CD4^{+}/CD8^{+}$ T cells associated with improved PFS and OS" - what is this cohort, how many patients were in this (looks like 16, but we are left without information about the provenance of this cohort), where is it from (a clinical trial?), and whether the same spatial transcriptomic methodology was applied.
5. Critically, this additional cohort could be used to contextualize the data from this trial in a much more convincing manner than presented here as it could highlight more comprehensively themes across combination trials for mucosal melanoma.

Minor:
1. We would recommend referring to this trial as a peri-operative trial, as the medical therapy both exists in the neoadjuvant phase as well as the adjuvant phase.
2. TRAEs were said to have been resolved with treatment interruption, were there incidences of multiple interruptions? Were any treated with immunosuppressive therapy?
3. It may be useful to give the reader an idea of the spread of patient the final AOIs were derived from. (Line 144-147)
4. Other than being both in the same compartment, did CD4+ or CD8+ exhibit any additional spatial arrangement changes? Did their proximity change relative to each other? To other immune cells?
5. If the immune composition did not change significantly for responders — what did change?
6. It is unclear to me how these immune composition change and immune cell recruitment pathways becoming upregulated in the non-responders leads to the conclusion that the therapy duration needs to be longer. Was there any comparison made to the responders that precipitated this thinking?
7. Comparison between responders and non-responders should be mentioned earlier than the discussion
8. Were attempts made to compare the relative gene expression strength between non-responders and responders?

Reviewer #4

(Remarks to the Author)
I co-reviewed this manuscript with one of the reviewers who provided the listed reports. This is part of the Nature Communications initiative to facilitate training in peer review and to provide appropriate recognition for Early Career Researchers who co-review manuscripts.

Version 1:

Reviewer comments:

Reviewer #1

(Remarks to the Author)
The authors addressed all my previous comments

Reviewer #2

(Remarks to the Author)
Thanks for taking the time to address my comments.
Most of my comments have been addressed. However, Comment #1 regarding the power/sample size rationale require further clarifications. Targeting a 40% CI width may yield a reasonably stable estimate, but it does not control the false-positive risk. Although the authors describe a precision-based sample size, the study design does not define a clinically meaningful benchmark (i.e., a null pCR rate) against which the observed ~30% pCR can be interpreted. It's unclear whether this reflects a true signal or estimation from a small cohort.

For all Kaplan–Meier figures, please add an at-risk table showing the number of patients at risk at the listed time points.

Reviewer #3

(Remarks to the Author)
The response to my review was thorough. From my perspective, it should be good to move forward and publish.

Open Access This Peer Review File is licensed under a Creative Commons Attribution 4.0 International License, which permits use, sharing, adaptation, distribution and reproduction in any medium or format, as long as you give appropriate credit to the original author(s) and the source, provide a link to the Creative Commons license, and indicate if changes were made.
In cases where reviewers are anonymous, credit should be given to 'Anonymous Referee' and the source.
The images or other third party material in this Peer Review File are included in the article's Creative Commons license, unless indicated otherwise in a credit line to the material. If material is not included in the article's Creative Commons license and your intended use is not permitted by statutory regulation or exceeds the permitted use, you will need to obtain permission directly from the copyright holder.
To view a copy of this license, visit https://creativecommons.org/licenses/by/4.0/

Point-by-point response:

We would like to sincerely thank the editor and the reviewers for their time and highly constructive comments, which have provided valuable insights to improve our study. We fully understand the concerns raised and the need for substantial revisions to strengthen our work. In response, we have carefully reviewed and comprehensively addressed all the comments.

The main changes to the manuscript are summarized below:

1. We have conducted TCR sequencing to compare TCR clonality to find specific immune response between different response groups (Reivewer 1).
2. We have clearly described the sample size calculation method, study endpoints, and statistical methods (Reivewer 2).
3. We have explicitly described the ROI inclusion criteria and bioinformatics methods, and we have improved the analysis of spatial transcriptomics data (Reivewer 3).
4. We have performed multiplex IHC to analyze the spatial arrangement and proximity changes of activated CD4 and CD8 T cells (Reivewer 3).

Below, we provide a point-by-point response to the reviewers' comments. The reviewers' comments are presented in bold ***italics***, and our responses are in plain blue text. All modifications made to the manuscript have been highlighted in yellow in the revised manuscript.

Reviewer #1 (Remarks to the Author):

The authors report a prospective phase II study investigating the clinical efficacy and underlying biological mechanisms of neoadjuvant pembrolizumab plus lenvatinib in mucosal melanoma patients with resectable disease. Overall 26 patients received treatment and 21 underwent surgery. The study is interesting but several points need to be addressed

1) The RFS was not significantly different in path responseders vs non-responders, how the authors could explain this finding when compared to CM? It's important to include CIs and p values when comparisons are resported.

Response: We thank the reviewer for this point. In the neoadjuvant setting in CM, pathological response is often defined using stringent criteria (pCR and near-pCR), and higher response rates typically provide clearer separation of outcome groups. In MM, pathological responses to neoadjuvant therapy are less frequent. In our cohort, only two patients achieved pCR and two achieved near-pCR, consistent with the only other neoadjuvant study in MM reported to date, which evaluated toripalimab plus axitinib and observed a pCR rate of 16.7% and a pathological response rate of 33.3%. In that study, responders also had a numerically longer RFS than non-responders (11.7 vs 6.2 months; p = 0.45). The absence of a statistically significant RFS advantage among pathological responders may also reflect the distinct biology of MM, which has a higher propensity for early systemic dissemination than CM. In addition, the small sample size and limited number of events lead to wide confidence intervals and reduced power to detect moderate survival differences. Larger studies with longer follow-up will be required to determine whether pathological response in MM confers a durable survival benefit comparable to that reported in CM. We have reorganized the Discussion to clarify this comparison with CM **(page 10, lines 278–289)**. We have also added 95% CIs and p values for the relevant comparisons throughout the clinical outcomes analyses.

2) There is no TCR clonality evaluation against neoantigens, can the authors report some experiments?

Response: We appreciate the reviewer's suggestion. We performed TCR sequencing and WES to assess intratumoral TCR clonality alongside tumor antigenicity-related features. TCR sequencing was performed on 8 pre-treatment biopsies (responders, n = 4; non-responders, n = 4) and 19 post-treatment surgical specimens (responders, n = 7; non-responders, n = 12) from 20 patients (responders, n = 8; non-responders, n = 12), including 7 paired pre- and post-treatment cases (responders, n = 3; non-responders, n = 4). In non-responders, clonotype counts and Shannon diversity increased after therapy, whereas the Simpson index and the contribution of top clonotypes decreased, indicating repertoire broadening **(Fig. 5a)**. To further characterize clonal remodeling, we classified intratumoral clonotypes as high-frequency (≥0.1%) or low-frequency (<0.1%) based on abundance **(Fig. 5b)**. Non-responders showed a significant increase in low-frequency clonotypes with a

decrease in high-frequency clonotypes, whereas responders showed the opposite pattern **(Fig. 5c)**, resulting in a higher post-treatment representation of high-frequency clonotypes in responders. In responders, high-frequency clonotypes increased while Shannon and Simpson index showed limited change, suggesting a more focused clonal expansion. In paired cases, the fraction of persistent clonotypes shared between pre- and post-treatment specimens was higher in responders than in non-responders **(Fig. 5d, e)**.

WES was performed on 9 pre-treatment biopsies (responders, n = 4; non-responders, n = 5) and 18 post-treatment surgical specimens (responders, n = 6; non-responders, n = 12) from 19 patients (responders, n = 7; non-responders, n = 12), including 8 paired cases. Predicted neoantigens were derived from WES; however, bulk TRB repertoire data did not allow inference of specific TCR–neoantigen pairings. We therefore examined the association between the number of high-frequency clonotypes and tumor neoantigen burden in post-treatment samples (n = 18) and did not observe a statistically significant association (Reviewer Fig. R1). We next assessed antigenicity using genomic features relevant to antigen presentation and immune escape **(Fig. 2)**. Notably, HLA loss of heterozygosity was observed exclusively in non-responders (4/12 vs. 0/7; p = 0.245), consistent with impaired antigen presentation as a potential resistance mechanism. These TCR and WES analyses have been incorporated into the revised manuscript **(page 6, lines 138–156; page 9, lines 245–264; Fig. 2 and Fig. 5)**.

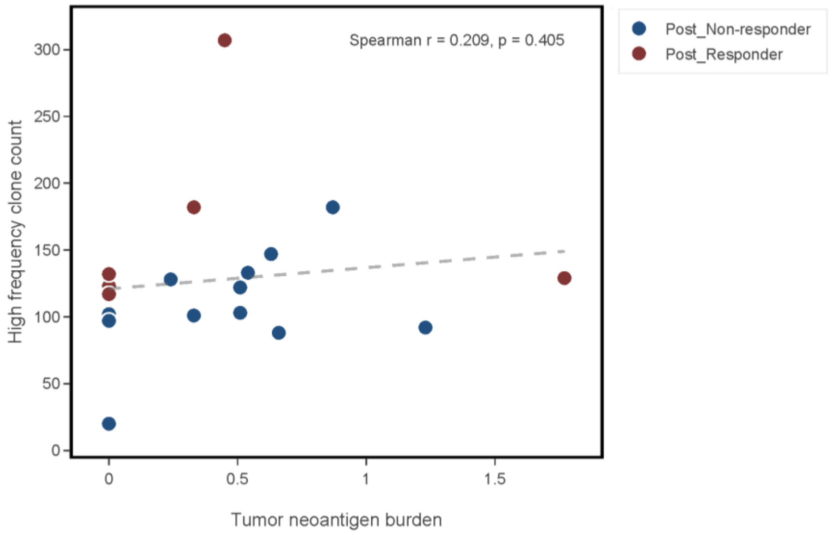

Reviewer Fig. R1. Spearman correlation between post-treatment high-frequency clonotype count and tumor neoantigen burden.

3) The authors state that they found predictive and prognostic biomarkers, but with the single arm design the predictive role cannot be demonstrated.

Response: We thank the reviewer for this important point and agree that a predictive biomarker effect cannot be concluded from our study design. We have therefore revised the manuscript to describe the activated $CD4^{+}/CD8^{+}$ T cell signature as a candidate outcome-associated biomarker in the setting of ICI plus

VEGF(R) inhibition. In support of this, the association between higher activated $CD4^+$/$CD8^+$ T cell signature scores and improved survival was observed in our independent, previously published multi-center phase II cohort of unresectable locally advanced or metastatic mucosal melanoma treated with first-line atezolizumab plus bevacizumab (NCT04091217; bulk RNA-seq available for 27 patients at our center), where higher signature scores were associated with improved PFS **(Fig. 3o, p)** and OS **(Fig. 3q, r)** **(page 8, lines 207–215)**. We also acknowledge in the Discussion that its predictive value needs to be validated in biomarker-stratified clinical trials **(page 13, lines 351–352)**.

4) The inflamed phenotype on TME after neoadjuvant therapy is not different in responders vs no-responders, any data on specific TCR clonality? How can the authors demonstrate that a specific immune response is generated in both groups?

Response: We appreciate the reviewer's comment. To assess whether treatment-associated T cell responses are generated and whether they differ by outcome, we performed intratumoral TCR sequencing as described above. Although both groups exhibited a more inflamed post-treatment microenvironment, TCR repertoire dynamics differed markedly between responders and non-responders. Responders showed preferential reinforcement of expanded and persistent clonotypes, consistent with a more focused clonal expansion pattern. In contrast, non-responders exhibited repertoire broadening characterized by an increase in low-frequency clonotypes and a reduced contribution of high-frequency/top clonotypes, suggesting a less focused expansion that may include bystander recruitment. Together, these findings indicate that, despite broadly increased inflammation after treatment, the underlying T cell expansion patterns are distinct—more focused and persistent in responders versus broader and low-frequency–dominated in non-responders. We have added this interpretation to the revised Discussion **(page 11, lines 311–316, and page 12, lines 323–326)**.

5) It's not clear to me how a longer treatment can improve prognosis as hypothesized by the authors in patients with a switch from cold to hot TME, tumour resident T cell clones data again should be included.

Response: We thank the reviewer for pointing out that this interpretation was unclear. Our DSP and mIHC analyses indicate that non-responders had a relatively "cold" baseline microenvironment. After two cycles of pembrolizumab plus lenvatinib, several immune infiltration–related features increased, and we observed vascular remodeling consistent with normalization. These on-treatment changes are consistent with partial immune priming and improved immune access to the tumor, and they motivated our hypothesis that a longer neoadjuvant exposure could further deepen immune remodeling in selected patients. However, this remains hypothesis-generating and requires prospective testing. Moreover, TCR sequencing in non-responders showed repertoire broadening dominated by low-frequency clonotypes, consistent with less focused clonal expansion. In light of this, we have

removed the treatment-duration hypothesis from the Abstract and revised the corresponding statements in the Discussion accordingly **(page 12, lines 326–332)**.

Reviewer #2 (Remarks to the Author):

1. The sample size justification is precision-based (targeting a 40% CI width around an assumed 30% pCR rate). While acceptable for estimation, this approach limits the ability to draw firm conclusions about whether the regimen is sufficiently active for further testing. Unlike a formal Phase II design, it does not specify null and alternative response rates, type I error, or power, and therefore does not provide a go/no-go framework for clinical decision-making.

Response: We thank the reviewer for this important point. We agree that a precision-based sample size provides an estimation framework rather than a formal Phase II go/no-go decision design. Given the rarity of mucosal melanoma and the exploratory intent of this single-arm neoadjuvant study, we adopted a precision-based rationale to obtain a reasonably precise estimate of the pathological response rate, rather than specifying null and alternative response rates with a pre-defined type I error and power. Specifically, based on prior response rates reported in advanced mucosal melanoma (ORR ~13–23%), we anticipated an approximately 30% pCR rate and targeted 26 patients to achieve an approximate 95% confidence interval width of ~40% around the estimated response rate. We have clarified this design choice in the Methods **(page 13, lines 370–373)**.

2. The primary endpoint is listed as "pathologic response rate." Please clarify whether this refers specifically to pCR, or whether near-pCR and pPR were prespecified as contributing to the composite "pathological response rate."

Response: We thank the reviewer for highlighting this important point and apologize for the lack of clarity. We have clarified that the primary endpoint was pCR. The pathologic response rate (PRR) was pre-specified as a composite of pCR, near-pCR, and pPR in the resected specimen **(page 14, lines 388–389)**.

3. For patients who declined surgery due to personal preferences, how were they handled in the primary analyses? Treating them as non-responders in ITT is consistent with strict definitions but should be clearly described.

Response: We thank the reviewer for raising this point. Five patients did not undergo surgery after neoadjuvant therapy, primarily due to patient preference. These patients were excluded from surgery-cohort analyses. For the ITT analysis of the primary endpoint, patients who did not undergo resection were classified as non-responders, consistent with a conservative ITT approach. We have now explicitly clarified this handling in the Methods as "Pathologic response rate was assessed in both the surgical cohort and the ITT population, with patients who did not undergo resection considered as non-responders for the primary endpoint analysis." **(page 14, lines 390–392)**.

4. Please distinguish clearly between the intention-to-treat population (n=26) and the surgery population (n=21). In the abstract, presenting both can cause confusion. If ITT was the primary analytic set, then all main results should be based on ITT.

Response: We agree that clear distinction between the ITT population and the surgical population is essential. Because five patients did not undergo resection and most subsequently lacked adequate follow-up, robust ITT survival analyses were not feasible. Accordingly, the main time-to-event analyses were performed in the surgical cohort (n = 21). To improve clarity, we have clarified the analytic populations in the Results and now report only the key outcomes for the surgical cohort in the Abstract **(page 2, lines 30–33)**.

5. Define "event" in EFS clearly (progression, relapse, second malignancy, death, etc.). Ensure consistency with protocol.

Response: We thank the reviewer for this point. We have now clarified the definition of EFS in the Methods as "EFS was defined as the time from treatment initiation to the first occurrence of disease progression precluding surgery, recurrence after surgery, or death." **(page 14, lines 396–397)**

6. Please clarify how circulating CD4+/CD8+ T cells are being positioned as prognostic or predictive biomarkers. The current data do not appear sufficient to justify this claim. To establish prognostic value, the biomarker should be shown to be independently associated with clinical outcomes, typically using multivariable models that adjust for known risk factors, and accompanied by measures of discrimination, calibration, and clinical utility. To establish predictive value, evidence of a biomarker–treatment interaction is required. Analyses based on small subgroups or descriptive trends are not adequate.

Response: We thank the reviewer for this important point. We agree that predictive value cannot be established without evidence of a biomarker–treatment interaction, and that prognostic claims should be supported by multivariable adjustment and independent validation. To avoid confusion, we clarify that the biomarker referred to in our manuscript is an activated $CD4^+/CD8^+$ T cell signature derived from tumor profiling, rather than circulating $CD4^+/CD8^+$ T cell measures. We have now performed multivariable Cox proportional hazards models to evaluate the association between this signature and time-to-event endpoints while adjusting for relevant clinical covariates **(Fig. 3l, n)**. In addition, we observed consistent survival associations in an independent, previously published mucosal melanoma cohort treated with atezolizumab plus bevacizumab (NCT04091217; bulk RNA-seq available for 27 patients at our center), where higher activated $CD4^+/CD8^+$ T cell signature scores were associated with improved PFS and OS **(Fig. 3o–r; page 8, lines 205–212)**. We have revised the manuscript to describe this signature as a candidate outcome-associated biomarker in the setting of ICI plus VEGF(R)

inhibition, and we acknowledge in the Discussion that its predictive value will require biomarker-stratified clinical trials **(page 13, lines 351–352)**.

7. Multiple exploratory endpoints (RFS, EFS, DMFS, biomarker analyses) are presented without adjustment for multiplicity. Please acknowledge that p-values and associations should be interpreted cautiously. The study is underpowered for subgroup analyses (e.g., site-specific outcomes, biomarker-defined subgroups). These results should be clearly labeled as exploratory.

Response: We thank the reviewer for this important point. We agree that the time-to-event endpoints (RFS, EFS, DMFS) and all biomarker analyses are exploratory in this single-arm phase II study and were not adjusted for multiple comparisons. As described above, we performed multivariable Cox proportional hazards model analyses. We further clarified in the Methods that all secondary endpoints and biomarker analyses were pre-specified as exploratory rather than confirmatory **(page 14, lines 397–398)**. In addition, we also added a limitation in the Discussion noting that the single-arm design and limited sample size reduce statistical power; accordingly, the findings should be interpreted cautiously as exploratory **(page 13, lines 342–344)**.

Reviewer #3 (Remarks to the Author):

The authors present the results of a phase II, single-arm, open label trial evaluating perioperative pembrolizumab and lenvatinib in patients with resectable mucosal melanoma which demonstrated modest but interesting clinical outcomes data, and includes the use of spatial transcriptomics to investigate the potential of T cell subsets as biomarkers of treatment response. The authors should be commended on the scope of their work given the rarity of mucosal melanoma, and their ambitious undertaking in applying such a resource-intensive technology at this scale, however the technology employed (DSP) does not allow spatial analysis beyond compartment level and the depth of the analysis presented here leaves somewhat to be desired. While this represents a significant contribution, there are several points that could benefit from further clarification:

Major:

1. The Methods section leaves a significant amount to be desired. For the spatial transcriptomics data, there very limited information provided about how the analysis was performed, how the phenotypes were generated beyond the software used.

Response: We appreciate the reviewer's comment and agree that the Methods section required additional detail for the spatial transcriptomics analysis, particularly regarding how immune phenotypes were generated. We have therefore expanded the Methods section (GeoMx digital spatial profiling) to explicitly describe the phenotype derivation and downstream analytical workflow. Briefly, raw digital

count conversion (DCC) files were normalized using ERCC RNA spike-in controls to mitigate system bias and adjust for ROI size variation, and ROIs were filtered using platform-recommended QC criteria, including exclusion of ROIs with normalization factors outside 0.3–3.0. QC-qualified ROIs were subsequently normalized using Q3 (third quartile) normalization and log-transformed.

For functional interpretation, differentially expressed genes ($|\log2$ fold change$| \geq 1$ and FDR < 0.05) were subjected to GO/KEGG enrichment analysis using Metascape (enrichment $p < 0.01$), and pathway-level enrichment was additionally assessed by GSEA using the R package clusterProfiler with MSigDB gene sets, applying an adjusted p-value < 0.05. Immune phenotypes were derived from curated gene expression signatures using ssGSEA within $CD45^+$ (immune compartment) ROIs, generating per-ROI immune cell signature scores. For heatmaps visualization, gene expression values were z-score standardized across samples. For statistical comparisons, paired pre- and post-treatment analyses were performed at the specimen level by averaging multiple ROIs from the same specimen (mean signature score). These methodological details have now been explicitly added to the Methods section **(page 16, lines 454–462)**.

2. Please clarify criteria for ROI inclusion, how many ROIs were included per case and compare that to the total number of potential ROIs over the analyzable section. Figure 2a could be a useful area to clarify this issue, as some of the ROIs in the diagram do not consist of tissue but also the whole tissue does not have gridded ROIs. ROI selection is critical in not generating biased data.

Response: We thank the reviewer for highlighting the importance of ROI selection and the potential for sampling bias in DSP studies. ROIs were selected on scanned sections using histology and immunofluorescence signals and were restricted to on-tissue regions within pathologist-annotated tumor and stroma compartments. ROIs were placed only within analyzable tissue and avoided off-tissue areas and regions with technical artifacts. ROIs were further required to meet the predefined DSP quality-control criteria described in the Methods (including minimum surface area and nuclei requirements and exclusion of ROIs with normalization factors outside 0.3–3.0).

To address the reviewer's request to report how many ROIs were included per case and to relate this to the overall analyzable section, we now quantify sampling using three indicators calculated on the analyzable tissue section (defined as the on-tissue tumor/stroma mask after excluding off-tissue regions and artifacts): the number of ROIs per case, ROI density (ROIs per mm^2 analyzable area), and ROI coverage (the sum of ROI areas divided by the analyzable tissue area). In our dataset, the median number of ROIs per case was 4 (IQR: 2–4) for pre-treatment biopsies and 4 (IQR: 3–6) for post-treatment resections. As expected, pre-treatment biopsies were generally smaller than post-treatment surgical specimens, leading to differences in absolute sampling across time points; however, within each time point, ROI number, density, and coverage were comparable between responders and non-responders,

suggesting no evidence of substantial, response-associated systematic sampling bias (Reviewer Fig. R2).

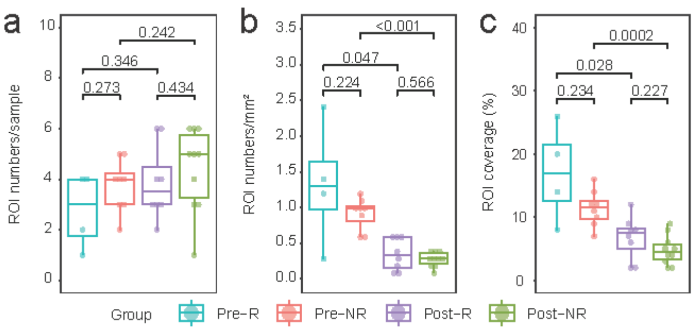

Reviewer Fig. R2. ROI sampling indicators: (a) number of ROIs per sample, (b) ROI density (ROIs per mm² analyzable tumor area), and (c) ROI coverage (sum of ROI areas divided by the analyzable tissue area).

We apologize for the confusion caused by the original DSP workflow schematic, in which some ROI circles may have appeared to extend beyond the tissue boundary or to include non-tissue areas. Importantly, ROIs were placed exclusively within pathologist-annotated, analyzable tissue regions, and no ROIs were positioned in off-tissue areas. We have revised the schematic in **Supplementary Figure S2a** to more clearly illustrate ROI placement relative to the analyzable tissue section.

We have now clarified the ROI inclusion and exclusion criteria in the Methods **(page 16, lines 441–443)**, added a dedicated description in the main text to mitigate the potential for ROI selection bias **(page 7, lines 166–170; Supplementary Fig. S2b-d)**, and revised the ROI schematic **(Supplementary Fig. S2a).**

3. Do any of the samples include tumor-adjacent stroma? In the DSP analysis, mIF is used to determine if a given pixel is immune vs tumor, but the immune microenvironment is highly dependent on localization within the tumor vs the stroma vs the tumor stroma interface. Was any analysis performed which investigated these relationships?

Response: We thank the reviewer for raising this important point regarding spatial localization of the immune microenvironment. In our study, tumor-adjacent stromal ROIs were available only in a limited subset of cases (12 specimens: 4 pre-treatment and 8 post-treatment). Within these specimens, we compared activated CD4 and activated CD8 T cell signature scores between tumor and stromal ROIs by averaging ROIs within each compartment per specimen and using the Wilcoxon test. We did not observe a statistically significant difference. Because stromal sampling was limited and this was not a primary planned analysis, we did not include these negative results in the main text. We have revised the Discussion to note limited stromal/interface coverage as a limitation and that addressing the tumor–stroma interface will require dedicated sampling with greater stromal representation **(page 13, lines 344–347)** .

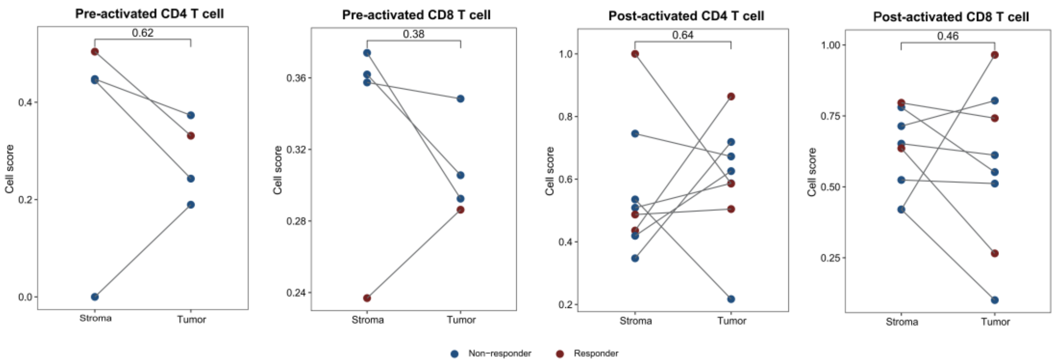

Reviewer Fig. R3. Comparison of activated CD4 and activated CD8 T cell signature scores between tumor and tumor-adjacent stromal ROIs in pre- and post-treatment specimens.

4.Please define the cohort referred to here better: lines 162-164 "These prognostic associations were further validated in an independent cohort of advanced MM patients treated with first-line atezolizumab plus bevacizumab, where elevated infiltration of activated CD4$^+$/CD8$^+$ T cells associated with improved PFS and OS" - what is this cohort, how many patients were in this (looks like 16, but we are left without information about the provenance of this cohort), where is it from (a clinical trial?), and whether the same spatial transcriptomic methodology was applied

Response: We thank the reviewer for pointing out that the provenance of the independent validation cohort should be described more clearly. This cohort was derived from a multi-center phase II study of first-line atezolizumab plus bevacizumab in advanced mucosal melanoma (NCT04091217), and both the clinical cohort and the associated transcriptomic profiling have been previously published. In brief, 43 patients were enrolled in the trial, and bulk RNA-seq data were available for 27 patients treated at our center. Activated CD4$^+$ and CD8$^+$ T cell scores were quantified from the bulk RNA-seq profiles using the same gene-signature definitions and scoring approach as in the neoadjuvant cohort. We have revised the manuscript to specify the cohort source, sample size, analytic methodology **(page 8, lines 207–212)**, and to add the appropriate citations for this validation dataset **(page 20, reference 22)**.

Reference: Dai J, *et al.* Atezolizumab plus bevacizumab in patients with unresectable or metastatic mucosal melanoma: 3-year survival update and multi-omics analysis. *Clin Transl Med* **15**, e70169 (2025).

5. Critically, this additional cohort could be used to contextualize the data from this trial in a much more convincing manner than presented here as it could highlight more comprehensively themes across combination trials for mucosal melanoma.

Response: We appreciate the reviewer's suggestion and agree that the previously published atezolizumab plus bevacizumab cohort provides an opportunity to place our findings in a broader mucosal melanoma combination-therapy context. We have

revised the Results **(page 8, lines 212-215)** and Discussion **(page 12, lines 333–338)** to more explicitly integrate the two cohorts, highlighting a consistent theme across ICI and VEGF(R) inhibition combination in mucosal melanoma: higher activated $CD4^{+}/CD8^{+}$ T cell scores are associated with more favorable clinical outcomes. We further revised the Discussion to place this finding within the biological rationale for combining ICI with VEGF(R) inhibition in mucosal melanoma, and to explain how an activated T cell milieu fits with the intended immunomodulatory effects of anti-angiogenic therapy **(page 12, lines 338–341)**.

Minor:

1. We would recommend referring to this trial as a peri-operative trial, as the medical therapy both exists in the neoadjuvant phase as well as the adjuvant phase.

Response: We thank the reviewer for this suggestion. We agree that, because systemic therapy was delivered both before and after surgery, "peri-operative" more accurately describes the overall trial framework. We have revised the manuscript to use "peri-operative" when referring to the study design and treatment strategy **(page 1, line 1; page 2, line 40; page 4, lines 79 and 84; page 10, line 268; page 13, lines 353 and 359)**. We then describe the regimen by phase to avoid ambiguity: neoadjuvant pembrolizumab plus lenvatinib followed by adjuvant pembrolizumab monotherapy.

2. TRAEs were said to have been resolved with treatment interruption, were there incidences of multiple interruptions? Were any treated with immunosuppressive therapy?

Response: We thank the reviewer for this question. Temporary treatment interruptions occurred primarily during the neoadjuvant phase and were mainly attributable to lenvatinib. In total, six patients had temporary interruptions for grade 1–2 TRAEs, and three of these resumed lenvatinib at a one-level dose reduction after recovery. No patient required more than once interruption. Following dose reduction, no further interruptions were required. Regarding immunosuppressive therapy, one patient with grade 3 ALT elevation received systemic corticosteroids. We have revised the manuscript to report these details explicitly **(page 5, lines 128–132)**.

3. It may be useful to give the reader an idea of the spread of patient the final AOIs were derived from. (Line 144-147)

Response: We thank the reviewer for this suggestion. We have revised the text to use the correct terminology and now consistently refer to these regions as ROIs (rather than AOIs). We also added a per-patient summary of ROI sampling to show how the profiled regions were distributed across patients, using three indicators: the number of ROIs per specimen, ROI density, and ROI coverage. These additions are described in the revised Results **(page 7, lines 164–170)** and summarized in

Supplementary Fig. S2. **Supplementary Table S1** lists the patients included in the GeoMx DSP analysis.

4. Other than being both in the same compartment, did CD4+ or CD8+ exhibit any additional spatial arrangement changes? Did their proximity change relative to each other? To other immune cells?

Response: We thank the reviewer for this comment and agree that changes in spatial organization may contribute to the different pathological responses between responders and non-responders. We performed mIHC using an ICOS/CD4/GZMB/CD8/Ki-67/SOX10 panel in 8 pre-treatment and 19 post-treatment samples. Due to panel size limitations, we focused the proximity analysis on activated T cell subsets and tumor cells. Activated $CD4^+$ T cells were defined as $ICOS^+CD4^+$ cells, activated $CD8^+$ T cells as $GZMB^+CD8^+$ cells, and proliferating melanoma cells as Ki-67^+SOX10^+ cells. The frequency and density of $ICOS^+CD4^+$ and $GZMB^+CD8^+$ cells increased after treatment in both responders and non-responders, whereas Ki-67^+SOX10^+ tumor cells decreased. Although between-group differences in the treatment-associated increases of $ICOS^+CD4^+$ and $GZMB^+CD8^+$ cells did not reach statistical significance, the magnitude of activated $CD4^+$ and activated $CD8^+$ T cell increases tended to be greater in post-treatment responders than in post-treatment non-responders. Spatial proximity analysis based on nearest-neighbor distances (NNC) showed that, within tumor core compartments, responders exhibited shorter NNCs between activated $CD4^+$ and $CD8^+$ T cells, as well as between these T-cell subsets and proliferating melanoma cells post-treatment, indicating a stronger immune–tumor cell interaction. These results have been added to the revised manuscript **(page 7, lines 191–203; Fig. 3e–j; Supplementary Fig. S3)**.

5. If the immune composition did not change significantly for responders — what did change?

Response: We thank the reviewer for point out that our description of treatment-associated changes in responders could be clearer and more complete. While overall immune composition differences in responders were modest, this may reflect the relatively high baseline immune infiltration in these cases. However, we observed several treatment-associated changes in responders. First, both DSP and mIHC showed increased activation-related T cell signals after treatment in responders, including higher activated $CD4^+$ and activated $CD8^+$ signatures and cell densities (Fig. 3a, b; Fig. 3e–h; Supplementary Fig. S3). Second, spatial proximity analysis demonstrated shorter nearest-neighbor distances between activated $CD4^+$ T cells, activated $CD8^+$ T cells, and melanoma cells after treatment, consistent with enhanced immune–tumor engagement (Fig. 3i, j). Third, pathway analyses in responders showed upregulation of immune-related programs, including T-cell receptor signaling, Toll-like receptor signaling, B-cell receptor signaling, and antigen processing and presentation (Fig. 3d). In addition, responders showed an increased representation of high-frequency clonotypes with limited changes in

Shannon and Simpson indicators, indicating a more focused clonal expansion (Fig. 5). We have revised the Discussion **(page 11, lines 301–316)** to more explicitly describe these responder-associated changes and to better balance the presentation between responders and non-responders.

6. It is unclear to me how these immune composition change and immune cell recruitment pathways becoming upregulated in the non-responders leads to the conclusion that the therapy duration needs to be longer. Was there any comparison made to the responders that precipitated this thinking?

Response: We thank the reviewer for pointing out that this interpretation was unclear. Our DSP and mIHC data show that non-responders exhibited a relatively "cold" baseline tumor microenvironment, yet several immune infiltration–related features and vascular remodeling signals increased after two cycles of combination therapy. These findings raise the possibility that a longer neoadjuvant course could further enhance immune remodeling and vascular normalization in selected patients, which will need to be tested in future studies. We did compare responders and non-responders in the Results; nevertheless, these data are not sufficient to support a treatment-duration recommendation. We have removed the the treatment-duration suggestion from the Abstract and revised the concluding statements in the Discussion accordingly **(page 12, lines 323–332; page13, 353–360)**.

7. Comparison between responders and non-responders should be mentioned earlier than the discussion

Response: We thank the reviewer for this suggestion. In the revised manuscript, we strengthened the responder versus non-responder comparisons in the Results by adding complementary assays and analyses. In addition to baseline differences in $CD45^+$ immune infiltration, we now include tumor-intrinsic comparisons in $S100/Pmel^+$ melanoma-cell ROIs at baseline. We also added mIHC and spatial proximity analyses, which provide histologic validation of the ssGSEA-derived immune signatures and demonstrate more pronounced treatment-associated increases in activated T cell abundance and tighter spatial proximity in responders compared with non-responders. In addition, we incorporated a dedicated TCR sequencing Results section with direct responder versus non-responder comparisons, showing repertoire broadening dominated by low-frequency clones in non-responders, whereas responders display higher persistence of clonotypes shared between pre- and post-treatment specimens. Collectively, these additions present response-associated differences more clearly in the Results.

8. Were attempts made to compare the relative gene expression strength between non-responders and responders?

Response: We thank the reviewer for this suggestion. We have now added a direct comparison between responders and non-responders at the same time point within tumor ROIs. Specifically, we performed differential expression analysis in pre-treatment tumor ROIs using the same normalization and statistical framework as

the main DSP analyses. Pathway analysis showed responders exhibited enrichment of antigen processing and presentation (NES = 2.54, adjusted $p < 0.001$) and IFN-γ response signaling (NES = 3.2, adjusted $p < 0.001$), together with higher expression of antigen-presentation and interferon-related transcripts, including *HLA-A*, *CD74*, *IRF1*, *STAT1*, and *ISG15* etc. These results have been added as **Fig. 4a–c** and are described in the revised Results **(page 8, lines 220–226)**.

Reviewer #4 (Remarks to the Author):

I co-reviewed this manuscript with one of the reviewers who provided the listed reports. This is part of the Nature Communications initiative to facilitate training in peer review and to provide appropriate recognition for Early Career Researchers who co-review manuscripts.

Response: We thank you for your time and effort.

Point-by-point response:

We would like to sincerely thank the editor and the reviewers for their time and valuable insights to improve our study. In response, we have carefully reviewed and comprehensively addressed these comments. Below, we provide a point-by-point response to the reviewers' comments. The reviewers' comments are presented in bold ***italics***, and our responses are in plain blue text. All modifications made to the manuscript have been highlighted in yellow in the revised manuscript.

Reviewer #1 (Remarks to the Author):

The authors addressed all my previous comments

Response: We thank you for your time and effort.

Reviewer #2 (Remarks to the Author):

1. Thanks for taking the time to address my comments. Most of my comments have been addressed. However, Comment #1 regarding the power/sample size rationale require further clarifications. Targeting a 40% CI width may yield a reasonably stable estimate, but it does not control the false-positive risk. Although the authors describe a precision-based sample size, the study design does not define a clinically meaningful benchmark (i.e., a null pCR rate) against which the observed ~30% pCR can be interpreted. It's unclear whether this reflects a true signal or estimation from a small cohort.

Response: We thank the reviewer for this insightful comment regarding the statistical rigor of our study design. We have clarified in the Methods that the trial used a precision-based sample size justification rather than a hypothesis-testing design, which is appropriate for an exploratory phase II trial in a rare malignancy such as mucosal melanoma. Specifically, the protocol assumed a target pCR rate of 30% and selected a sample size to achieve an approximately 40% two-sided 95% confidence interval width around the pCR estimate; 23 evaluable patients were required, and the final target enrollment of 26 allowed for 10% dropout.

We agree that this design does not by itself control false-positive risk against a prespecified null pCR rate, because no formal null benchmark or decision boundary was prespecified. To address the concern regarding the interpretation of the observed 30% pCR rate, we performed a sample size calculation using an exact one-sample binomial test, with one-sided $\alpha = 0.05$, and evaluating power at pCR rate

of 30%. A sample size of 25 would provide adequate power (80%) only for detecting a pCR rate of 30% against 10%.

null pCR rate	Rejection rule	Minimum sample size for 80% power
5%	Reject if ≥ 3 pCRs	14
**10%**	**Reject if ≥ 6 pCRs**	**25**
15%	Reject if ≥ 12 pCRs	48
20%	Reject if ≥ 31 pCRs	116

These additional quantitative results support the interpretation that the observed ~30% pCR rate represents a biologically plausible signal rather than random variation arising from a small cohort. We have clarified this design choice in the Methods **(page 14, lines 385–294)**. The R code used for both the precision-based and hypothesis-testing-based calculations has been deposited in our GitHub repository as referenced in the Code Availability statement.

2. For all Kaplan–Meier figures, please add an at-risk table showing the number of patients at risk at the listed time points.

Response: We have added at-risk tables showing the number of patients at risk at each time point for all Kaplan–Meier survival curves **(Figure 1d-g)**.

Reviewer #3 (Remarks to the Author):

The response to my review was thorough. From my perspective, it should be good to move forward and publish.

Response: We thank you for your time and effort.
